# Ovarian cycling and reproductive state shape the vaginal microbiota in wild baboons

**DOI:** 10.1186/s40168-017-0228-z

**Published:** 2017-01-19

**Authors:** Elizabeth A. Miller, Joshua A. Livermore, Susan C. Alberts, Jenny Tung, Elizabeth A. Archie

**Affiliations:** 1 Department of Biological Sciences, University of Notre Dame, Notre Dame, IN USA; 2 Institute of Primate Research, National Museums of Kenya, Nairobi, Kenya; 3 Department of Evolutionary Anthropology, Duke University, Durham, NC USA; 4 Duke Population Research Institute, Duke University, Durham, NC USA; 5 Department of Biology, Duke University, Durham, NC USA

**Keywords:** Vaginal microbiome, Primate, Reproductive state, Ovulation, Transmission

## Abstract

**Background:**

The vaginal microbiome is an important site of bacterial-mammalian symbiosis. This symbiosis is currently best characterized for humans, where lactobacilli dominate the microbial community and may help defend women against infectious disease. However, lactobacilli do not dominate the vaginal microbiota of any other mammal studied to date, raising key questions about the forces that shape the vaginal microbiome in non-human mammals.

**Results:**

We used Illumina sequencing of the bacterial 16S rRNA gene to investigate variation in the taxonomic composition of the vaginal microbiota in 48 baboons (*Papio cynocephalus*), members of a well-studied wild population in Kenya. Similar to prior studies, we found that the baboon vaginal microbiota was not dominated by lactobacilli. Despite this difference, and similar to humans, reproductive state was the dominant predictor of baboon vaginal microbiota, with pregnancy, postpartum amenorrhea, and ovarian cycling explaining 18% of the variance in community composition. Furthermore, among cycling females, a striking 39% of variance in community composition was explained by ovarian cycle phase, with an especially distinctive microbial community around ovulation. Periovulatory females exhibited the highest relative abundance of lactic acid-producing bacteria compared to any other phase, with a mean relative abundance of 44%. To a lesser extent, sexual behavior, especially a history of shared sexual partners, also predicted vaginal microbial similarity between baboons.

**Conclusions:**

Despite striking differences in their dominant microbes, both human and baboon vaginal microbiota exhibit profound changes in composition in response to reproductive state, ovarian cycle phase, and sexual behavior. We found major shifts in composition during ovulation, which may have implications for disease risk and conception success. These findings highlight the need for future studies to account for fine-scale differences in reproductive state, particularly differences between the various phases of the ovarian cycle. Overall, our work contributes to an emerging understanding of the forces that explain intra- and inter-individual variation in the mammalian vaginal microbiome, with particular emphasis on its role in host health and disease risk.

**Electronic supplementary material:**

The online version of this article (doi:10.1186/s40168-017-0228-z) contains supplementary material, which is available to authorized users.

## Background

The composition of the human vaginal microbiome varies considerably between individuals and within the same individual over time [1, 2]. Such variation is important because it can have major consequences for a woman’s vaginal health, disease risk, and fertility [3–5]. In contrast to humans, we know very little about the causes and consequences of inter-individual variation in the vaginal microbiomes of non-human mammals. Furthermore, human vaginal microbiomes differ considerably from those of other mammals, including other primates [6], raising key questions about whether the forces that shape the human vaginal microbiome are unique to humans or are shared with other primates or mammals. Answering this question is important to understanding both (i) the generalizability of factors that explain inter-individual variation in the vaginal microbiome in different species and (ii) the ways in which non-human primates can serve as useful models for human vaginal microbial communities.

Unlike other primates, the human vaginal microbiota is usually dominated by members of the genus *Lactobacillus,* which typically comprise 70% or more of resident bacteria [1, 2]. Lactobacilli dominance is important for understanding both the forces shaping the human vaginal microbiome as well as its hypothesized functional properties. Specifically, lactobacilli produce lactic acid from the breakdown products of glycogen (e.g., maltose) in vaginal fluid [7–13]. This reaction creates an acidic environment (pH ≤ 4.5) that is thought to protect women against sexually transmitted diseases (STDs) and inhibit the proliferation of opportunistic endogenous bacteria ([10, 14, 15], reviewed in [4]). Indeed, the loss of a lactobacilli-dominated community and subsequent increase in vaginal pH can lead to the overgrowth of anaerobic bacteria, referred to as bacterial vaginosis (BV), which is associated with infertility, preterm birth, maternal infections, and increased risk of STDs [3, 16–19].

In humans, a variety of endogenous and exogenous factors alter the relative abundance of *Lactobacillus* and vaginal microbial composition [20, 21]. In particular, estrogen stimulates the proliferation of the vaginal epithelium, increasing available glycogen in the vagina [22, 23]. As such, the bacterial composition of the vaginal microbiome is strongly affected by normal fluctuations in estrogen that occur during puberty and menopause, between reproductive states, and over the menstrual cycle (e.g., [24–26]). In particular, estrogen peaks during ovulation and this peak is linked to high relative abundance of *Lactobacillus* spp., low microbial diversity, low vaginal pH, and a stable bacterial community [2, 27, 28]. In addition to estrogen, sexual contact and exposure to maternal bacteria during birth may also influence the vaginal microbiome. During sexual contact, transmission of novel bacteria or neutralization of vaginal acidity by seminal fluid may impact the vaginal environment [29–33]. In support, sexual promiscuity is linked to greater instability in vaginal bacteria and increased risk of BV [20, 34, 35]. Additionally, close contact with the mother’s vaginal canal during birth leads to the vertical transmission of maternal vaginal bacteria to offspring [36–38].

**Fig. 1:**
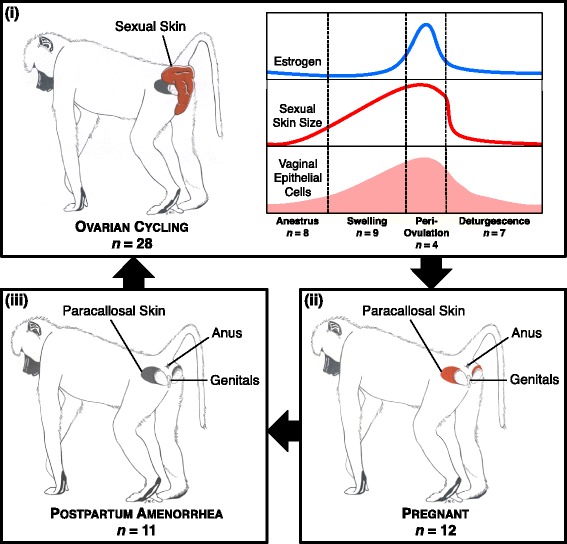
Schematic representing the progression through reproductive states and ovarian cycle phases in baboons with sample sizes for this study shown for each state/phase. *Baboon drawings* show the characteristics of the perineal sexual skin and the paracallosal skin associated with each reproductive state. Ovarian cycling includes four phases: (i) swelling (*n* = 9 samples), during which the perineal skin begins to swell; (ii) periovulation (*n* = 4 samples), which occurs at peak swelling, in the 5 days prior to deturgescence; (iii) deturgescence (*n* = 7 samples), when the perineal skin deflates; and (iv) anestrus (*n* = 8 samples), which occurs when the sexual swelling has completely deturgesced (anestrus includes days when females are menstruating). High levels of estrogen during periovulation stimulate the proliferation of the vaginal epithelium, increasing available glycogen in the vagina [22, 23]. If conception occurs during ovarian cycling, females become pregnant (*n* = 12), during which the color of their paracallosal skin changes from dark gray to pink [45]. After birth, the paracallosal skin of females in postpartum amenorrhea (those who either give birth or miscarry, prior to resumption of ovarian cycling; *n* = 11) gradually returns to dark gray. One sample collected from a female in the process of miscarrying is not included in the sample sizes shown in the figure

In contrast to humans, the vaginal microbiota of non-human primates (NHPs)—at least all NHPs studied to date—have few lactobacilli (typically <2% of resident bacteria) and much higher taxonomic diversity than humans [6, 21, 39–42]. Given these differences, it is unclear whether reproductive state, especially states linked to relatively high or low estrogen levels, will also be associated with predictable changes in NHP vaginal microbiota. Similarly, the effects of sexual contact or vertical transmission on NHP vaginal microbiota are not well understood. To address these gaps, we tested the association between reproductive state, ovarian cycle phase, sexual behavior, and vertical transmission and inter-individual differences in vaginal microbial composition in a well-studied population of wild baboons (*Papio cynocephalus*) living in the Amboseli ecosystem in Kenya. Similar to other NHPs, baboon vaginal microbiota are characterized by low levels of *Lactobacillus* spp., high microbial diversity, and high vaginal pH compared to humans [6, 39, 40, 43, 44]. Baboons also exhibit well-documented changes in external genital morphology that indicate female reproductive state and ovarian cycle phase, including pregnancy, postpartum amenorrhea, and four distinct stages of ovarian cycling (including ovulation; Fig. 1) [45–47]. However, like humans, baboons reproduce year-round: when not pregnant or in postpartum amenorrhea, baboons experience ~34-day ovarian cycles and can conceive at any time of the year [48, 49]. Also similar to humans, estrogen and vaginal glycogen are highest in baboons around ovulation and decrease during anestrus (i.e., menses)[46, 50–52]. Hence, if baboon vaginal microbiota experience dynamics similar to those in humans, we would expect to observe relatively high levels of lactic acid-producing bacteria during the periovulatory period and relatively low levels during low estrogen periods such as anestrus.

Our primary objective was to test the effects of reproductive state and ovarian cycle phase on the microbial composition of the baboon vaginal microbiome in order to understand whether the reproduction-related changes in baboon vaginal microbiota are parallel to those in humans. In addition, we used data on the baboons’ sexual contacts and maternal relationships to test for evidence of horizontal and vertical transmission in the vaginal microbiota. We also explored other variables that may shape inter-individual differences in baboon vaginal microbiota, including kinship, age, dominance rank, social group size (as a measure of available sexual partners), and rainfall (which may affect bacterial exposures in the environment). Finally, we measured vaginal pH in a separate set of subjects from the same population to understand the relationship between reproductive state and vaginal pH. To our knowledge, this data set represents the largest sample size and the greatest sequencing depth of the vaginal microbiota in a wild, non-human mammal to date. Our results improve our understanding of the factors that shape natural variation in the mammalian vaginal microbiome and provide valuable groundwork for further investigation on how vaginal microbiota contributes to host health and disease risk.

## Methods

### Study subjects

Study subjects for microbial analyses were 48 wild, adult female baboons (*Papio cynocephalus*), living in five different social groups in the Amboseli ecosystem in Kenya. Since 1971, this population has been intensively monitored by the Amboseli Baboon Research Project [53]. All study group members are individually recognized based on morphological characteristics. Experienced observers visit each study group two to three times per week, year-round, during 5-hour monitoring visits. During these visits, observers record information on a wide range of demographic, reproductive, and behavioral events, allowing us to correlate vaginal microbial composition with reproductive status (Fig. 1) and several other host traits, including history of sexual contacts, maternal relationships, kinship, age, dominance rank, social group size, and rainfall. Detailed information on data collection for each of these variables can be found in Additional file 1: Supplemental methods. A table describing all metadata for each sample can be found in Additional file 2: Table S1, with further information on ovarian cycle phase at the time of sample collection in Additional file 3: Figure S1.

### Sample collection, 16S rRNA gene sequencing, and sequence processing

We collected 52 vaginal swabs from 48 females between 2007 and 2010 (four individuals were sampled twice). Samples were collected from sexually mature females (range: 4–26 years; median: 8.5 years) that were anesthetized using an anesthetic bearing dart [54, 55]**.** Subjects were chosen opportunistically, but we excluded females in the second half of pregnancy and those with dependent infants (i.e., <270 days old). Once anesthetized, a sterile cotton swab was inserted into the female’s vaginal canal, gently rotated several times, and placed in 1 ml of RNAlater (Qiagen, Valencia, CA, USA).

Samples were stored at ambient temperature in the field and frozen at −20 °C upon arrival in the United States. Bacterial DNA was extracted from the swabs using the PowerSoil DNA Isolation kit (MO BIO Laboratories, Inc., Carlsbad, CA, USA), with modifications to the manufacturer’s instructions to accommodate the cotton swabs (see Additional file 1: Supplemental methods). A 102 base pair region of the 16S rRNA gene V4 region was amplified and sequenced using the methods developed by Caporaso et al. [56] with library preparation modifications by Davenport et al. [57]. Samples were multiplexed and sequenced in triplicate on three lanes of two Illumina HiSeq2000 flow cells (CA, USA).

Initial quality filtering was performed using PRINSEQ lite v.0.20.4 [58]. Sequences with ambiguous bases and/or with mean Phred quality scores ≤25 were discarded. To identify operational taxonomic units (OTUs), we employed open reference OTU picking. Specifically, representative sequences for first-round OTU-picking were chosen using USEARCH v.7.0 [59] with an initial OTU subsample depth of 2%. OTU clustering was completed using the UPARSE algorithm [60], which includes chimera filtering [59]. Sequences that did not match first-round OTUs were clustered and used as reference sequences in second-round OTU-picking. To reduce the risk of including OTUs that were PCR artifacts, all OTUs that occurred in only one sample were removed. Taxonomic identities were assigned to each OTU in QIIME v.1.8.0 [61] using the RDP Classifier v.2.2 [62] with the QIIME-formatted SILVA reference database (release 123, available at https://www.arb-silva.de/download/archive/qiime/) [63, 64]. Use of Greengenes [65] as the reference database produced qualitatively identical results, but with higher rates of unclassified OTUs. To verify that all sequences were from the 16S rRNA gene V4 region, reference sequences were aligned using the Python implementation of the NAST alignment algorithm (PyNAST)[66], and sequences that did not align were removed. To control for differences in sequencing depth between samples, we normalized read counts using cumulative-sum scaling implemented in the *R* package *metagenomeSeq* [67, 68]. This OTU table was used to calculate beta diversity and for differential relative abundance analyses. All analyses were repeated using an alternative OTU table rarefied to 1,777,373 reads per sample, and there were no qualitative differences in the results (data not shown).

### Statistical analyses

#### Predictors of alpha diversity

A full summary of all statistical analyses conducted in *R* is included in Additional file 4. Using the pre-normalized OTU table, we calculated the number of unique OTUs in a sample (i.e., richness) and Shannon’s diversity index, which accounts for the distribution of OTU abundances (Additional file 2: Table S1). To test predictors of microbial alpha diversity, we constructed multivariate linear regression models for both alpha diversity metrics in *R* (version 3.2.2, R Foundation for Statistical Computing, Vienna, Austria). We modeled the following as fixed effects: sequencing read count (to control for variation in sequencing depth between samples), age, reproductive state or ovarian cycle phase, dominance rank, presence or absence of rainfall in the 30 days prior to sample collection, the number of individuals in a female’s social group at sample collection (i.e., social group size), and level of promiscuity, estimated using the average number of consortship partners per ovarian cycle. Consortships are defined as sustained proximity between an adult male and female with a turgescent swelling [69]. Most sexual contact with intromission occurs in the context of consortships [54, 70, 71]. Although four individuals were sampled twice, we report models without individual identity because variation in alpha diversity among samples from the same female did not differ significantly from samples between females (Additional file 3: Figure S2). Furthermore, the inclusion of individual identity as a random effect in our models did not qualitatively change our results. Model selection was performed using stepwise backward regression with the *stepAIC* function from the *R* package *MASS* [72]. An alpha value of 0.05 was used as a threshold for inclusion in the final model.

#### Predictors of microbial beta diversity

To identify predictors of vaginal microbial similarity between samples, we performed principal coordinates analyses (PCoAs) and PERMANOVAs using Bray-Curtis dissimilarity and weighted UniFrac distance [73]. Predictor variables in the PERMANOVAs included age, reproductive state or ovarian cycle phase, dominance rank, rainfall, social group size, and level of promiscuity. Again, we did not include individual identity because variation in beta diversity between samples from the same female did not differ significantly from samples between females (Additional file 3: Figure S3).

#### Testing for horizontal and vertical transmission

To test for horizontal transmission, we used partial Mantel tests implemented in *R* package *vegan* [74] to correlate the extent to which females shared the same male consortship partners over their lifetime (partner sharing) with estimates of vaginal microbial community dissimilarity (i.e., Bray-Curtis and weighted UniFrac). Because similar sexual history between two vaginal samples could be due to the samples being from the same host, from hosts living in the same social group, or from hosts of similar age, we separately tested the correlations between microbial dissimilarity and each of the following matrices: a binary matrix of individual identity (i.e., samples from same or different individual), a binary matrix of social group co-residency (i.e., same or different social group), and a matrix of the absolute difference in ages between all pairs of sample dyads. For all partial Mantel tests, we controlled for reproductive state and ovarian cycle phase by including a third, binary matrix that indicated whether pairwise samples were from the same or different reproductive state or ovarian cycle phase. We calculated *P* values for all partial Mantel tests based on comparison of the observed Pearson correlation coefficient to Pearson coefficients calculated from 10,000 permutations.

To test for vertical transmission from mother to offspring, we used non-parametric Kruskal-Wallis tests to compare vaginal microbial dissimilarity between samples from maternal siblings (*n* = 10 sibling pairs) to the dissimilarity between samples from paternal siblings (*n* = 15 sibling pairs) and unrelated pairs of individuals (*n* = 1049). If vertical transmission plays a strong role in shaping female vaginal microbiota, microbial communities from maternal siblings should be significantly more similar to each other than the communities of both paternal siblings and unrelated dyads. To test the effects of pairwise genetic relatedness beyond mother-offspring effects on vaginal microbiota, we used partial Mantel tests to correlate a pedigree of both maternal and paternal relatedness with pairwise microbial dissimilarity, controlling for reproductive state.

#### Identifying taxa associated with different reproductive states and ovarian cycle phases

We used linear discriminant analysis (LDA) effect size (LEfSe; Galaxy v.1.0) to identify taxa that differed significantly in relative abundance between reproductive states and ovarian cycle phases. LEfSe is a method for high-dimensional biomarker discovery that combines standard statistical tests (i.e., the Kruskal-Wallis rank sum test and the pairwise Wilcoxon test) with linear discriminant analysis to detect taxa that explain the most variation between two or more classes [75]. We set the alpha value for the Kruskal-Wallis test at 0.01 and the threshold on the logarithmic LDA score at 3.0.

### Determination of vaginal pH

To understand the relationship between reproductive state and vaginal pH, we measured the vaginal pH of a separate set of 20 female Amboseli baboons between May and July of 2015 and 2016. None of these individuals were included in the vaginal microbiota portion of this study, and so we did not combine the pH data with microbial data for any analysis. However, all subjects for microbial and pH analyses came from the same wild population and lived together in the same five social groups, such that the pH data we collected should be representative of vaginal tract pH in our study system (as opposed to measurements from other species, populations, or captive animals). Measurements of pH were collected following Thoma et al. [24]. Briefly, a pH-Fix paper strip (pH 4.5–10.0, Macherey-Nagel, Düren, Germany) was affixed to a sterile pediatric tongue depressor and inserted into the vaginal canal with the aid of a speculum. The tongue depressor was kept in the vagina for 10 s and then the pH reading was taken immediately following removal.

## Results

### Baboon vaginal microbiota consist of a core set of taxa not dominated by *Lactobacillus* spp.

**Fig. 2:**
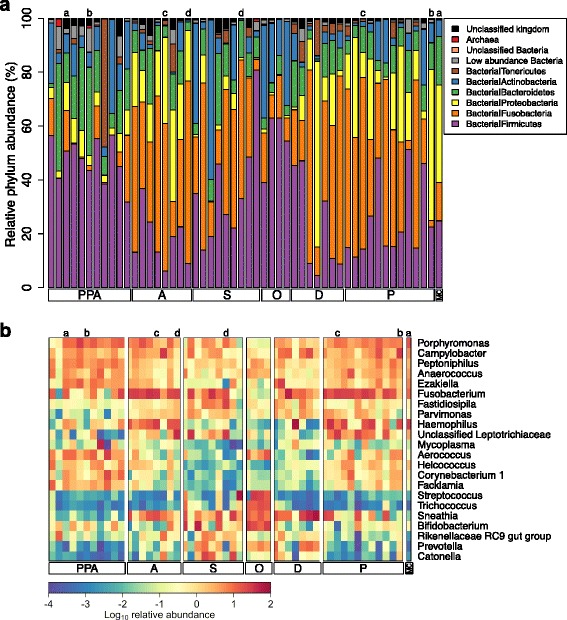
Relative abundance of bacterial and archaeal (**a**) phyla and (**b**) genera in the baboon vaginal microbiota by sample (*n* = 52). In panel **a**, each *bar* represents one sample; *colors* depict the proportion of reads assigned to a particular phylum in each sample. Only phyla with ≥1% mean relative abundance are shown. Phyla present at <1% are grouped into the category “Low abundance Bacteria”. Panel **b** shows a heatmap of the log_10_-transformed relative abundance of bacterial genera. Only genera with ≥1% mean relative abundance are shown. Each column represents one sample. Genera along the *y* axis were ordered based on hierarchical clustering by Euclidian distance. For both panels **a** and **b**, samples are ordered based on female reproductive state and ovarian cycle phase (Fig. 1): *PPA*, postpartum amenorrhea; *A*, anestrus; *S*, swelling; *O*, periovulation; *D*, deturgescence; *P*, pregnant; and *MC*, miscarrying. Matching *lower case letters* above bars and columns denote samples from the same individual

We generated a total of 188,665,626 reads from 48 female baboons (*n* = 52 samples; 1,777,373–7,024,839 reads per sample; mean = 3,628,185; Additional file 2: Table S1). Taxonomic assignments revealed representatives of 29 bacterial and archaeal phyla (Fig. 2a). Of these 29, 11 phyla were found in 100% (52/52) of samples, six of which had a mean relative abundance greater than 1%, including: *Firmicutes* (33%), *Fusobacteria* (29%), *Proteobacteria* (13%), *Bacteroidetes* (11%), *Actinobacteria* (10%), and *Tenericutes* (3%) (Fig. 2a; Additional file 2: Table S2). Forty-three genera were also found in all 52 samples, including multiple genera that have been linked to BV in humans, such as *Mobiluncus*, *Atopobium*, *Prevotella*, *Mycoplasma*, and *Sneathia* (Fig. 2b; Additional file 2: Table S2)[76].

As found in prior studies (e.g., [6, 43, 44, 77]), the baboon vaginal microbiota had markedly lower levels of lactobacilli than human vaginal microbiota. *Lactobacillus* spp. occurred in only 85% (44/52) of baboon samples, as compared to near 100% prevalence in women (e.g., [1]). Moreover, the median relative abundance of *Lactobacillus* spp. was only 0.00063% in baboons (range: 0–0.93%), compared to a reported median relative abundance of 96% in humans (range: 0%–99.9%) [1]. However, other lactic acid-producing bacteria (LAB), including *Streptococcus*, *Facklamia*, *Aerococcus,* and unclassified members of the order *Lactobacillales*, were relatively abundant in all baboon samples, with a mean relative abundance of 10% (±16% SD; Fig. 2b; Additional file 2: Table S2). Finally, approximately 12% of OTUs, comprising approximately 1.5% of reads, could not be classified beyond the kingdom level, suggesting that a large number of novel taxa colonize the baboon vagina. These unclassified OTUs were unlikely to be sequencing artifacts as all appeared in more than one sample, and the distribution of their prevalence across samples closely resembled the distribution of classified OTUs.

### The composition of vaginal microbiota changes with female reproductive state and ovarian cycle phase

#### Alpha diversity

**Fig. 3:**
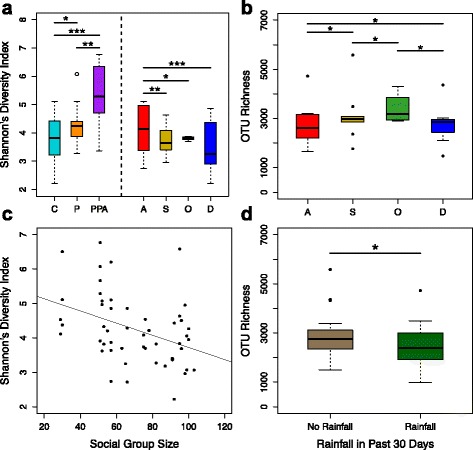
Plots summarizing major results of best supported multivariate linear regression models predicting Shannon’s diversity index and OTU richness (Additional file 3: Tables S3 and S4). **a** Shannon’s diversity index versus reproductive state (*n* = 51) and ovarian cycle phase (*n* = 28). **b** OTU richness versus ovarian cycle phase (**c**) Shannon’s diversity index versus social group size (**d**) OTU richness versus rainfall in the previous 30 days. *C*, ovarian cycling; *P*, pregnant; *PPA*, postpartum amenorrhea; *A*, anestrus; *S*, swelling; *O*, periovulation; *D*, deturgescence. *P ≤ 0.05; **P < 0.01; ***P < 0.001

Female reproductive state and ovarian cycle phase were strongly correlated with Shannon’s diversity, but not with OTU richness (Fig. 3a; Additional file 2: Table S3). Shannon’s diversity was lowest in cycling females and highest in females experiencing postpartum amenorrhea (PPA), with some evidence for increasing Shannon’s diversity throughout the first half of pregnancy (Fig. 3a; Additional file 2: Table S3; Additional file 3: Figure S4). Among the 28 females experiencing ovarian cycling (i.e., anestrus, swelling, periovulation, or deturgescence), anestrous females had the highest Shannon’s diversity (Fig. 3a; Additional file 2: Table S4), while OTU richness was higher in both anestrous and periovulatory females compared to swelling and deturgescent females (Fig. 3b; Additional file 2: Table S4).

Aside from reproductive state and ovarian cycle phase, we also found that social group size and rainfall in the 30 days prior to sample collection predicted microbial alpha diversity. Females who were members of larger social groups had lower Shannon’s diversity, compared to those in smaller groups (Fig. 3c; Additional file 2: Tables S3 and S4). This pattern may be caused by higher rates of intromission, and hence more disruption of the vaginal microbiota of females in larger versus smaller social groups. In support, females in larger social groups experienced more consortships and consortship partners per ovarian cycle than females in smaller groups (GLMMs: consortships: *z* value = 10.17, *P* < 10^−16^; consortship partners: *z* value = 5.47, *P* = 4.62 × 10^−8^; Additional file 3: Figure S5). Additionally, females sampled in rainy months had significantly lower OTU richness than females sampled in dry months, suggesting that the external environment affects vaginal exposure to bacteria (Fig. 3d; Additional file 2: Table S3). No other factors, including female age, dominance rank, or level of promiscuity predicted variation in either alpha diversity metric.

#### Beta diversity

**Fig. 4:**
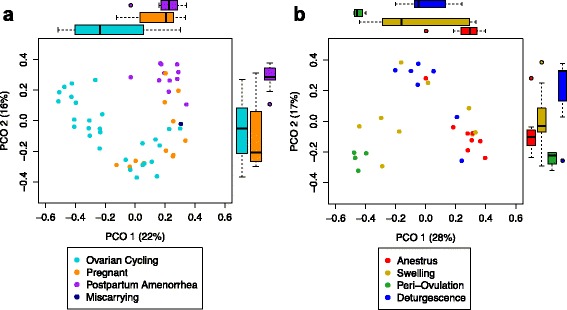
Principal coordinates plots based on Bray-Curtis dissimilarities for vaginal microbial communities as a function of (**a**) reproductive state (*n* = 52) and (**b**) ovarian cycle phase (*n* = 28). *Boxplots* are included to help visualize differences between reproductive states and ovarian cycle phases across principal coordinates axes 1 and 2

Female reproductive state and ovarian cycle phase were also strongly correlated with the composition of vaginal microbiota (Fig. 4; Additional file 3: Figure S6). Indeed, differences in reproductive state explained 18 to 19% of the variation in overall community composition (Fig. 4a; Additional file 3: Figure S6A; PERMANOVAs: Bray-Curtis: Pseudo*-*F = 5.24, *P* < 10^−5^; weighted UniFrac: Pseudo*-*F = 5.71, *P* < 10^−5^). Among the 28 samples collected during ovarian cycling, cycle phase explained 22 to 39% of the variation in microbial composition (Fig. 4b; Additional file 3: Figure S6B; PERMANOVAs: Bray-Curtis: Pseudo*-*F = 5.09, *P* < 10^−5^; weighted UniFrac: Pseudo*-*F = 2.31, *P* = 0.0032). Of particular interest, periovulation, as compared to swelling, anestrus, and deturgescence, was associated with a distinct vaginal microbiota, with significant differentiation along both the 1st and 2nd principal coordinate axes of the PCoA plot (green dots in Fig. 4b; Additional file 3: Figure S6B).

### Bacterial taxa, including some linked to bacterial vaginosis, vary in their relative abundance across female reproductive states

**Fig. 5:**
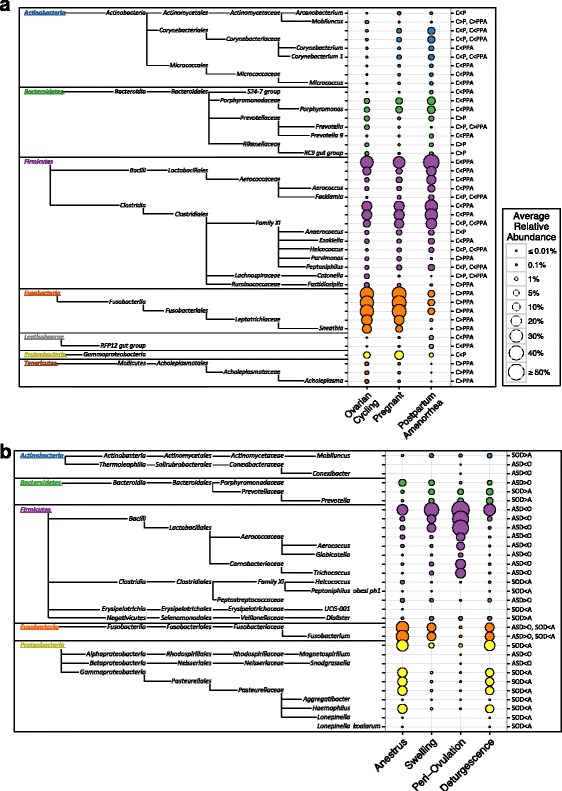
*Bubble plots* representing all vaginal bacterial taxa that have significant differences in relative abundance between reproductive states and ovarian cycle phases as identified by LEfSe. Pairwise comparisons included (**a**) ovarian cycling versus pregnancy (C vs. P), ovarian cycling versus postpartum amenorrhea (C vs. PPA), (**b**) periovulation versus anestrus, swelling, and deturgescence (O vs. ASD), and anestrus versus swelling, periovulation, and deturgescence (A vs. SOD). *Circle size* is proportional to each taxon’s average relative abundance and *circle color* differentiates between phyla. Significant comparisons (*P* ≤ 0.01) are noted on the right side of the plot

Given the striking correlation between reproductive state and community composition, we used LEfSe to identify which taxonomic groups experienced the largest changes in relative abundance when females transition from one reproductive state to another. We first compared females in the ovarian cycling phase to those in the next reproductive phase, pregnancy, and likewise compared females in late PPA to those in ovarian cycling. Of the 29 phyla tested, taxa from 7 phyla (24%) showed significant changes in relative abundance in at least one of the two reproductive state comparisons (*P* ≤ 0.01; Fig. 5a). Compared to both pregnant females and those in PPA, cycling individuals had elevated levels of common BV-associated genera, including *Sneathia*, *Prevotella*, and *Mobiluncus* [76]. Conversely, cycling females had relatively low levels of multiple other taxa, including the genus *Cornebacterium 1*, the LAB family *Aerococcaceae*—particularly from the genera *Facklamia* and *Aerococcus*—and multiple genera from the *Clostridiales* family XI. Furthermore, there appeared to be a negative relationship between the two dominant phyla *Firmicutes* and *Fusobacteria* across reproductive states, with cycling females exhibiting high levels *Fusobacteria* and low levels of *Firmicutes*, and PPA females displaying the opposite trend (Fig. 5a).

### The periovulatory period is associated with elevated *Lactobacillales*

Because community composition varied within the phases of the ovarian cycle (Fig. 4b), we also used LEfSe to identify the microbial taxa that exhibited the greatest changes in relative abundance during periovulation or anestrus relative to other cycle phases. These two phases may exhibit particularly distinct microbial communities because they represent the periods of maximum and minimum estrogen and vaginal glycogen within the ovarian cycle [46, 50–52]. Overall, samples from periovulatory females exhibited significantly higher relative abundance of *Bacilli*, especially members of the lactic acid-producing order *Lactobacillales* (mean: 44 ± 10% SD), and significantly lower relative abundance of the family *Fusobacteriaceae*, as compared to the other three cycle phases (Fig. 5b). Indeed, there was a striking trade-off in relative abundance between *Firmicutes* and *Fusobacteria* throughout the ovarian cycle, with high levels of *Firmicutes* and low levels of *Fusobacteria* around ovulation and the opposite pattern at the deturgescence to anestrus transition (Additional file 3: Figure S8). Additionally, compared to swelling, periovulating, and deturgescing females, anestrous individuals had significantly higher levels of genera from the Clostridiales family XI, the phylum *Proteobacteria*—particularly from the genus *Haemophilus*—and lower levels of the BV-associated genera *Mobiluncus* and *Prevotella* (Fig. 5b).

### Vaginal pH fluctuates over the ovarian cycle

The unique taxonomic composition associated with periovulation, especially the high relative abundance of lactic acid-producing bacteria, suggests that vaginal pH might be lowest in the time period around ovulation. To test the relationship between reproductive state and vaginal pH, we measured vaginal pH in a separate set of 20 female baboons living in the same social groups as the original subjects. Vaginal pH varied significantly across the reproductive states (pH range: 5.5–9.0), with the lowest vaginal pH in females with swollen, turgescent sexual skins, and the highest vaginal pH in females experiencing pregnancy and postpartum amenorrhea (Kruskal-Wallis test: *H* = 11.78, *P* = 0.019; Additional file 3: Figure S8). This finding suggests that vaginal pH fluctuates across the ovarian cycle, with pH falling to more acidic levels as females approach ovulation.

### Sharing sexual partners predicts vaginal microbial similarity, but vertical transmission does not

While reproductive state and ovarian cycle phase exerted dominant effects on vaginal microbiota, we also tested for evidence of horizontal and vertical transmission in shaping vaginal microbial communities. In the case of horizontal transmission, we predicted that females who share the same sexual partners should exhibit more similar vaginal microbiota than females who do not share sexual partners. In support, we found that females with more overlap among partners in their lifetime consortship profiles tended to have slightly more similar vaginal microbial communities, controlling for reproductive state and ovarian cycle phase, than females with less overlap among partners (partial Mantel tests: Bray-Curtis dissimilarity: *r* = 0.052, *P* = 0.046; weighted UniFrac: *r* = 0.043, *P* = 0.076; Additional file 2: Table S5). This pattern could not be explained by individual identity, similarity in age, or social group co-residency (Additional file 2: Table S5).

With respect to vertical (mother-offspring) transmission, we predicted that maternal siblings would exhibit more similar vaginal microbiota than paternal siblings or unrelated females. However, we found no evidence that vertical transmission affects the microbial composition of the baboon vaginal cavity in adulthood. Controlling for female reproductive state and ovarian cycle phase, pairwise samples from maternal siblings did not have more similar vaginal microbial communities than samples from paternal siblings or unrelated individuals (Kruskal-Wallis tests: Bray-Curtis dissimilarity: *H* = 0.25, *P* = 0.88; weighted UniFrac: *H* = 0.77, *P* = 0.68). We also found no evidence that overall pairwise genetic relatedness between all the female baboons in our data set explained similarity in vaginal microbial communities (partial Mantel tests: Bray-Curtis dissimilarity: *r* = 0.015, *P* = 0.30; weighted UniFrac: *r* = 0.018, *P* = 0.26 Additional file 2: Table S5).

## Discussion

To date, the majority of research on the vaginal microbiome of non-human primates has used captive animals (e.g., [6, 39, 41–43, 78]). Recently, several authors have called for greater use of wild subjects in studies of the microbiome, in part because of strong effects of captivity on host-associated microbial communities [79–81]. The study described here is, to our knowledge, only the third to investigate vaginal microbiota composition in wild primates; notably, the previous two studies were restricted to small sample sizes and lacked of information on host reproductive state [6, 77]. Thus, this work represents the most comprehensive investigation of inter-individual variation in the vaginal microbiota in a wild, non-human primate population.

All primates studied to date, in either captive or wild settings, lack the *Lactobacillus* spp. dominance typically found in human vaginal microbiota, raising questions about whether the forces that shape the human vaginal microbiome are also important in other primates and mammals. Despite low relative abundance of lactobacilli, we found that many of the same predictors of human vaginal microbiota are also important in wild baboons. Specifically, reproductive state and ovarian cycle phase—especially ovulation—were linked to distinct vaginal microbial communities, which may have consequences for functional aspects of the vaginal microbiome. In addition, females with similar sexual histories also had more similar vaginal microbiota. Together, these results suggest that, despite large differences in vaginal community composition, similar forces influence the communities of humans and non-human primates, laying important groundwork for further comparative work on how vaginal microbiota contribute to host health and disease risk across primates.

### Responses of the vaginal microbiota to host reproductive state and ovarian cycle phase

In humans, reproductive state (cycling, pregnancy, or postpartum amenorrhea) is the dominant force shaping the vaginal microbiome [26, 82–84]. These effects are primarily attributed to changing levels of estrogen, which affect the abundance of glycogen in vaginal mucus—a key resource for energy metabolism by lactobacilli [85]. Similar to humans, we found that reproductive state is also the primary driver of inter-individual variation in baboon vaginal microbiota, and many of the patterns we observe are consistent with the idea that fluctuations in estrogen and glycogen drive baboon vaginal microbial dynamics. For instance, like humans, glycogen is lowest in baboon vaginal mucus during both pregnancy and postpartum amenorrhea and highest during ovarian cycling [52]. Paralleling this pattern, the family *Aerococcaceae*, particularly the genus *Facklamia*, is prolific during pregnancy and PPA in female baboons, but rare during ovarian cycling. Since *Aerococcaceae* cannot metabolize glycogen directly and variably ferment maltose [86], the low relative abundance during cycling suggests this taxa is being outcompeted by other bacteria, such as *Sneathia* and *Prevotella*, that can metabolize glycogen directly and thus may thrive in a glycogen-rich environment (or, conversely, be more easily outcompeted by *Facklamia* in a glycogen-poor environment) [87, 88].

Within cycling female baboons, estrogen and vaginal glycogen fluctuate across ovarian cycle phases, with the highest levels around ovulation and lowest during anestrus [46, 50–52]. We find that the relative abundance of lactic acid-producing bacteria (LAB) follows a similar pattern, with an increase around ovulation and decrease during anestrus. Such changes are expected to reduce lactic acid production, and indeed, in baboons, we observe lower vaginal pH during the swelling phase of the ovarian cycle compared to anestrus. Similar patterns of vaginal pH have been observed over the human menstrual cycle, with a drop in pH during the follicular phase, and an increase during menses [27, 89].

It is worth noting that our findings differ from a prior study on the vaginal microbiota of cycling female baboons [43]. In contrast to our results, Uchihashi et al. [43] found no differences between females experiencing different ovarian cycle phases. There are two possible reasons for this finding. First, Uchihashi et al. studied captive baboons, and prior work has found that host-associated microbial communities can be altered by captivity [40]. Second, and probably more importantly, Uchihashi et al. did not make fine-scaled distinctions between different female ovarian cycle phases. For instance, Uchihashi et al. combined swelling, periovulatory, and deturgescence phases into a single category (“cycling”) and separated menstruating females from those that we termed anestrus (i.e., “non-cycling”). When we reanalyze our data based on Uchihashi et al.’s categories, we still observe significant differences in community composition between cycling and non-cycling females, but there were no significant differences in community composition between menstruating and non-menstruating females. Combining across cycle phases is common in the literature because it can be challenging to assign female mammals to the correct ovarian cycle phase. However, our results highlight the importance of these characterizations for understanding variation in the vaginal microbiota.

### The role of lactic acid-producing bacteria during ovulation

Consistent with previous work, we find that *Lactobacillus* is rare in the baboon vaginal microbiota, especially compared to humans [6, 39, 40, 43, 44]. From a functional standpoint, aspects of human physiology may make the human vagina more lactobacilli-friendly than other animals. For example, humans have significantly higher glycogen and lactic acid levels in vaginal fluid compared to macaques [90]. From an evolutionary perspective, differences in the vaginal microbiome between humans and NHPs may have arisen as a result of variation in sexual behavior and disease risk [6, 21]. Specifically, continual sexual receptivity and long intromission may expose humans to more pathogens (particularly STDs) compared to NHPs, leading to selection for a lactobacilli-dominated community [6, 21]. By extension, since STDs are not unique to humans, one might predict that NHPs should have the most protective microbial community when sexual activity and disease risk are greatest [91, 92]. For example, in baboons, the majority of sexual contact takes place within the second half of the swelling phase and periovulation [48]. Correspondingly, we observe the highest levels of LAB during this period, and find that BV-related bacteria are abundant during the periovulatory period, indicating that ovulation may be a time of high disease risk. In a separate data set, we also observe the lowest vaginal pH near this time; however, even the lowest vaginal pH measurements we recorded (pH = 5.5) were higher than what is considered protective and healthy in human women, whose vaginal pH typically ranges from 3.5 to 5.0 [1, 11, 85]. This suggests that defense mechanisms other than pH may also be at work in baboons, such as competitive exclusion, production of bacteriocins and other antimicrobial compounds [93, 94], and microbial interaction with the host immune system [95].

In addition to a possible protective function, the unique microbial community associated with the periovulatory phase may also have implications for sexual attraction. While male baboons typically identify sexually receptive females based on visual signals, such as sexual swellings [48], there is debate about how males choose one ovulating female over another (e.g., [96]). Vaginal microbiota may generate unique olfactory cues that signal ovulation, female ‘quality’, or likelihood of conception [97]. Indeed, work in a wide range of animals, including humans, suggests that many bacteria produce volatile compounds that can convey information about their hosts [98, 99]. For example, in humans, levels of volatile organic acids, including lactic acid, change over the human menstrual cycle [2], which may influence the attractiveness of female vaginal secretions [100].

### Transmission of vaginal bacteria

Past work in a wide range of animals indicates that microbial transmission occurs between sexual partners and from mother to offspring during birth [92, 101, 102]. While we find no evidence that vertical transmission exerts long-term effects on the vaginal microbiota of adult female baboons, our results do suggest that sexual contact leads to horizontal transmission of baboon vaginal bacteria. It is well established that copulation facilitates the spread of pathogens, and that multiple sexual partners can further increase the risk of infection [103, 104]. However, recent work suggests that commensal, and even beneficial, bacteria are also being transmitted between partners during sexual contact [102]. Thus, more promiscuous mating systems may promote exposure to these beneficial microbes [105]. In humans, there is some evidence for the sharing of commensal bacteria between sexual partners [30, 31], but, to our knowledge, our study provides the first evidence in a non-human primate that sharing sexual partners can result in similar commensal microbial communities. Interestingly, our results also suggest that an increased number of sexual partners may perturb the vaginal microbiota and reduce microbial alpha diversity. Indeed, in humans, promiscuity has been linked to greater instability in vaginal bacteria [20] and BV [106, 107]. However, it is yet to be determined whether these changes are due to the introduction of novel bacteria or neutralization of the vaginal environment due to repeated insemination [29].

## Conclusions

Like other non-human primates, baboons lack the *Lactobacillus* spp. dominance typically found in the human vaginal microbiome. Despite this difference and similar to humans, we found that reproductive state was the strongest predictor of the baboon vaginal microbiota, with effects on both microbial composition and pH. These results highlight the importance of accounting for fine-scale differences in reproductive state and emphasize the need for future studies to include this information whenever possible. Moreover, we found that during ovulation, the baboon vaginal microbiota exhibited traits reminiscent of those observed in humans, which are believed to defend against pathogens. This included the highest levels of lactic acid-producing bacteria, which predicts the lowest vaginal pH. Overall, our findings suggest that the vaginal microbiome of humans and baboons are under similar selective forces, which indicates these forces may be fundamentally important to many mammals. However, the unique nature of the human vaginal microbiome suggests that other factors shape vaginal microbial communities in humans. Future work should focus on disentangling the evolutionary pressures common to all mammals and those that are unique to humans.

## Additional files

Additional file 1: Supplementary methods. (DOCX 156 kb)
Additional file 2: Supplementary Tables S1-S5. (XLSX 65 kb)
Additional file 3: Supplementary Figures S1-S8. (DOCX 1129 kb)
Additional file 4: Full account of statistical analysis performed in *R*. (PDF 3480 kb)

## Abbreviations

A: Anestrus
BV: Bacterial vaginosis
C: Ovarian cycling
D: Deturgescence
LAB: Lactic acid-producing bacteria
LDA: Linear discriminant analysis
LEfSe: Linear discriminant analysis effect size
NHP: Non-human primate
O: Periovulation
OTU: Operational taxonomic unit
P: Pregnant
PCoA: Principal coordinates analysis
PPA: Postpartum amenorrhea
S: Swelling
STD: Sexually transmitted disease
