# Supplementary material for: Ovarian cycling and reproductive state shape the vaginal microbiota in wild baboons
Source: Microbiome. 2017 Jan 19;5:8. doi: 10.1186/s40168-017-0228-z (PMC5248513; doi:10.1186/s40168-017-0228-z)
Supplement: Additional file 1: — Supplementary methods. (DOCX 156 kb) [file 40168_2017_228_MOESM1_ESM.docx]

1. Data collection of host metadata
2. Age
3. Reproductive state
4. Dominance rank
5. Social group size
6. Rainfall
7. History of sexual contact
8. Patterns of kinship
9. DNA extraction from vaginal swabs
10. **Data collection of host metadata**

**1. Age**

All females in our data set were sexually mature and between 4 and 26 years old (median: 8.5 years). 47 of the 48 females had ages known to within a few days’ error. One female was born during a period of low monitoring of her study group, resulting in an age estimate accurate to within ±6 months.

**2. Reproductive state**

All females in the data set were sexually mature adults. Females were assigned to one of three reproductive states: ovarian cycling, pregnancy, or postpartum amenorrhea based on the characteristics of their perineal sexual skins, the color of their paracallosal skins, and the presence of external menstrual bleeding (Fig. 1)[1-4]. Females experiencing ovarian cycling (*n*=28 samples from 27 females) could be further divided into four phases: swelling, ovulation, deturgescence, and anestrus (Fig. 1, top-most box; Additional file 3: Fig. S1). Swelling corresponds to the second half of the follicular phase, an approximately two-week period during which a baboon's sexual skin fills with fluid. Ovulation has been shown to occur most often at the peak of the swelling phase, during the 5 days prior to the onset of deturgescence, which we term the peri-ovulatory period [4, 3]. Deturgescence occurs during the luteal phase, during which the female’s swelling decreases in size until flat. Anestrus is characterized by a completely deturgescent sexual skin. Menstruation occurs during the anestrous phase, although vaginal blood is not always observed.

Pregnant females (*n*=12) were identified by the cessation of ovarian cycling and a change in the color of their paracallosal skin from grey/black to pink (Fig. 1)[1]. All pregnant females in our data set were sampled in the first half of pregnancy. Pregnancy in baboons typically lasts 178 days [5, 6], and the number of days into pregnancy was calculated as the number of days since the onset of deturgescence during the previous ovarian cycle, which represented the most likely period of conception [4, 3].

Females in postpartum amenorrhea (*n*=11) were those that had either given birth or miscarried, but had not yet resumed ovarian cycling. To avoid disrupting close mother-infant contact, we did not collect samples from females with young infants; hence, all but one sample was collected >270 days after birth (one sample was collected eight days after a miscarriage). Some females in postpartum amenorrhea may have been lactating, which typically extends 1 to 1.5 years after parturition [7].

Finally, one sample was collected from a female in the process of miscarrying. This was determined based on: (i) her prior status as pregnant, (ii) the observation of blood on the day of sample collection, and (iii) the subsequent loss of pink color in her paracallosal skin. This sample was excluded from analyses involving reproductive state as a predictor variable, but was included in analyses that were not directly linked to reproductive state (i.e. the relative abundance of microbial taxa or prior sexual history).

**3. Dominance rank**

Ordinal dominance ranks were assigned to each female on a monthly basis using dyadic agonistic interactions observed during regular monitoring visits [8].

**4. Social group size**

To test for an effect of social group size on the vaginal microbiota, we used census data on the total number of individuals residing in each female’s social group on the day of sample collection.

**5. Rainfall**

The Amboseli ecosystem is characterized by highly seasonal patterns of rain, which may influence female condition [5, 9] and/or bacterial exposures from the environment [10, 11]. From June through October no rain falls. In the rest of year (November through May), the total rainfall is highly variable, ranging from less than 150 mm to more than 750 mm [12-14]. Samples were assigned one of two rainfall categories: 'rain’ if there had been rainfall in the 30 days prior to sample collection and 'no rain' if there had been no rainfall in the 30 days prior to sample collection. We chose to make rainfall a categorical variable since the data was mostly bimodal (depending on season of collection), with relatively extreme values.

**6. History of sexual contact**

To test for an effect of sexual contact on vaginal microbiota, we used measures of sexual partner diversity that could influence vaginal microbial alpha diversity, and a dyadic measure of sexual partner sharing, which could influence vaginal microbial similarity between females. To measure sexual partner diversity, we calculated the average number of unique consortship partners per ovarian cycle of a female’s adult life, up until sample collection. We refer to this as the females’ ‘level of promiscuity’. Observations of consortships are collected *ad libitum* during all monitoring visits.

To measure sexual partner sharing, we constructed a pairwise partner sharing matrix using the Bray-Curtis dissimilarity metric based on the identity of consortship partners and the number of consortships with each partner over a female's lifetime prior to sample collection. The dyadic Bray-Curtis dissimilarity values could thus range from 0 (for females who sexual history involved exactly the same males, with consortships distributed among these males in the same proportions) to 1 (for females whose sexual history involved a completely non-overlapping set of males). Two females who recently matured had never participated in a consortship and so were not included in this analysis. Bray-Curtis dissimilarities were calculated using the R package *vegan* [15].

**7. Patterns of kinship**

Finally, because vaginal microbial communities can be vertically transmitted between mothers and offspring [16], we used patterns of known maternal and paternal kinship to test whether maternal siblings harbored more similar vaginal microbiota than paternal siblings and ‘unrelated’ pairs of individuals. We constructed a relatedness matrix from known maternities and paternity assignments based on 14 microsatellite loci [17] and the R package *pedantics* [18]. In total, we had 10 maternal sibling pairs and 15 paternal sibling pairs in our data set. We considered dyads as “unrelated” if they had a coefficient of relatedness less than 0.125 (i.e. first cousins or more distantly related). The average coefficient of relatedness between unrelated dyads was 0.0045 (±0.015 SD).

**B. DNA extraction from vaginal swabs**

For DNA extraction, we used the Powersoil DNA Isolation kit (MO BIO Laboratories, Inc., Carlsbad, CA, USA), with modifications to the manufacturer’s instructions to accommodate the cotton swabs. Briefly, the cotton was cut from the swab using a sterile razor blade and placed in a clean 1.5 ml tube. To capture any bacterial DNA remaining in the RNAlater, we centrifuged the RNAlater at 14,000 x g for 10 minutes, removed the supernatant, and transferred the pellet to the tube containing the cotton. To lyse the bacterial cells, we added 500 μl of MoBio PowerBead buffer to the tube, vortexed the sample, and then transferred 500 μl of the supernatant and buffer mixture to the MoBio PowerBead tube for vortexing. DNA extraction then proceeded as described in the MoBio protocol. Extracted DNA was stored at -20°C.

**References**

1. Altmann SA. The pregnancy sign in savannah baboons. The Journal of Zoo Animal Medicine. 1973;4(2):8-12.

2. Gesquiere LR, Wango EO, Alberts SC, Altmann J. Mechanisms of sexual selection: Sexual swellings and estrogen concentrations as fertility indicators and cues for male consort decisions in wild baboons. Horm Behav. 2007;51(1):114-25.

3. Wildt DE, Doyle LL, Stone SC, Harrison RM. Correlation of perineal swelling with serum ovarian hormone levels, vaginal cytology, and ovarian follicular development during the baboon reproductive cycle. Primates. 1977;18(2):261-70.

4. Shaikh AA, Celaya CL, Gomez I, Shaikh SA. Temporal relationship of hormonal peaks to ovulation and sex skin deturgescence in the baboon. Primates. 1982;23(3):444-52.

5. Beehner JC, Onderdonk DA, Alberts SC, Altmann J. The ecology of conception and pregnancy failure in wild baboons. Behav Ecol. 2006;17(5):741-50.

6. Beehner JC, Nguyen N, Wango EO, Alberts SC, Altmann J. The endocrinology of pregnancy and fetal loss in wild baboons. Horm Behav. 2006;49(5):688-99.

7. Altmann J, Samuels A. Costs of maternal care: infant-carrying in baboons. Behav Ecol Sociobiol. 1992;29(6):391-8.

8. Hausfater G. Dominance and reproduction in baboons (*Papio cynocephalus*): a quantitative analysis. Contrib Primatol. 1975;7:2-150.

9. Gesquiere LR, Khan M, Shek L, Wango TL, Wango EO, Alberts SC et al. Coping with a challenging environment: Effects of seasonal variability and reproductive status on glucocorticoid concentrations of female baboons (*Papio cynocephalus*). Horm Behav. 2008;54(3):410-6.

10. Fierer N, Jackson RB. The diversity and biogeography of soil bacterial communities. P Natl Acad Sci USA. 2006;103(3):626-31.

11. Stumpf RM, Wilson BA, Rivera A, Yildirim S, Yeoman CJ, Polk JD et al. The primate vaginal microbiome: Comparative context and implications for human health and disease. Am J Phys Anthropol. 2013;152:119-34.

12. Alberts SC, Hollister-Smith J, Mututua RS, Sayialel SN, Muruthi PM, Warutere JK et al. Seasonality and long term change in a savannah environment. In: Brockman DK, van Schaik CP, editors. Seasonality in Primates: Studies of Living and Extinct Human and Non-Human Primates. Cambridge, UK: Cambridge University Press; 2005. p. 157-95.

13. Altmann J, Alberts SC, Altmann SA, Roy SB. Dramatic change in local climate patterns in the Amboseli basin, Kenya. Afr J Ecol. 2002;40(3):248-51.

14. Lea AJ, Altmann J, Alberts SC, Tung J. Developmental constraints in a wild primate. Am Nat. 2015;185(6):809-21.

15. Oksanen J, Blanchet FG, Kindt R, Legendre P, Minchin PR, O'Hara RB et al. vegan: Community Ecology Package. R package version 2.0-10 ed2013.

16. Funkhouser LJ, Bordenstein SR. Mom knows best: The universality of maternal microbial transmission. Plos Biol. 2013;11(8):1-9.

17. Alberts SC, Buchan JC, Altmann J. Sexual selection in wild baboons: From mating opportunities to paternity success. Anim Behav. 2006;72:1177-96.

18. Morrissey MB, Wilson AJ. pedantics, an R package for pedigree-based genetic simulation, and pedigree manipulation, characterisation, and viewing. Molecular Ecology Resources. 2010;10(4):711-9.
