# Supplementary material for: Ovarian cycling and reproductive state shape the vaginal microbiota in wild baboons
Source: Microbiome. 2017 Jan 19;5:8. doi: 10.1186/s40168-017-0228-z (PMC5248513; doi:10.1186/s40168-017-0228-z)
Supplement: Additional file 3: — Supplementary Figures S1-S8. (DOCX 1129 kb) [file 40168_2017_228_MOESM3_ESM.docx]

**Fig. S1.** Schema representing the ovarian cycle day and phase at the time of sample collection for all samples. Each diamond represents one sample. Diamond color denotes ovarian cycle phase at time of sample collection, while the number above each diamond indicates the day in the ovarian cycle. The onset of deturgescence is Day 0, with the peri-ovulatory phase occurring in the five preceding days (Days -5 through -1). The standard ovarian cycle lasts 34-days [1], although because the length of time an individual spends in a specific phase differs, females can be in different cycle phases on the same cycle day (e.g. both anestrous and swelling females were sampled on Day -20). Additionally, some females experience extended anestrous periods (e.g., the sample shown from an anestrous female on Day -87). One anestrous female died before resuming cycling and is not included in the figure.

**Fig. S2.** Variation in alpha diversity among samples from the same female did not differ significantly from samples between females. Box plots depict the difference in alpha diversity between samples from the same individual (*n*=4) and samples from different individuals (*n*=1,322). Plots depict (**A**) OTU richness (*W*=1,725, *P*=0.23) and (**B**) Shannon’s diversity index (*W*=2,876, *P*=0.76). The y-axes show the absolute difference in residuals from a multivariate linear regression with alpha diversity as the response variable and with read count and reproductive state or cycle phase as the fixed effects.

**Fig. S3.** Vaginal samples from the same individual are no more similar than samples from different individuals based on (**A**,**B**) Bray-Curtis dissimilarity (*W*=1,717, *P*=0.23), (**C**,**D**) weighted UniFrac distance (*W*=1,993, *P*=0.40). Panels **A** and **C** depict principal coordinates analyses of vaginal microbial communities with colors denoting samples from the same individuals. Panels **B** and **D** depict boxplots of beta diversity between samples from the same individual (*n*=4) and samples from different individuals (*n*=1,322). The y-axes shows the residuals from the linear regression with beta diversity as the response variable and reproductive state or cycle phase as the fixed effect.

**Fig. S4.** Shannon’s diversity of the baboon vaginal microbiota increases over the first half of pregnancy (*r*^2^=0.43, *F*(1,10)=7.4, *P*=0.021). The y-axes show the residuals from a linear regression with Shannon’s diversity index as the response variable and read count as a fixed effect.

**Fig. S5.** Females living in larger social groups engage in (**A**) more consortships with (**B**) more males. We used behavioral data on all mature females present within the Amboseli study population between 2007 and 2010. For each ovarian cycle of each female (i.e. beginning of the swelling phase to the end of the deturgescence phase), we calculated the number of observed consortships and the number of distinct consortship partners. This resulted in consortship data for 913 ovarian cycles from 117 unique females. The number of observations can depend on the number of observers concurrently watching a group and the number of times a group was visited. Thus, we controlled for differences in observation intensity by using a proxy of a group's true observer effort: the number of focal animal samples that occurred in the group during a female's ovarian cycle divided by the average group size during the same time period [see 2 for more information]. To test whether observations of mounts and number of mount partners was higher in larger social groups, we constructed generalized linear mixed models (GLMMs) with a Poisson distribution in R (version 3.2.2, R Foundation for Statistical Computing, Vienna, Austria). Observer effort and social group size were scaled and included as fixed effects and individual identity was included as a random effect. The y-axes from the above plots are the residuals from GLMMs including just observer effort as a fixed effect.

**Fig. S6.** Principal coordinates analysis of weighted UniFrac distance for vaginal microbial communities as a function of (**A**) reproductive state (*n*=52) and (**B**) ovarian cycle phase (*n*=28). Variation in vaginal microbial composition was explained by both reproductive state (PERMANOVA: Pseudo-F=6.92, *P*<10^-5^) and ovarian cycle phase (Pseudo-F=3.56, *P*=0.0006). Boxplots are included to help visualize differentiation between reproductive states and ovarian cycle phases across principal coordinates axes 1 and 2.

**Fig. S7.** Relative abundance of the bacterial phyla *Firmicutes* (purple circles) and *Fusobacteria* (orange triangles) over the standard (i.e. 34-day)[1] ovarian cycle (*n=*20). Lines show best fit polynomial functions (gray shaded areas=95% confidence interval).

**Fig. S8.** Vaginal pH of baboons (*n*=20) during pregnancy (P; *n*=5)**,** postpartum amenorrhea (PPA; *n*=2), and ovarian cycle phases (anestrus, A, *n*=3; swelling, S, *n*=3; deturgescence, D, *n*=7). Points are the mean pH for each reproductive state and cycle phase. Error bars represent the standard error of each state.

**References**

1. Gesquiere LR, Wango EO, Alberts SC, Altmann J. Mechanisms of sexual selection: Sexual swellings and estrogen concentrations as fertility indicators and cues for male consort decisions in wild baboons. Horm Behav. 2007;51(1):114-25.

2. Archie EA, Tung J, Clark M, Altmann J, Alberts SC. Social affiliation matters: Both same-sex and opposite-sex relationships predict survival in wild female baboons. P R Soc B. 2014;281(1793):1-9.
