# Supplementary material for: Ovarian cycling and reproductive state shape the vaginal microbiota in wild baboons
Source: Microbiome. 2017 Jan 19;5:8. doi: 10.1186/s40168-017-0228-z (PMC5248513; doi:10.1186/s40168-017-0228-z)
Supplement: Additional file 4: — Full account of statistical analysis performed in R. (PDF 3480 kb) [file 40168_2017_228_MOESM4_ESM.pdf]

Ovarian cycling and reproductive state shape the vaginal microbiota in wild baboons

E. A. Miller et al.

This document contains R code for all statistical analyses conducted for the manuscript as well as the R code to generate all figures.

Required data files:

All data files required to reproduce analyses will be made available on the Dryad Digital Respository ((http://datadryad.org/)http://datadryad.org/ (http://datadryad.org/)).

File Name	Description
otu_table.txt	OTU table with taxonomic information in last column (not normalized for read count)
otu_table_all.txt	OTU table with *all* OTUs (even those in only one sample)
rel_abundance_phylum.txt	phylum relative abundance by sample (created with Qiime script *summarize_taxa.py*)
rel_abundance_class.txt	class relative abundance by sample (created with Qiime script *summarize_taxa.py*)
rel_abundance_order.txt	order relative abundance by sample (created with Qiime script *summarize_taxa.py*)
rel_abundance_family.txt	family relative abundance by sample (created with Qiime script *summarize_taxa.py*)
rel_abundance_genus.txt	genus relative abundance by sample (created with Qiime script *summarize_taxa.py*)
rel_abundance_species.txt	species relative abundance by sample (created with Qiime script *summarize_taxa.py*)
metadata.txt	Sample metadata (including alpha diversity metrics)
all_consort.txt	Consortship and group size data on all ABRP adult female between 2007 and 2010

File Name	Description
bray_curtis_matrix.txt	Bray-Curtis dissimilarity matrix
weighted_unifrac_matrix.txt	Weighted UniFrac distance matrix
lefse_rep.txt	Results of reproductive states LEfSe analyses
lefse_cycle.txt	Results of ovarian cycle phases LEfSe analyses
vaginal_ph.txt	Vaginal pH measurements from a separate set of 20 female baboons with corresponding reproductive state or ovarian cycle phase
relatedness.txt	Relatedness matrix between baboons
consort_history.txt	Consortship history for female by sample

```
otu <- read.table("otu_table.txt", header=TRUE, row.names=1)
otu_all <- read.table("otu_table_all.txt", header=TRUE, row.names=1)
phylum <- read.table("rel_abundance_phylum.txt", header=TRUE, row.names=1)
class <- read.table("rel_abundance_class.txt", header=TRUE, row.names=1)
order <- read.table("rel_abundance_order.txt", header=TRUE, row.names=1)
family <- read.table("rel_abundance_family.txt", header=TRUE, row.names=1)
genus <- read.table("rel_abundance_genus.txt", header=TRUE, row.names=1)
species <- read.table("rel_abundance_species.txt", header=TRUE, row.names=1)
meta <- read.table("metadata.txt", header=TRUE, row.names=1, na.strings="NA")
all_consort <- read.table("all_consorts.txt", header=TRUE)
bray <- read.table("bray_curtis_matrix.txt", header=TRUE, row.names=1)
unifrac <- read.table("weighted_unifrac_matrix.txt", header=TRUE, row.names=1)
lefse_rep <- read.table("lefse_rep.txt", header=TRUE, row.names=1)
lefse_cycle <- read.table("lefse_cycle.txt", header=TRUE, row.names=1)
ph <- read.table("vaginal_ph.txt", header=TRUE)
relate <- as.matrix(read.table("relatedness.txt", header=TRUE, row.names=1))
history <- as.matrix(read.table("consort_history.txt", header=TRUE, row.names=1
))
```

Sequencing metrics:

```
read_count <- data.frame(read_count=(colSums(otu[1:52])))
# Total read count
sum(read_count$read_count)
```

```
## [1] 188665626
```

```
# Range read count per sample
range(read_count$read_count)
```

```
## [1] 1777373 7024839
```

```
# Mean read count per sample
mean(read_count$read_count)
```

```
## [1] 3628185
```

```
# Add read count to metadata dataframe
meta <- merge(meta, read_count, by="row.names")
rownames(meta) <- meta[,'Row.names']
meta <- subset(meta, select=-c(Row.names))
```

```
# Good's Coverage Estimator
# 1 – (n / N)], where n is the number of OTUs in a single read and N is the tot
al number of reads analyzed
n <- colSums(otu[1:52] == 1)
N <- colSums(otu[1:52])
coverage <- data.frame(coverage=1-(n/N))
# Add Good's Coverage Estimator to metadata dataframe
meta <- merge(meta, coverage, by="row.names")
rownames(meta) <- meta[,'Row.names']
meta <- subset(meta, select=-c(Row.names))
meta$coverage
```

```
##  [1] 0.9998651 0.9997914 0.9997550 0.9998283 0.9997871 0.9998451 0.9998425
##  [8] 0.9998377 0.9996994 0.9996186 0.9997203 0.9997668 0.9997645 0.9997434
## [15] 0.9997197 0.9998093 0.9997875 0.9996394 0.9998208 0.9998171 0.9996327
## [22] 0.9998421 0.9998164 0.9997938 0.9995696 0.9996584 0.9996595 0.9997580
## [29] 0.9997624 0.9997552 0.9996736 0.9996857 0.9996358 0.9997834 0.9997213
## [36] 0.9998960 0.9997389 0.9998260 0.9997192 0.9997722 0.9997255 0.9997753
## [43] 0.9997025 0.9998384 0.9998030 0.9997568 0.9998379 0.9996313 0.9997512
## [50] 0.9998059 0.9998378 0.9998069
```

OTU table normalization:

To control for differences in sequencing depth between samples, we normalized OTU table read counts using cumulative-sum scaling implemented in the R package, metagenomeSeq (version 1.4.2).

- Note: Starting with version 1.7.10, metagenomeSeq updated the default quantile estimate (.5) for low estimates. For the version or metagenomeSeq used in this manuscript (1.4.2), the quantile estimate was 0.3144699.

```
library(metagenomeSeq) #version 1.4.2
obj <- newMRexperiment(as.matrix(otu[1:52]))
p <- cumNormStat(obj)
# Use p <- 0.3144699 if using metagenomeSeq version 1.7.10 or later.
obj_sf <- cumNorm(obj, p=p)
otu_norm <- MRcounts(obj_sf, norm=T)
# Add back in taxonomy
tax <- otu[,"taxonomy",drop=FALSE]
otu_norm_tax <- merge(otu_norm, tax, by="row.names")
rownames(otu_norm_tax) <- otu_norm_tax$Row.names
otu_norm_tax <- otu_norm_tax[,-1]
```

RESULTS

Taxonomic relative abundance

Calculate data for TABLE S2A:

```
# Calculate phylum prevalence and % relative abundance (mean, SD, minimunm, and
 maximum)
phylum1 <- phylum
rownames(phylum1) <- gsub("k__|p__", "", rownames(phylum1))
rownames(phylum1) <- gsub(";", "|", rownames(phylum1))
rownames(phylum1) <- gsub("Other", "Unclassified", rownames(phylum1))
rownames(phylum1) <- gsub("Unassigned", "Unclassified", rownames(phylum1))
rownames(phylum1) <- gsub("Candidate_division_SR1", "Candidate division SR1", r
ownames(phylum1))
Table_S2A <- cbind(as.data.frame(rowSums(phylum1 != 0)),
                   round(as.data.frame(rowSums(phylum1 != 0))/ncol(phylum1)*100
, 2),
                   format(as.data.frame(rowMeans(phylum1)*100), digits=3, scien
tific=TRUE),
                   format(as.data.frame(apply(phylum1, 1, sd, na.rm=TRUE)*100),
 digits=3, scientific=TRUE),
                   format(as.data.frame(apply(phylum1, 1, min, na.rm=TRUE)*100)
, digits=3, scientific=TRUE),
                   format(as.data.frame(apply(phylum1, 1, max, na.rm = TRUE)*10
0), digits=3, scientific=TRUE))
colnames(Table_S2A) <- c("prev_n", "prev", "ab_mean", "ab_sd", "ab_min", "ab_ma
x")
Table_S2A
```

```
##                                 prev_n   prev  ab_mean    ab_sd   ab_min
## Unclassified|Unclassified           52 100.00 1.37e+00 1.62e+00 2.93e-02
## Archaea|Euryarchaeota               44  84.62 1.07e-01 4.19e-01 0.00e+00
## Archaea|Thaumarchaeota              41  78.85 2.32e-03 4.09e-03 0.00e+00
## Bacteria|Unclassified               34  65.38 1.56e-04 3.33e-04 0.00e+00
## Bacteria|Acidobacteria              51  98.08 1.79e-02 3.47e-02 0.00e+00
## Bacteria|Actinobacteria             52 100.00 9.66e+00 1.19e+01 2.52e-01
## Bacteria|Armatimonadetes            45  86.54 4.99e-03 1.25e-02 0.00e+00
## Bacteria|Bacteroidetes              52 100.00 1.09e+01 7.89e+00 9.05e-01
## Bacteria|Candidate division SR1     52 100.00 2.67e-01 7.55e-01 1.29e-04
## Bacteria|Chlorobi                    6  11.54 1.63e-04 6.90e-04 0.00e+00
## Bacteria|Chloroflexi                51  98.08 2.38e-02 5.32e-02 0.00e+00
## Bacteria|Cyanobacteria              52 100.00 4.06e-02 1.00e-01 1.66e-05
## Bacteria|Deinococcus-Thermus        40  76.92 4.60e-03 1.48e-02 0.00e+00
## Bacteria|Elusimicrobia              35  67.31 1.79e-02 9.52e-02 0.00e+00
## Bacteria|Fibrobacteres              14  26.92 4.55e-04 2.07e-03 0.00e+00
## Bacteria|Firmicutes                 52 100.00 3.26e+01 1.83e+01 4.43e+00
## Bacteria|Fusobacteria               52 100.00 2.86e+01 2.24e+01 1.31e-02
## Bacteria|Gemmatimonadetes           52 100.00 3.06e-02 5.53e-02 5.97e-05
## Bacteria|Hydrogenedentes            12  23.08 5.54e-04 2.21e-03 0.00e+00
## Bacteria|Lentisphaerae              52 100.00 5.05e-01 1.60e+00 4.99e-05
## Bacteria|Nitrospirae                25  48.08 2.82e-04 6.56e-04 0.00e+00
## Bacteria|Planctomycetes             49  94.23 7.31e-03 1.44e-02 0.00e+00
## Bacteria|Proteobacteria             52 100.00 1.27e+01 1.46e+01 4.50e-01
## Bacteria|SHA-109                    32  61.54 8.40e-03 3.72e-02 0.00e+00
## Bacteria|Saccharibacteria           40  76.92 1.79e-03 4.47e-03 0.00e+00
## Bacteria|Spirochaetae               52 100.00 6.84e-01 1.07e+00 5.22e-04
## Bacteria|Synergistetes               6  11.54 4.13e-03 2.97e-02 0.00e+00
## Bacteria|TM6                         2   3.85 3.35e-06 2.00e-05 0.00e+00
## Bacteria|Tenericutes                52 100.00 2.53e+00 6.91e+00 1.03e-02
## Bacteria|Verrucomicrobia            47  90.38 6.06e-03 1.35e-02 0.00e+00
## Bacteria|WCHB1-60                    8  15.38 1.32e-04 6.26e-04 0.00e+00
##                                   ab_max
## Unclassified|Unclassified       6.52e+00
## Archaea|Euryarchaeota           2.73e+00
## Archaea|Thaumarchaeota          1.61e-02
## Bacteria|Unclassified           1.78e-03
## Bacteria|Acidobacteria          1.62e-01
## Bacteria|Actinobacteria         5.93e+01
## Bacteria|Armatimonadetes        7.80e-02
## Bacteria|Bacteroidetes          4.00e+01
## Bacteria|Candidate division SR1 4.53e+00
## Bacteria|Chlorobi               4.02e-03
## Bacteria|Chloroflexi            2.69e-01
## Bacteria|Cyanobacteria          5.02e-01
## Bacteria|Deinococcus-Thermus    1.02e-01
```

```
## Bacteria|Elusimicrobia          6.80e-01
## Bacteria|Fibrobacteres          1.44e-02
## Bacteria|Firmicutes             8.08e+01
## Bacteria|Fusobacteria           7.18e+01
## Bacteria|Gemmatimonadetes       2.37e-01
## Bacteria|Hydrogenedentes        1.42e-02
## Bacteria|Lentisphaerae          7.72e+00
## Bacteria|Nitrospirae            3.25e-03
## Bacteria|Planctomycetes         6.37e-02
## Bacteria|Proteobacteria         6.90e+01
## Bacteria|SHA-109                2.68e-01
## Bacteria|Saccharibacteria       2.87e-02
## Bacteria|Spirochaetae           5.42e+00
## Bacteria|Synergistetes          2.14e-01
## Bacteria|TM6                    1.41e-04
## Bacteria|Tenericutes            4.76e+01
## Bacteria|Verrucomicrobia        7.16e-02
## Bacteria|WCHB1-60               4.13e-03
```

How many bacterial and archaeal phyla?

```
nrow(subset(Table_S2A, !(rownames(Table_S2A) %in% c("Unclassified|Unclassified"
, "Bacteria|Unclassified"))))
```

```
## [1] 29
```

How many bacterial phyla have a prevalence of 100%?

```
nrow(subset(Table_S2A, !(rownames(Table_S2A) %in% c("Unclassified|Unclassified"
, "Bacteria|Unclassified")) & prev==100))
```

```
## [1] 11
```

Which bacterial phyla have a relative abundance ≥1%?

```
mean_p <- data.frame(ab_mean=rowMeans(phylum1)*100)
subset(mean_p, ab_mean >= 1 & !(rownames(mean_p) %in% c("Unclassified|Unclassif
ied", "Bacteria|Unclassified")))
```

```
##                              ab_mean
## Bacteria|Actinobacteria  9.662413
## Bacteria|Bacteroidetes  10.852255
## Bacteria|Firmicutes     32.554020
## Bacteria|Fusobacteria   28.574172
## Bacteria|Proteobacteria 12.717570
## Bacteria|Tenericutes     2.531569
```

FIGURE 2A:

```
# Subset into Archaea, Bacteria, or unclassified
# Subset out Bacteria phyla that have <1% mean relative abundance
archaea <- colSums(subset(phylum1, rownames(phylum1) %in% grep("Archaea", rowna
mes(phylum1), value=TRUE)))
unclassified <- colSums(subset(phylum1, rownames(phylum1) %in% grep('Unclassifi
ed\\|', rownames(phylum1), value=TRUE)))
bacteria <- subset(phylum1, rownames(phylum1) %in% grep('Bacteria', rownames(ph
ylum1), value=TRUE))
low_bacteria <- colSums(subset(bacteria, (rowMeans(bacteria) < 0.01) & (rowname
s(bacteria) != "Bacteria|Unclassified")))
high_bacteria <- subset(bacteria, rowMeans(bacteria) >= 0.01)
unclass_bacteria <- subset(bacteria, rownames(bacteria) == "Bacteria|Unclassifi
ed")
phylum2 <- as.matrix(rbind(high_bacteria, low_bacteria, unclass_bacteria, archa
ea, unclassified) * 100)
rownames(phylum2) <- c(rownames(high_bacteria), "Low abundance Bacteria", "Uncl
assified Bacteria", "Archaea", "Unclassified kingdom")
# Order samples by reproductive state and ovarian cycle phase
meta$rep_state <- factor(meta$rep_state, levels = c("PPA", "C", "P","MC"))
meta$cycle_phase <- factor(meta$cycle_phase, levels = c("A", "S", "O","D"))
meta1 <- meta[order(meta$rep_state, meta$cycle_phase, meta$ppa_day, meta$cycle_
day, meta$preg_day, na.last=FALSE),]
Figure_2A <- t(t(phylum2)[match(rownames(meta1), rownames(t(phylum2))),])
phylum_order <- c("Bacteria|Firmicutes", "Bacteria|Fusobacteria", "Bacteria|Pro
teobacteria", "Bacteria|Bacteroidetes", "Bacteria|Actinobacteria", "Bacteria|Te
nericutes", "Low abundance Bacteria", "Unclassified Bacteria", "Archaea", "Uncl
assified kingdom")
Figure_2A <- Figure_2A[match(phylum_order, rownames(Figure_2A)),]
# Stacked bar plot
layout(matrix(c(1,2), 1, 2), widths=c(11, 2))
par(xpd=NA)
color_p <- c("#984EA3","#FF7F00","#FFFF33", "#4DAF4A", "#377EB8", "#A65628", "#
999999", "#FB9A99", "#E41A1C", "black")
barplot(Figure_2A, axes=FALSE, space = 0.3, axisnames = FALSE, col=color_p)
axis(2, at=c(0, 20, 40, 60, 80, 100), pos=-0.5, cex.axis=1.5, ylab="Relative ph
ylum abundance (%)")
mtext("Relative phylum abundance (%)", side=2, line=1.5, cex=1.5)
legend(x=68, y=100, legend=rev(rownames(Figure_2A)), fill=rev(color_p), cex=1)
text(x=-7, y=105, "A", cex=2.5)
rect(c(0.3,14.7,25.05,36.8,41.9,51.1,66.6),rep(-5,7),c(14.4,24.75,36.45,41.6,50
.8,66.3,67.7),rep(-0.5,7))
mtext(c("PPA","A","S","O","D","P"), 1, at=c(7.35,19.75,30.9,39.4,46.6,58.9), li
ne=0.05, cex=1.4)
mtext("MC", 1, at=67.15, line=-0.05, cex=0.75, las=2, padj=0.5)
mtext(c("a", "b", "c", "d", "d", "c", "b", "a"), 3, at=c(3.4, 7.35,20.25,24.25,
33.3,54.1,65.83,67.15), line=0, cex=1.2)
```

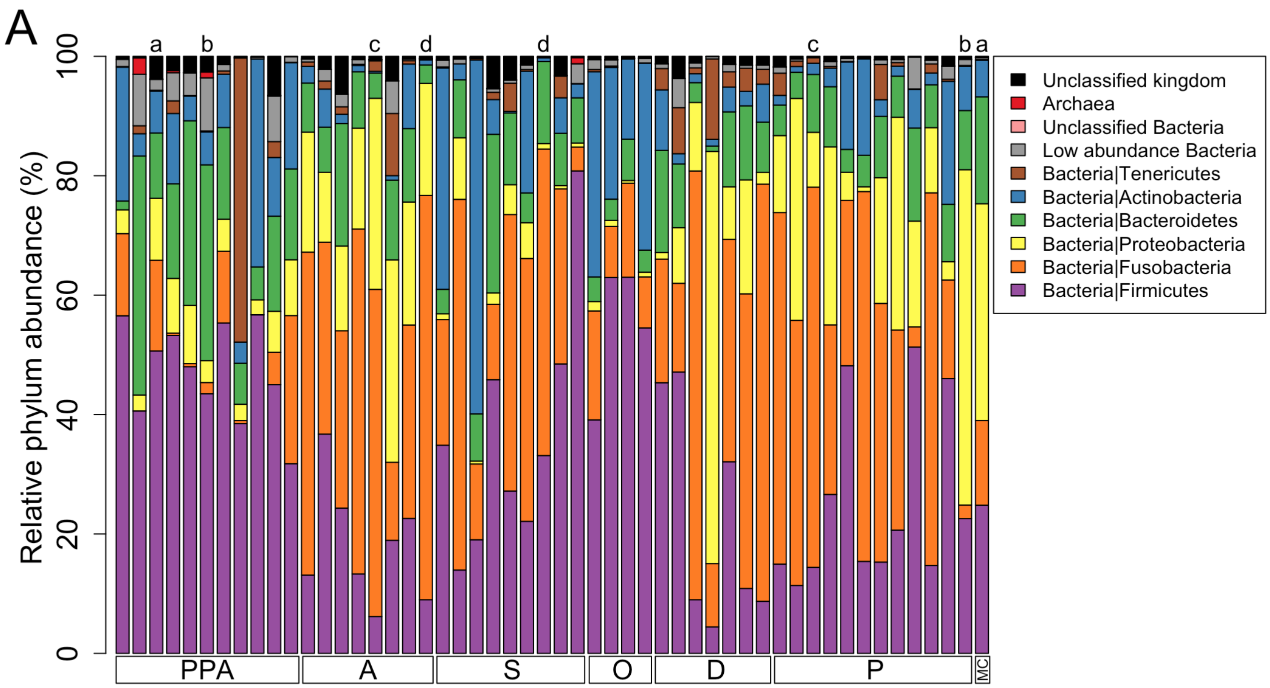

Calculate data for TABLE S2B:

```
# Keep only genera with a prevalence of 100% and classified to genus level
genus_prev <- as.data.frame(rowSums(genus != 0))
colnames(genus_prev) <- "prevalence_n"
genus_prev100 <- subset(genus_prev, prevalence_n==52 & !(rownames(genus_prev) %
in% grep("Other|g__uncultured|g__uncultured_bacterium$", rownames(genus_prev),
value=TRUE)))
genus100 <- subset(genus, rownames(genus) %in% rownames(genus_prev100))
rownames(genus100) <- gsub("^.*g__","", rownames(genus100))
rownames(genus100) <- gsub("_"," ", rownames(genus100))
# Calculate genus prevalence and % relative abundance (mean, SD, minimum, and m
aximum)
Table_S2B <- cbind(as.data.frame(rowSums(genus100 != 0)),
                   round(as.data.frame(rowSums(genus100 != 0))/ncol(genus100)*1
00, 2),
                   format(as.data.frame(rowMeans(genus100)*100), digits=3, scie
ntific=TRUE),
                   format(as.data.frame(apply(genus100, 1, sd, na.rm=TRUE)*100)
, digits=3, scientific=TRUE),
                   format(as.data.frame(apply(genus100, 1, min, na.rm=TRUE)*100
), digits=3, scientific=TRUE),
                   format(as.data.frame(apply(genus100, 1, max, na.rm = TRUE)*1
00), digits=3, scientific=TRUE))
colnames(Table_S2B) <- c("prev_n", "prev", "ab_mean", "ab_sd", "ab_min", "ab_ma
x")
Table_S2B
```

```
##                               prev_n prev  ab_mean    ab_sd   ab_min
## Arcanobacterium                   52  100 4.16e-01 1.26e+00 3.22e-05
## Mobiluncus                        52  100 6.74e-01 1.36e+00 7.74e-05
## Bifidobacterium                   52  100 4.52e+00 1.05e+01 3.41e-04
## Corynebacterium                   52  100 9.42e-01 2.67e+00 2.41e-04
## Corynebacterium 1                 52  100 1.64e+00 3.77e+00 1.41e-03
## Micrococcus                       52  100 3.78e-01 7.74e-01 3.80e-05
## Atopobium                         52  100 5.94e-01 1.64e+00 1.26e-04
## Porphyromonas                     52  100 6.03e+00 5.72e+00 3.66e-02
## Prevotella                        52  100 1.60e+00 3.05e+00 6.97e-05
## Prevotella 9                      52  100 5.27e-01 1.94e+00 6.66e-05
## Rikenellaceae RC9 gut group       52  100 1.07e+00 2.45e+00 1.04e-04
## Tumebacillus                      52  100 2.73e-02 5.52e-02 2.98e-05
## Bacillus                          52  100 9.08e-03 1.31e-02 2.47e-05
## Aerococcus                        52  100 3.05e+00 5.18e+00 3.31e-04
## Eremococcus                       52  100 9.22e-02 1.27e-01 8.95e-05
## Facklamia                         52  100 1.29e+00 2.42e+00 6.86e-04
## Atopostipes                       52  100 3.54e-01 5.97e-01 1.79e-04
## Trichococcus                      52  100 1.48e+00 4.39e+00 1.39e-04
## Streptococcus                     52  100 2.86e+00 1.11e+01 3.31e-04
## Christensenellaceae R-7 group     52  100 2.79e-01 4.05e-01 1.33e-03
## Anaerococcus                      52  100 2.82e+00 3.26e+00 8.13e-02
## Ezakiella                         52  100 3.04e+00 4.68e+00 1.13e-03
## Helcococcus                       52  100 1.39e+00 2.04e+00 1.37e-03
## Murdochiella                      52  100 7.82e-02 1.19e-01 2.56e-04
## Parvimonas                        52  100 1.45e+00 1.63e+00 8.88e-04
## Peptoniphilus                     52  100 2.88e+00 2.54e+00 1.93e-01
## Catonella                         52  100 1.67e+00 5.18e+00 2.10e-04
## Peptococcus                       52  100 1.27e-01 1.94e-01 4.65e-04
## Peptoclostridium                  52  100 8.05e-01 1.17e+00 5.57e-04
## Fastidiosipila                    52  100 1.93e+00 3.94e+00 1.20e-02
## Ruminococcaceae UCG-005           52  100 1.44e-01 4.34e-01 2.84e-05
## Ruminococcaceae UCG-014           52  100 3.79e-01 5.97e-01 2.39e-04
## Saccharofermentans                52  100 4.68e-02 6.20e-02 2.79e-04
## Dialister                         52  100 8.08e-01 1.28e+00 1.89e-03
## Negativicoccus                    52  100 3.64e-01 5.13e-01 3.95e-04
## Fusobacterium                     52  100 1.37e+01 1.32e+01 3.42e-03
## Sneathia                          52  100 9.11e+00 1.31e+01 6.30e-04
## Sphingomonas                      52  100 3.41e-02 1.17e-01 2.78e-05
## Campylobacter                     52  100 4.97e+00 6.93e+00 2.40e-01
## Haemophilus                       52  100 7.26e+00 1.29e+01 7.84e-04
## Treponema 2                       52  100 5.97e-01 1.05e+00 4.53e-04
## Acholeplasma                      52  100 9.55e-01 1.97e+00 4.20e-05
## Mycoplasma                        52  100 1.25e+00 6.60e+00 1.07e-04
##                                 ab_max
## Arcanobacterium               7.40e+00
```

```
## Mobiluncus                      5.92e+00
## Bifidobacterium                 5.31e+01
## Corynebacterium                 1.45e+01
## Corynebacterium 1               2.17e+01
## Micrococcus                     4.39e+00
## Atopobium                       1.03e+01
## Porphyromonas                   2.97e+01
## Prevotella                      1.24e+01
## Prevotella 9                    1.30e+01
## Rikenellaceae RC9 gut group     1.26e+01
## Tumebacillus                    3.41e-01
## Bacillus                        5.10e-02
## Aerococcus                      2.76e+01
## Eremococcus                     7.37e-01
## Facklamia                       1.18e+01
## Atopostipes                     3.76e+00
## Trichococcus                    1.98e+01
## Streptococcus                   6.88e+01
## Christensenellaceae R-7 group 1.95e+00
## Anaerococcus                    1.77e+01
## Ezakiella                       2.79e+01
## Helcococcus                     9.44e+00
## Murdochiella                    6.67e-01
## Parvimonas                      5.74e+00
## Peptoniphilus                   9.96e+00
## Catonella                       2.52e+01
## Peptococcus                     7.81e-01
## Peptoclostridium                5.13e+00
## Fastidiosipila                  2.58e+01
## Ruminococcaceae UCG-005         2.32e+00
## Ruminococcaceae UCG-014         2.48e+00
## Saccharofermentans              2.68e-01
## Dialister                       6.08e+00
## Negativicoccus                  2.69e+00
## Fusobacterium                   5.07e+01
## Sneathia                        6.69e+01
## Sphingomonas                    7.95e-01
## Campylobacter                   3.35e+01
## Haemophilus                     6.81e+01
## Treponema 2                     5.41e+00
## Acholeplasma                    1.01e+01
## Mycoplasma                      4.76e+01
```

How many bacterial genera have a prevalence of 100%?

```
nrow(Table_S2B)
```

```
## [1] 43
```

FIGURE 2B:

```
# Subset out genera that have >1% mean relative abundance
mean_g <- data.frame(ab_mean=rowMeans(genus)*100)
mean_g1 <- subset(mean_g, (ab_mean >= 1) & (rownames(mean_g) != "Unassigned;Oth
er;Other;Other;Other;Other"))
mean_g2 <- mean_g1[order(-mean_g1$ab_mean), , drop = FALSE]
genus_1 <- droplevels(subset(genus, rownames(genus) %in% rownames(mean_g2)))
genus_1 <- genus_1[match(rownames(mean_g2), rownames(genus_1)),]
# Combine bacterial genera with <1% relative abundance
genus_mean_low <- droplevels(subset(mean_g, ab_mean < 1))
bacteria_mean_low <- subset(genus_mean_low, (row(genus_mean_low) %in% grep("k__
Bacteria;", rownames(genus_mean_low))) & (rownames(genus_mean_low) != "k__Bacte
ria;Other;Other;Other;Other;Other"))
bacteria_low <- colSums(droplevels(subset(genus, rownames(genus) %in% rownames(
bacteria_mean_low))))
bacteria_unclass <- colSums(droplevels(subset(genus, rownames(genus)=="k__Bacte
ria;Other;Other;Other;Other;Other")))
# Combine archaea genera
archaea_mean <- subset(genus_mean_low, row(genus_mean_low) %in% grep("k__Archae
a;", rownames(genus_mean_low)))
archaea <- colSums(droplevels(subset(genus, rownames(genus) %in% rownames(archa
ea_mean))))
# Grep unclassified row
unclassified <- genus["Unassigned;Other;Other;Other;Other;Other",]
# Combine
genus1 <- rbind(genus_1, bacteria_low, bacteria_unclass, archaea, unclassified)
rownames(genus1) <- c(rownames(genus_1), "Low abundance Bacteria (mean < 1%)",
"Unclassified Bacteria", "Archaea",  "Unclassified kingdom")
genus2 <- as.matrix(genus1*100)
# Shorten row names
rownames(genus2) <- gsub("k__Bacteria;p__Fusobacteria;c__Fusobacteriia;o__Fusob
acteriales;f__Leptotrichiaceae;Other", "Unclassified Leptotrichiaceae", rowname
s(genus2))
rownames(genus2) <- sub(".*g__", "", rownames(genus2))
rownames(genus2) <- sub("_", " ", rownames(genus2))
rownames(genus2) <- sub("RC9_gut_group", "RC9 gut group", rownames(genus2))
# Order samples by reproductive state and cycle phase
Figure_2B <- t(t(genus2)[match(rownames(meta1), rownames(t(genus2))),])
# Heatmap
library(gplots)
library(RColorBrewer)
my_palette <- colorRampPalette(rev(brewer.pal(11,"Spectral")))(99)
my_breaks <- c(seq(-4,-2,length=20), seq(-1.99,1,length=60), seq(1.01,2,length=
20))
layout(matrix(c(2,1), 1, 2), widths=c(5, 5))
par(oma=c(0,1,0,5))
heatmap.2(log10(Figure_2B[c(1:22),]), Rowv=TRUE, Colv=FALSE, dendrogram="none",
```

```
 density.info="none",
          trace="none", col=my_palette, breaks=my_breaks, symkey=FALSE, colsep=
c(11, 19, 28, 32, 39, 51), sepcolor="white", sepwidth=c(0.3,0.02), labCol=NA, k
ey=TRUE, lhei=c(0.1,0.35,0.05), lmat=rbind(c(0,0,4), c(2,1,0), c(0,3,0)), lwid=
c(0.1, 4.5, 1.5), key.xlab=expression(paste(Log[10], " relative abundance",sep=
"")), key.title=NA, cexRow=1.75, key.par=list(cex.lab=1.5, cex.axis=1.5),
          add.expr = c({rect(c(0.55,11.8,19.8,28.8,32.9,39.9,51.8),rep(-0.75,7)
,c(11.5,19.5,28.5,32.6,39.6,51.5,52.8),rep(0.35,7),xpd=TRUE)},
              {mtext(c("PPA","A","S","O","D","P"), 1.5, at=c(6.03,15.65,24.15,3
0.75,36.3,45.7), line=-0.15, cex=1.3, padj = 0.8)},
              {mtext("MC", 1, at=52.3, line=0.3, cex=0.75, las=2, padj=0.5)},
              {mtext(c("a", "b", "c", "d", "d", "c", "b", "a"), 3, at=c(3, 6, 1
6, 19, 26, 42, 51, 52.2), line=0, cex =1.2)},
              {rect(c(0.55,11.865,19.85,28.88,32.88,39.88,51.88),0.5,c(11.44,19
.45,28.45,32.45,39.45,51.44,52.54),22.5, xpd=TRUE, col=NA)},
              {mtext("B", 2, cex=2.5, xpd=TRUE, las=1, at=c(24))}))
```

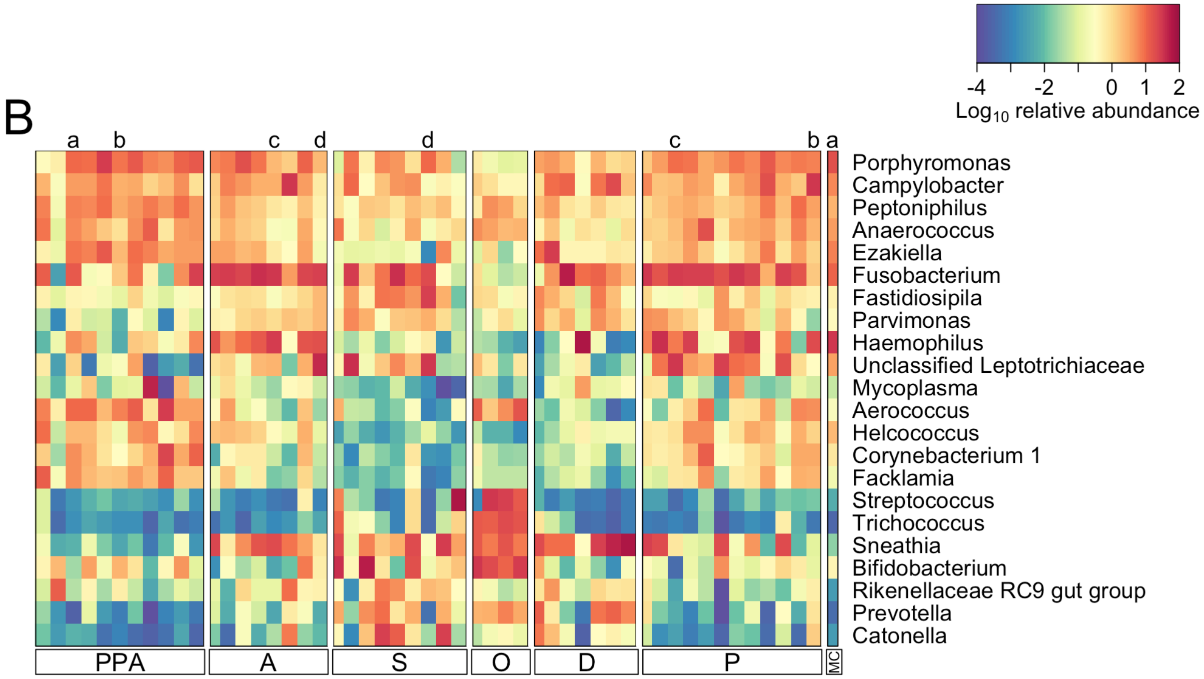

What is the prevalence and relative abundance of *Lactobacillus* spp.?

```
# Prevalence
lacto <- t(genus[grep(".*;g__Lactobacillus", rownames(genus)), ])
sum(lacto != 0)/nrow(lacto)*100
```

```
## [1] 84.61538
```

```
# Median relative abundance
median(lacto)*100
```

```
## [1] 0.0006315
```

```
# Relative abundance range
range(lacto)*100
```

```
## [1] 0.0000000 0.9324851
```

What is the relative abundance of the order Lactobacillales?

```
lacto1 <- order[grep(".*;o__Lactobacillales", rownames(order)), ]
# Mean relative abundance
rowMeans(lacto1)*100
```

```
## k__Bacteria;p__Firmicutes;c__Bacilli;o__Lactobacillales
##                                                 10.41283
```

```
# Relative abundance standard deviation
sd(lacto1)*100
```

```
## [1] 16.34909
```

What percentage of OTUs could not be classified beyond the kingdom level?

```
library(splitstackshape) #version 1.4.2
otu1 <- as.data.frame(cSplit(otu, "taxonomy", sep=';'))
rownames(otu1) <- rownames(otu)
unclass <- subset(otu1, !is.na(taxonomy_1) & is.na(taxonomy_2))
nrow(unclass)/nrow(otu)*100
```

```
## [1] 11.80084
```

What percentage of total reads were from unclassified OTUs?

```
sum(unclass[1:52])/sum(otu[1:52])*100
```

```
## [1] 1.522719
```

What percentage of unclassified OTUs appeare in more than one sample?

```
# Use OTU table with all OTUs (not just those that occur in more than one sampl
e)
otu_all_unclass <- subset(otu_all, rownames(otu_all) %in% rownames(unclass))
unclass_prev <- as.data.frame(rowSums(otu_all_unclass[1:52] > 0), drop=FALSE)
names(unclass_prev) <- "prevalence"
nrow(subset(unclass_prev, prevalence > 1))/nrow(unclass)*100
```

```
## [1] 100
```

How does the prevalence distribution of unclassified OTUs compare to the distribution of classified OTUs?

```
# Distribution of classified OTU prevalence
class_prev <- as.data.frame(rowSums(otu[1:52] > 0))
hist(class_prev[,1], breaks=16, ylab='Relative frequency of classified OTUs', x
lab='Prevalence of classified OTUs', main=NA, col="gray", cex.axis=1.5, cex.lab
=1.25, freq=FALSE, ylim=c(0,0.08))
```

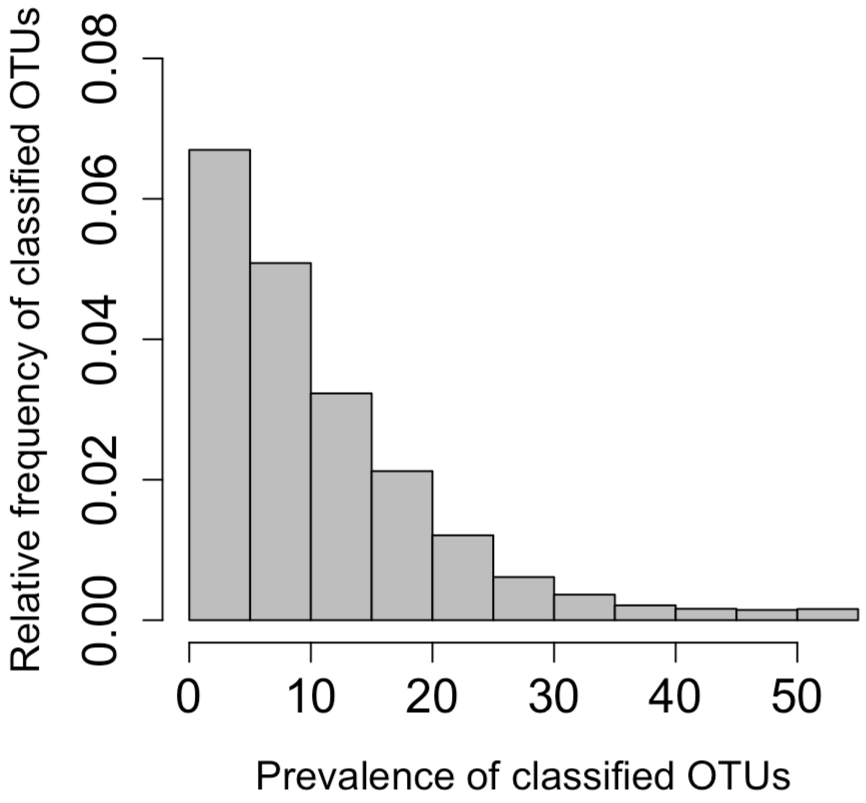


```
# Distribution of classified OTU prevalence
unclass_prev <- as.data.frame(rowSums(unclass[1:52] > 0))
hist(unclass_prev[,1], breaks=16, ylab='Relative frequency of unclassified OTUs
', xlab='Prevalence of unclassified OTUs', main=NA, col="gray", cex.axis=1.5, c
ex.lab=1.25, freq=FALSE, ylim=c(0,0.08))
```

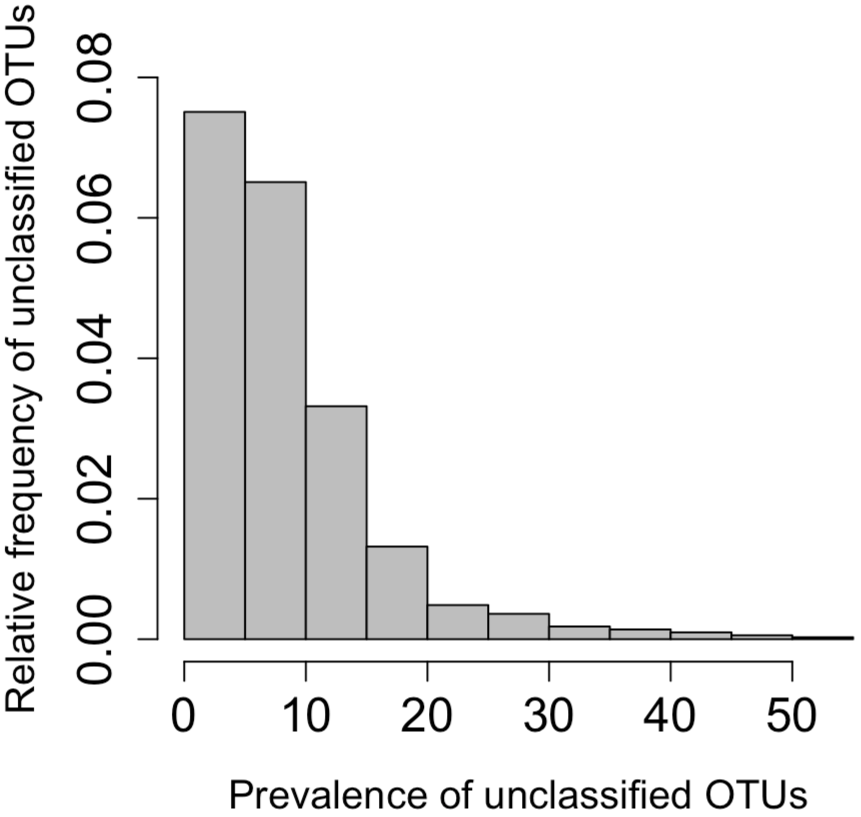

Alpha diversity

What host factors predict alpha diversity?

We constructed multivariate linear regression models for both OTU richness and Shannon's diversity index. The following factors were fixed effects: sequencing read count (to control for variation in sequencing depth between samples), age, reproductive state or ovarian cycle phase, dominance rank, presence or absence of rainfall in the 30 days prior to sample collection, the number of individuals in a female's social group at sample collection (i.e. social group size), and level of promiscuity, estimated using the average number of consortship partners per ovarian cycle.

```
# Calculate OTU richness and Shannon's diversity index
library(vegan) #version 2.4-0
library(plyr) #version 1.8.4
otu_matrix <- t(as.matrix(otu[1:52]))
richness <- data.frame(richness=specnumber(otu_matrix), rn=row.names(otu_matrix
))
shannon <- data.frame(diversity(otu_matrix, index="shannon", base=2), rn=row.na
mes(otu_matrix))
colnames(shannon) <- c("shannon", "rn")
meta1 <- join_all(list(data.frame(meta, rn=row.names(meta)), richness, shannon)
, by="rn", type='full')
rownames(meta1) <- meta1$rn
meta1$rn <- NULL
```

Should we include baboon ID as a random effect in the alpha diversity linear regression models?

Four baboons were sampled twice.

```r
library(cluster) #version 2.0.4
# Do samples from the same baboon have more similar alpha diversity?
# Dataframe of all possible sample dyads:
bid <- data.frame(sname=meta$baboon_id)
bid_gower <- daisy(bid, metric="gower")
sid <- data.frame(baboon_id=meta$baboon_id, row.names=rownames(meta))
all_dyads <- data.frame(t(combn(row.names(sid),2)), as.factor(as.numeric(bid_go
wer)))
names(all_dyads) <- c("sample_id1", "sample_id2", "same_baboon")
# Control alpha diversity for read count, reproductive state, and cycle phase.
meta1$"rep_cycle" <- ifelse(meta1$rep_state=="MC","MC",ifelse(meta1$rep_state==
"P","P",ifelse(meta1$rep_state=="PPA","PPA",ifelse(meta1$cycle_phase=="A","A",i
felse(meta1$cycle_phase=="S","S",ifelse(meta1$cycle_phase=="O","O","D"))))))
meta1$"richness_residuals" <- residuals(lm(richness ~ read_count + rep_cycle, d
ata=meta1))
meta1$"shannon_residuals" <- residuals(lm(shannon ~ read_count + rep_cycle, dat
a=meta1))
all_dyads1 <- merge(all_dyads, meta1[,c("richness_residuals", "shannon_residual
s")], by.x="sample_id1", by.y="row.names")
all_dyads2 <- merge(all_dyads1, meta1[,c("richness_residuals", "shannon_residua
ls")], by.x="sample_id2", by.y="row.names")
all_dyads2$"richness" <- abs(all_dyads2$richness_residuals.x-all_dyads2$richnes
s_residuals.y)
all_dyads2$"shannon" <- abs(all_dyads2$shannon_residuals.x-all_dyads2$shannon_r
esiduals.y)
# Because sample sizes are not equal, use Wilcoxon rank-sum tests (Mann–Whitney
 U tests)
# OTU Richness
wilcox.test(all_dyads2$"richness" ~ all_dyads2$same_baboon)
```

```
##
##  Wilcoxon rank sum test with continuity correction
##
## data:  all_dyads2$richness by all_dyads2$same_baboon
## W = 1725, p-value = 0.2297
## alternative hypothesis: true location shift is not equal to 0
```

```r
# Shannon's diversity index
wilcox.test(all_dyads2$"shannon" ~ all_dyads2$same_baboon)
```

```
##
##  Wilcoxon rank sum test with continuity correction
##
## data:  all_dyads2$shannon by all_dyads2$same_baboon
## W = 2876, p-value = 0.7621
## alternative hypothesis: true location shift is not equal to 0
```

Based on Wilcoxon rank sum tests (Mann–Whitney U tests), samples from the same baboon are no more similar to each other in terms of alpha diversity than samples from different baboons.

FIGURE S2

```
library(reshape) #version 0.8.5
library(ggplot2) #version 2.1.0
all_dyads_melt <- melt(all_dyads2[,c("sample_id1", "sample_id2","same_baboon","
richness","shannon")])
all_dyads_melt$variable <- gsub("richness", "OTU\nRichness", all_dyads_melt$var
iable)
all_dyads_melt$variable <- gsub("shannon", "Shannon's\nDiversity", all_dyads_me
lt$variable)
ggplot(data=all_dyads_melt, aes(x=variable, y=value)) +
  geom_boxplot(aes(fill=same_baboon), width=0.75, position=position_dodge(0.75)
) +
  facet_wrap( ~ variable, scales="free", ncol=5) +
  ylab("Absolute difference in alpha diversity (residuals)") + xlab("") +
  theme(plot.background=element_blank(), panel.grid.major=element_blank(), pane
l.grid.minor=element_blank(), panel.border=element_rect(fill=NA, colour="black"
, size=1), panel.background=element_blank(), axis.line=element_blank(), legend.
position="right", axis.title.x=element_text(color="black", size=16), axis.title
.y=element_text(color="black", size=16, hjust=0.25), axis.text.x=element_blank(
), axis.text.y=element_text(size=14, lineheight=0.9, color="black"), strip.text
.x=element_text(size=12, colour="black"), legend.text=element_text(size=14), le
gend.background=element_rect(fill="white", size=.5, linetype="solid", color="bl
ack"), legend.title=element_blank()) +
  scale_fill_manual(values=c("white","gray"), labels=c("Same Individual","Diffe
rent Individual"), name="")
```

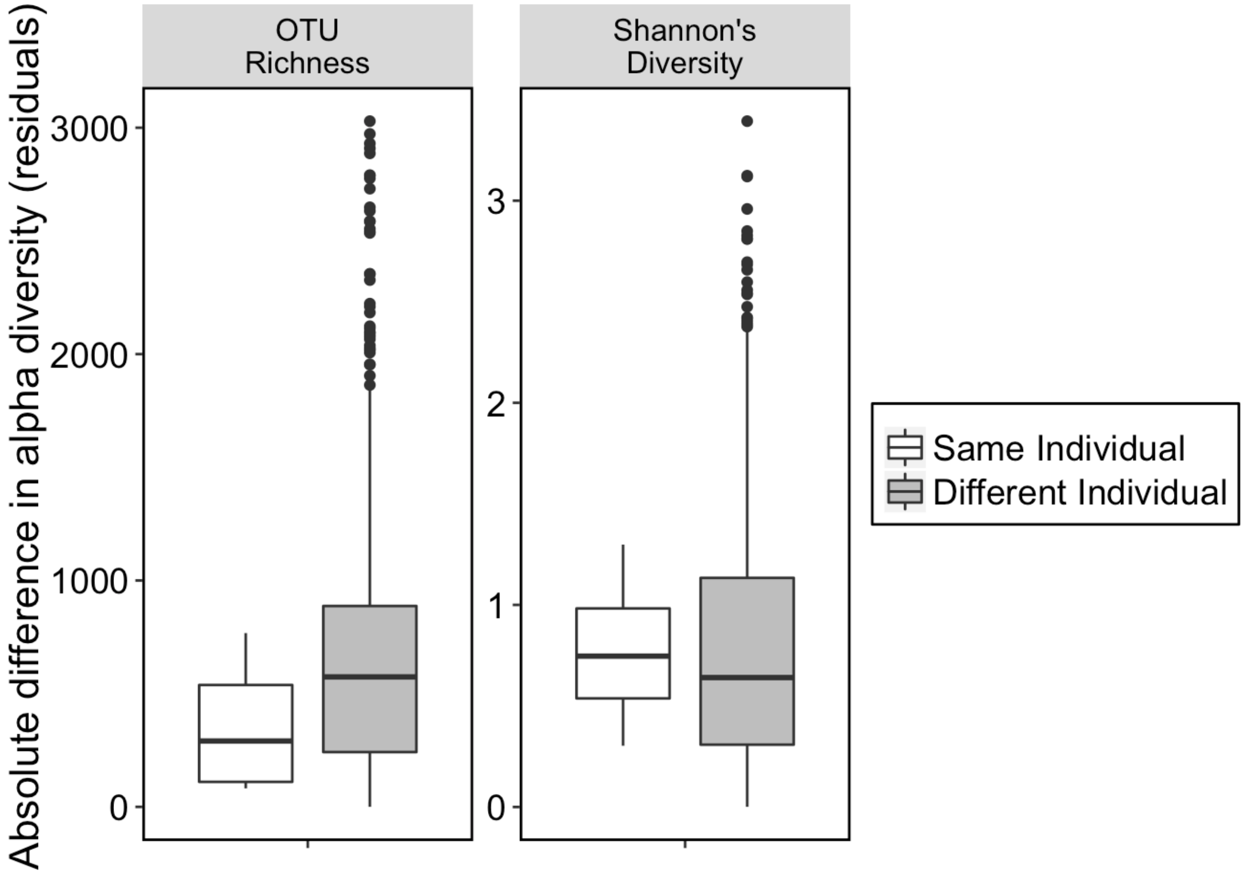

Calculate data for TABLE S3:

```
library(MASS) #version 7.3-45
# Remove the female who is miscarrying
meta_noMC <- droplevels(subset(meta1, rep_state != "MC"))
# Scale numerical factors
meta_noMC$age.scaled <- as.numeric(scale(meta_noMC$age, scale=TRUE))
meta_noMC$absolute_rank.scaled <- as.numeric(scale(meta_noMC$absolute_rank, sca
le=TRUE))
meta_noMC$read_count.scaled <- as.numeric(scale(meta_noMC$read_count, scale=TRU
E))
meta_noMC$social_grp_size.scaled <- as.numeric(scale(meta_noMC$social_grp_size,
 scale=TRUE))
meta_noMC$promiscuity.scaled <- as.numeric(scale(meta_noMC$promiscuity, scale=T
RUE))
# Linear regression for OTU richness
lm_full_R <- lm(richness ~ (read_count.scaled + age.scaled + rep_state + rainfa
ll + absolute_rank.scaled + promiscuity.scaled + social_grp_size.scaled), data=
meta_noMC)
stepAIC(lm_full_R, direction="backward", trace=0)
```

```
##
## Call:
## lm(formula = richness ~ read_count.scaled + rainfall, data = meta_noMC)
##
## Coefficients:
##       (Intercept)  read_count.scaled          rainfallR
##            2932.2              563.1             -467.6
```

```
# Final model for OTU richness
summary(lm(richness ~ (read_count.scaled + rainfall), data=meta_noMC))
```

```
##
## Call:
## lm(formula = richness ~ (read_count.scaled + rainfall), data = meta_noMC)
##
## Residuals:
##      Min       1Q   Median       3Q      Max
## -1223.23  -350.77   -91.13   253.08  1727.37
##
## Coefficients:
##                   Estimate Std. Error t value Pr(>|t|)
## (Intercept)         2932.2      128.8  22.766  < 2e-16 ***
## read_count.scaled    563.1       89.5   6.292 8.98e-08 ***
## rainfallR           -467.6      177.6  -2.633   0.0113 *
## ---
## Signif. codes:  0 '***' 0.001 '**' 0.01 '*' 0.05 '.' 0.1 ' ' 1
##
## Residual standard error: 628.8 on 48 degrees of freedom
## Multiple R-squared:  0.4744, Adjusted R-squared:  0.4525
## F-statistic: 21.66 on 2 and 48 DF,  p-value: 1.977e-07
```

```
# Linear regression for Shannon's diversity index
lm_full_H <- lm(shannon ~ (read_count.scaled + age.scaled + rep_state + rainfal
l + absolute_rank.scaled + promiscuity.scaled + social_grp_size.scaled), data=m
eta_noMC)
stepAIC(lm_full_H, direction="backward", trace=0)
```

```
##
## Call:
## lm(formula = shannon ~ read_count.scaled + rep_state + rainfall +
##     promiscuity.scaled + social_grp_size.scaled, data = meta_noMC)
##
## Coefficients:
##            (Intercept)        read_count.scaled              rep_stateC
##                 5.5979                   0.4157                 -1.7263
##             rep_stateP                rainfallR      promiscuity.scaled
##                -0.9182                  -0.3319                 -0.1525
## social_grp_size.scaled
##                -0.3014
```

```
summary(lm(shannon ~ (read_count.scaled + age.scaled + rep_state + rainfall + a
bsolute_rank.scaled + promiscuity.scaled + social_grp_size.scaled), data=meta_n
oMC))
```

```
##
## Call:
## lm(formula = shannon ~ (read_count.scaled + age.scaled + rep_state +
##     rainfall + absolute_rank.scaled + promiscuity.scaled + social_grp_size.s
caled),
##     data = meta_noMC)
##
## Residuals:
##     Min      1Q  Median      3Q     Max
## -1.4969 -0.4512 -0.1016  0.4630  1.4915
##
## Coefficients:
##                        Estimate Std. Error t value Pr(>|t|)
## (Intercept)             5.59870    0.25755  21.739  < 2e-16 ***
## read_count.scaled       0.41445    0.12874   3.219  0.00248 **
## age.scaled             -0.02778    0.11521  -0.241  0.81062
## rep_stateC             -1.71574    0.31412  -5.462 2.35e-06 ***
## rep_stateP             -0.94702    0.31904  -2.968  0.00493 **
## rainfallR              -0.33144    0.21968  -1.509  0.13885
## absolute_rank.scaled   -0.05910    0.13095  -0.451  0.65406
## promiscuity.scaled     -0.16246    0.11617  -1.399  0.16929
## social_grp_size.scaled -0.26448    0.13173  -2.008  0.05113 .
## ---
## Signif. codes:  0 '***' 0.001 '**' 0.01 '*' 0.05 '.' 0.1 ' ' 1
##
## Residual standard error: 0.7478 on 42 degrees of freedom
## Multiple R-squared:  0.5499, Adjusted R-squared:  0.4642
## F-statistic: 6.414 on 8 and 42 DF,  p-value: 1.976e-05
```

```
# Remove age, rainfall, absolute rank, and promiscuity because Pr(>|t|) > 0.05
# What happens to social group size, which is borderline significant?
summary(lm(shannon ~ (read_count.scaled + rep_state + social_grp_size.scaled),
data=meta_noMC))
```

```
##
## Call:
## lm(formula = shannon ~ (read_count.scaled + rep_state + social_grp_size.scal
ed),
##     data = meta_noMC)
##
## Residuals:
##     Min      1Q  Median      3Q     Max
## -1.3437 -0.5043 -0.1276  0.5277  1.6622
##
## Coefficients:
##                       Estimate Std. Error t value Pr(>|t|)
## (Intercept)             5.4309     0.2366  22.954  < 2e-16 ***
## read_count.scaled       0.3761     0.1247   3.016  0.00417 **
## rep_stateC             -1.7188     0.2969  -5.789 5.99e-07 ***
## rep_stateP             -0.9724     0.3114  -3.122  0.00310 **
## social_grp_size.scaled  -0.3252     0.1094  -2.972  0.00470 **
## ---
## Signif. codes:  0 '***' 0.001 '**' 0.01 '*' 0.05 '.' 0.1 ' ' 1
##
## Residual standard error: 0.7448 on 46 degrees of freedom
## Multiple R-squared:  0.511,  Adjusted R-squared:  0.4684
## F-statistic: 12.02 on 4 and 46 DF,  p-value: 9.128e-07
```

Calculate data for TABLE S4:

```
meta_C <- droplevels(subset(meta_noMC, rep_state == "C"))
# Linear regression for OTU richness
lm_full_R_C <- lm(richness ~ (read_count.scaled + age.scaled + cycle_phase + ra
infall + absolute_rank.scaled + promiscuity.scaled + social_grp_size.scaled), d
ata=meta_C)
stepAIC(lm_full_R_C, direction="backward", trace=0)
```

```
##
## Call:
## lm(formula = richness ~ read_count.scaled + cycle_phase + rainfall,
##     data = meta_C)
##
## Coefficients:
##       (Intercept)  read_count.scaled       cycle_phaseS
##           3288.52             721.47            -731.57
##      cycle_phaseO       cycle_phaseD          rainfallR
##             31.95            -863.91            -325.83
```

```
summary(lm(richness ~ (read_count.scaled + cycle_phase + rainfall), data=meta_C
))
```

```
##
## Call:
## lm(formula = richness ~ (read_count.scaled + cycle_phase + rainfall),
##     data = meta_C)
##
## Residuals:
##      Min       1Q   Median       3Q      Max
## -1236.06  -288.07   -38.04   217.52  1842.66
##
## Coefficients:
##                   Estimate Std. Error t value Pr(>|t|)
## (Intercept)        3288.52     273.05  12.044 3.71e-11 ***
## read_count.scaled   721.47     135.92   5.308 2.51e-05 ***
## cycle_phaseS       -731.57     363.92  -2.010   0.0568 .
## cycle_phaseO         31.95     399.16   0.080   0.9369
## cycle_phaseD       -863.91     364.71  -2.369   0.0270 *
## rainfallR          -325.83     254.92  -1.278   0.2145
## ---
## Signif. codes:  0 '***' 0.001 '**' 0.01 '*' 0.05 '.' 0.1 ' ' 1
##
## Residual standard error: 622.2 on 22 degrees of freedom
## Multiple R-squared:  0.6182, Adjusted R-squared:  0.5315
## F-statistic: 7.125 on 5 and 22 DF,  p-value: 0.0004245
```

```
# Remove rainfall from final model because Pr(>|t|) > 0.05
# Final model for OTU richness
summary(lm(richness ~ (read_count.scaled + cycle_phase), data=meta_C))
```

```
##
## Call:
## lm(formula = richness ~ (read_count.scaled + cycle_phase), data = meta_C)
##
## Residuals:
##      Min       1Q   Median       3Q      Max
## -1049.02  -235.07    24.79   160.59  2047.47
##
## Coefficients:
##                   Estimate Std. Error t value Pr(>|t|)
## (Intercept)         3093.7      229.6  13.472 2.13e-12 ***
## read_count.scaled    743.4      136.7   5.439 1.58e-05 ***
## cycle_phaseS        -777.6      367.1  -2.118   0.0452 *
## cycle_phaseO         140.5      395.4   0.356   0.7255
## cycle_phaseD        -920.0      367.0  -2.507   0.0197 *
## ---
## Signif. codes:  0 '***' 0.001 '**' 0.01 '*' 0.05 '.' 0.1 ' ' 1
##
## Residual standard error: 630.7 on 23 degrees of freedom
## Multiple R-squared:  0.5899, Adjusted R-squared:  0.5186
## F-statistic:  8.27 on 4 and 23 DF,  p-value: 0.0002752
```

```
# Linear regression for Shannon's diversity index
lm_full_H_C <- lm(shannon ~ (read_count.scaled + age.scaled + cycle_phase + rai
nfall + absolute_rank.scaled + promiscuity.scaled + social_grp_size.scaled), da
ta=meta_C)
stepAIC(lm_full_H_C, direction="backward", trace=0)
```

```
##
## Call:
## lm(formula = shannon ~ read_count.scaled + cycle_phase + social_grp_size.sca
led,
##     data = meta_C)
##
## Coefficients:
##            (Intercept)       read_count.scaled            cycle_phaseS
##                 4.3547                  0.5835                 -1.0160
##           cycle_phaseO            cycle_phaseD  social_grp_size.scaled
##                -0.7118                 -1.2716                 -0.2943
```

```
# Final model for Shannon's diversity index
summary(lm(shannon ~ (read_count.scaled + cycle_phase + social_grp_size.scaled)
, data=meta_C))
```

```
##
## Call:
## lm(formula = shannon ~ (read_count.scaled + cycle_phase + social_grp_size.sc
aled),
##     data = meta_C)
##
## Residuals:
##     Min      1Q  Median      3Q     Max
## -1.2726 -0.2955  0.0912  0.2415  0.7878
##
## Coefficients:
##                         Estimate Std. Error t value Pr(>|t|)
## (Intercept)               4.3547     0.1866  23.333  < 2e-16 ***
## read_count.scaled         0.5835     0.1116   5.230 3.03e-05 ***
## cycle_phaseS             -1.0160     0.3003  -3.383 0.002678 **
## cycle_phaseO             -0.7118     0.3219  -2.212 0.037691 *
## cycle_phaseD             -1.2716     0.2982  -4.264 0.000317 ***
## social_grp_size.scaled  -0.2943     0.1049  -2.806 0.010294 *
## ---
## Signif. codes:  0 '***' 0.001 '**' 0.01 '*' 0.05 '.' 0.1 ' ' 1
##
## Residual standard error: 0.5116 on 22 degrees of freedom
## Multiple R-squared:  0.6255, Adjusted R-squared:  0.5403
## F-statistic: 7.348 on 5 and 22 DF,  p-value: 0.0003492
```

FIGURE 3:

```
library(graphics) #version 3.2.2
# Panel A
meta_noMC$rep_state <- factor(meta_noMC$rep_state, c("C","P","PPA"))
meta_C$cycle_phase <- factor(meta_C$cycle_phase, c("A","S","O","D"))
bxpl_R <- boxplot(meta_noMC$shannon ~ meta_noMC$rep_state)
```

```
bxpl_C <- boxplot(meta_C$shannon ~ meta_C$cycle_phase)
```

```
bxp(bxpl_R, ylab="Shannon's Diversity Index", ylim=c(2,8), xlim=c(0.5,8.5), box
wex=0.5, cex.axis=1.25, cex.lab=1.5, boxfill= c("darkturquoise", "darkorange",
"purple"), at=c(1,2,3))
bxp(bxpl_C, ylab="", ylim=c(2,8), xlim=c(0.5,8.5), boxwex=0.5, cex.axis=1.25, c
ex.lab=1.5, boxfill= c("red", "gold3", "#33A02C", "blue"), at=c(5,6,7,8), add=T
RUE)
abline(v=4, lty=2, lwd=2)
segments(1,7.9,2,7.9, col="black",lwd=2.5) #C vs. P
text(x=1.5,y=8.05,labels="*",cex=2) #C vs. P
segments(1,7.45,3,7.45, col="black",lwd=2.5) #C vs. PPA
text(x=2,y=7.6,labels="***",cex=2) #C vs. PPA
segments(2,7,3,7, col="black",lwd=2.5) #P vs. PPA
text(x=2.5,y=7.15,labels="**",cex=2) #P vs. PPA
segments(5,5.4,6,5.4, col="black",lwd=2.5) #A vs. S
text(x=5.5,y=5.55,labels="**",cex=2) #A vs. S
segments(5,5.85,7,5.85, col="black",lwd=2.5) #A vs. O
text(x=6,y=6,labels="*",cex=2) #A vs. O
segments(5,6.3,8,6.3, col="black",lwd=2.5) #A vs. D
text(x=6.5,y=6.45,labels="***",cex=2) #A vs. D
mtext("A", side=3, at=c(-1), line=0, cex=2, font=2)
```

A

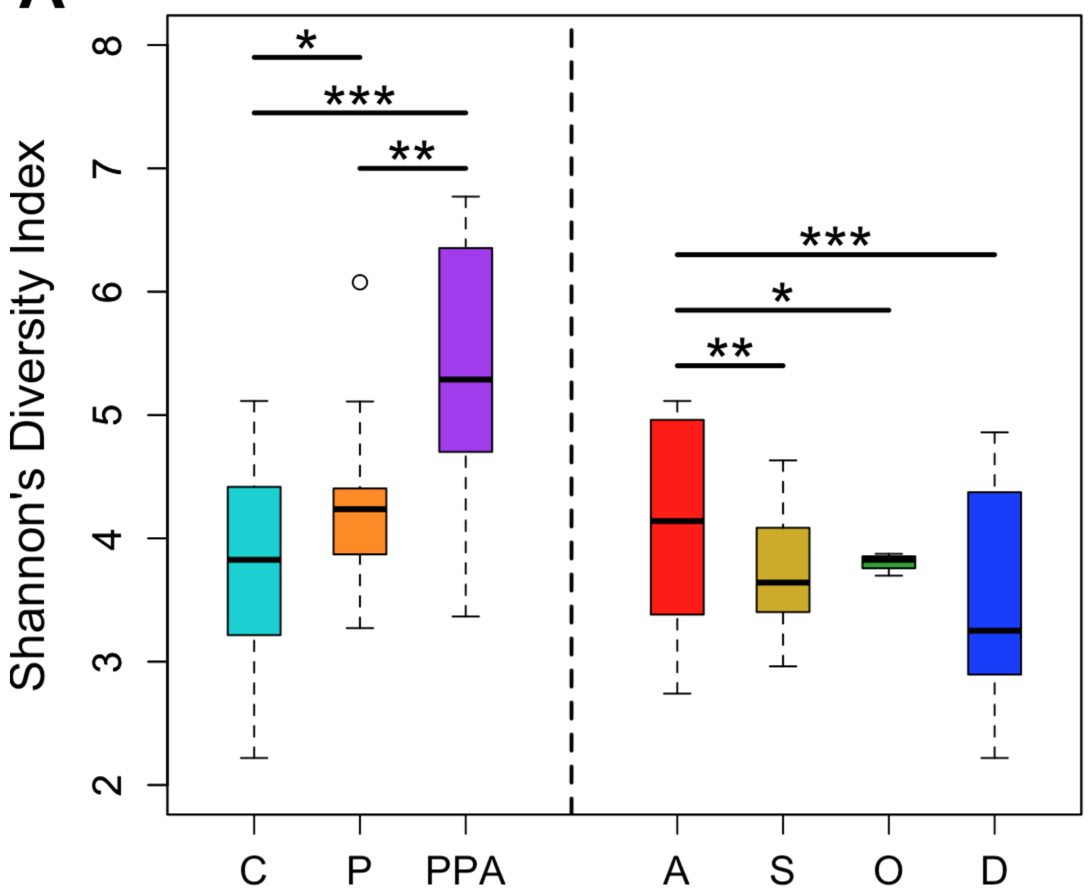


```
# Panel B
plot(meta_C$richness ~ meta_C$cycle_phase, pch=20, xlab=NA, ylab="OTU Richness"
, ylim=c(0,7000), boxwex=0.5, cex.axis=1.25, cex.lab=1.5, col= c("red", "gold3"
, "#33A02C", "blue"))
segments(1,6850,4,6850, col="black",lwd=2.5) #A vs. D
text(x=2.5,y=7000,labels="*",cex=2) #A vs. D
segments(3,5750,4,5750, col="black",lwd=2.5) #O vs. D
text(x=3.5,y=5900,labels="*",cex=2) #O vs. D
segments(1,6514,2,6514, col="black",lwd=2.5) #A vs. S
text(x=1.5,y=6664,labels="*",cex=2) #A vs. S
segments(2,6086,3,6086, col="black",lwd=2.5) #S vs. O
text(x=2.5,y=6236,labels="*",cex=2) #S vs. O
mtext("B", side=3, at=c(-0.25), line=0, cex=2, font=2)
```

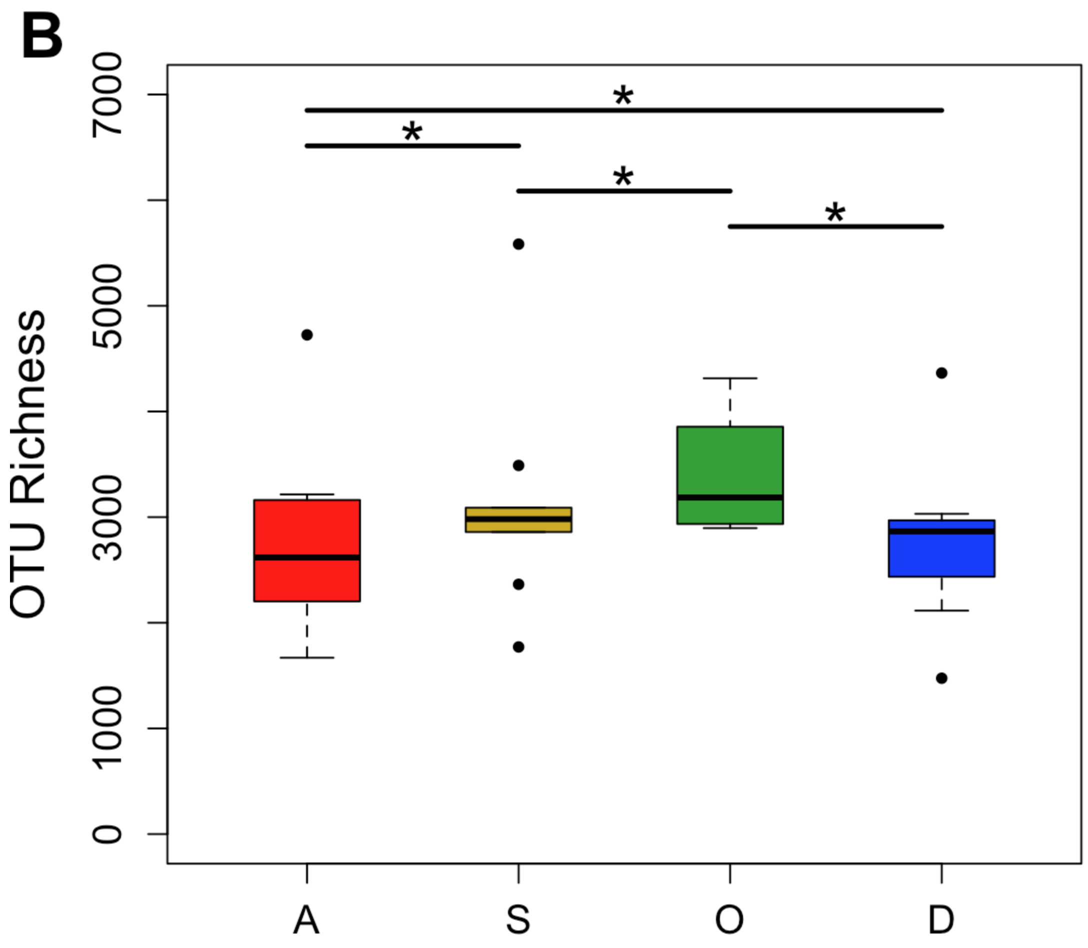


```
# Panel C
plot(meta_noMC$shannon ~ meta_noMC$social_grp_size, pch=20, xlab="Social Group
Size", ylab="Shannon's Diversity Index", cex.axis=1.25, cex.lab=1.5, xlim=c(20,
120), ylim=c(2,7))
abline(lm(meta_noMC$shannon ~ meta_noMC$social_grp_size), col="black")
mtext("C", side=3, at=c(0), line=0, cex=2, font=2)
```

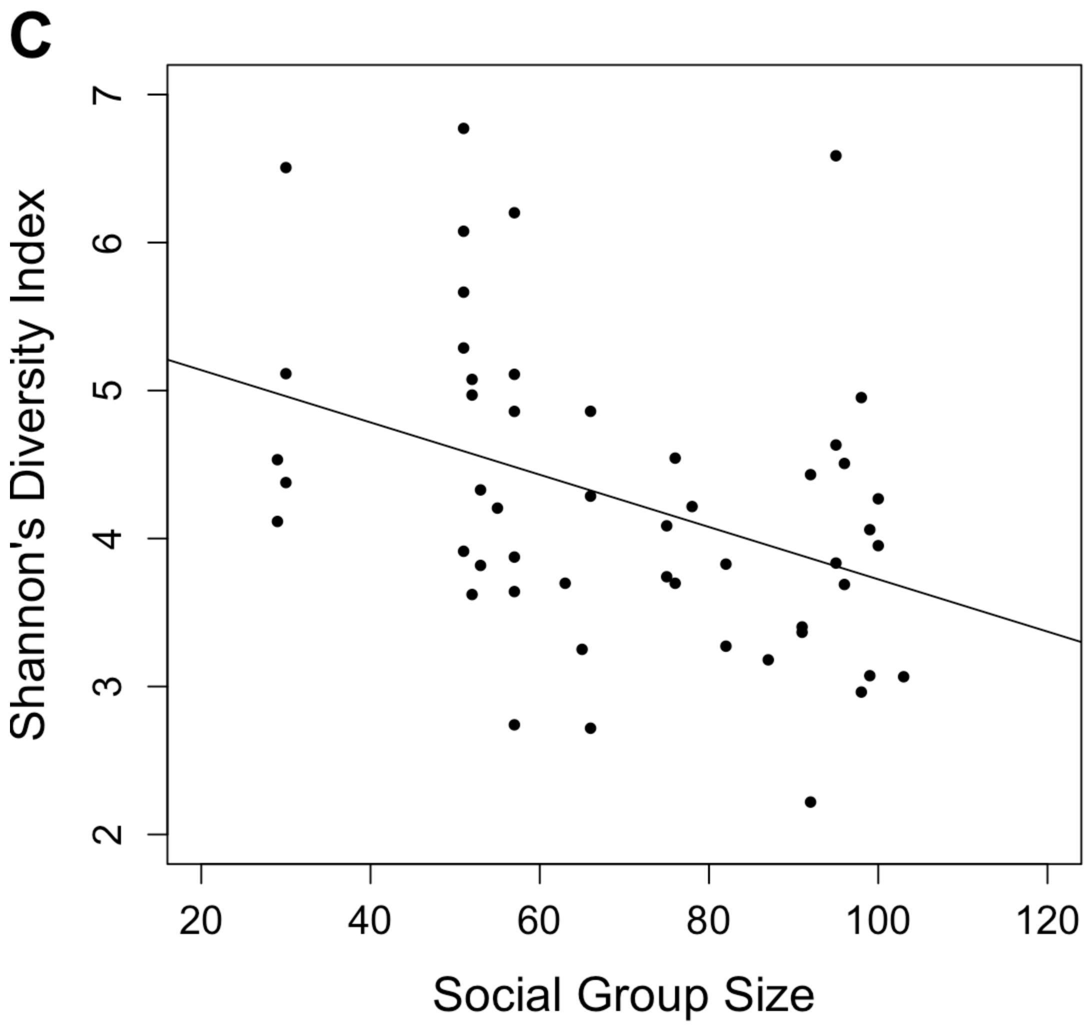


```
# Panel D
plot(meta_noMC$richness ~ meta_noMC$rainfall, pch=20, xlab="Rainfall in Past 30
 Days", ylab="OTU Richness", names=c("No Rainfall", "Rainfall"), ylim=c(0,7000)
, boxwex=0.5, cex.axis=1.25, cex.lab=1.5, col=c("burlywood4", "darkgreen"))
segments(1,6250,2,6250, col="black",lwd=2.5)
text(x=1.5,y=6500,labels="*",cex=2)
mtext("D", side=3, at=c(0.1), line=0, cex=2, font=2)
```

D

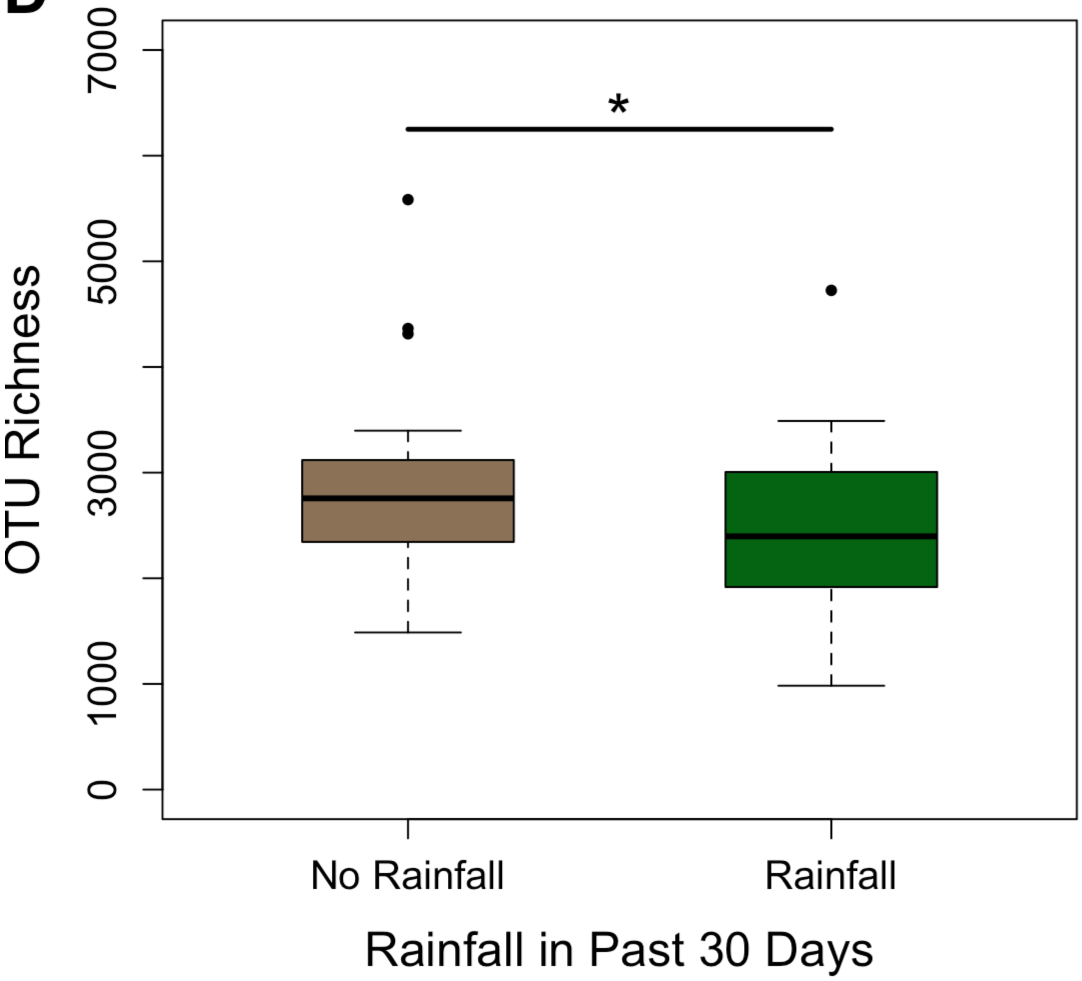

FIGURE S4:

```
meta_P <- droplevels(subset(meta_noMC, rep_state == "P"))
# Control for read count
plot(meta_P$preg_day, lm(shannon ~ read_count, data=meta_P)$residuals, xlab="Da
ys into Pregnancy", ylab="Shannon's Diversity Index (residuals)", pch=19, xlim=
c(0,100), cex.lab=1.5, cex.axis=1.5)
abline(lm(lm(shannon ~ read_count, data=meta_P)$residuals ~ preg_day, data=meta
_P), col="black")
```

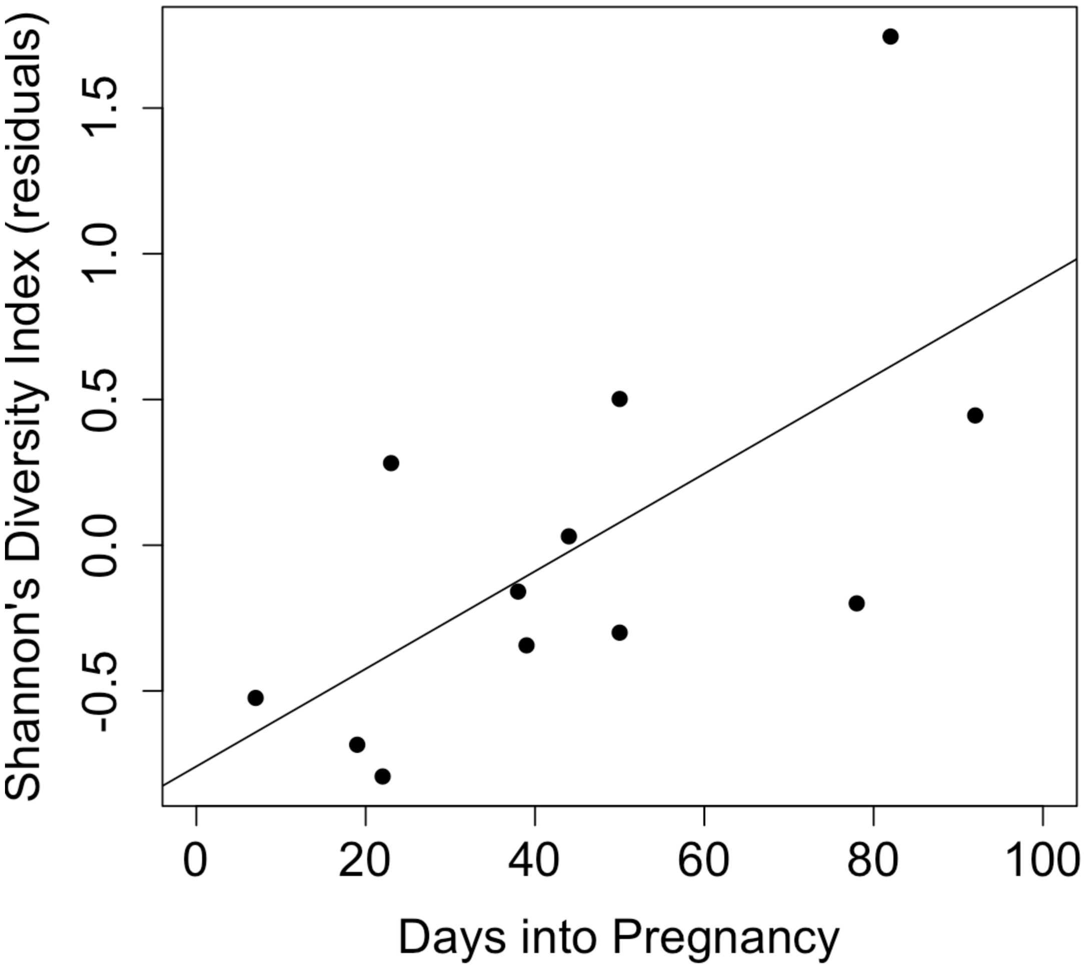

Do females in larger social groups experience more consortships and consortship partners per ovarian cycle than females in smaller groups?

We used behavioral data on all mature females present within the Amboseli Baboon Research Project study population between 2007 and 2010. For each ovarian cycle of each female (i.e. beginning of the swelling phase to the end of the deturgescence phase), we calculated the number of observed consortships and the number of distinct consortship partners.

```
# The number of observations can depend on the number of observers concurrently
 watching a group and the number of times a group was visited. Thus, we control
led for differences in observation intensity by using a proxy of a group's true
 observer effort: the number of focal animal samples that occurred in the group
 during a female's ovarian cycle divided by the average group size during the s
ame time period.
library(lme4) #version 1.1-12
all_consort$"obs_effort" <- all_consort$focal_samples/all_consort$avg_grp_size
# Scale all factors
all_consort$"obs_effort.scaled" <- scale(all_consort$obs_effort)
all_consort$"avg_grp_size.scaled" <- scale(all_consort$avg_grp_size)
all_consort$"consorter_count.scaled" <- scale(all_consort$consorter_count)
all_consort$"consort_count.scaled" <- scale(all_consort$consort_count)
# Relationship between number of consortships and social group size
summary(glmer(consort_count ~ (avg_grp_size.scaled + obs_effort.scaled) + (1|ba
boon_id), family="poisson", data=all_consort))
```

```
## Generalized linear mixed model fit by maximum likelihood (Laplace
##   Approximation) [glmerMod]
##  Family: poisson  ( log )
## Formula: consort_count ~ (avg_grp_size.scaled + obs_effort.scaled) + (1 |
##     baboon_id)
##    Data: all_consort
##
##      AIC      BIC   logLik deviance df.resid
##   4335.1   4354.4  -2163.6   4327.1      909
##
## Scaled residuals:
##     Min      1Q  Median      3Q     Max
## -3.0028 -0.9788 -0.2694  0.7178  7.2603
##
## Random effects:
##  Groups    Name        Variance Std.Dev.
##  baboon_id (Intercept) 0.4489   0.67
## Number of obs: 913, groups:  baboon_id, 117
##
## Fixed effects:
##                     Estimate Std. Error z value Pr(>|z|)
## (Intercept)          0.94619    0.06850   13.81   <2e-16 ***
## avg_grp_size.scaled  0.51308    0.05046   10.17   <2e-16 ***
## obs_effort.scaled    0.31730    0.02170   14.62   <2e-16 ***
## ---
## Signif. codes:  0 '***' 0.001 '**' 0.01 '*' 0.05 '.' 0.1 ' ' 1
##
## Correlation of Fixed Effects:
##             (Intr) avg__.
## avg_grp_sz. -0.122
## obs_ffrt.sc -0.051  0.330
```

```
# Relationship between number of unique consortship partners and social group s
ize
summary(glmer(consorter_count ~ (avg_grp_size.scaled + obs_effort.scaled) + (1|
baboon_id), family="poisson", data=all_consort))
```

```
## Generalized linear mixed model fit by maximum likelihood (Laplace
##   Approximation) [glmerMod]
##  Family: poisson  ( log )
## Formula: consorter_count ~ (avg_grp_size.scaled + obs_effort.scaled) +
##     (1 | baboon_id)
##    Data: all_consort
##
##      AIC      BIC   logLik deviance df.resid
##   2844.9   2864.2  -1418.5   2836.9      909
##
## Scaled residuals:
##     Min      1Q  Median      3Q     Max
## -1.6158 -0.7372 -0.1344  0.5503  3.8244
##
## Random effects:
##  Groups    Name        Variance Std.Dev.
##  baboon_id (Intercept) 0.1688   0.4109
## Number of obs: 913, groups:  baboon_id, 117
##
## Fixed effects:
##                     Estimate Std. Error z value Pr(>|z|)
## (Intercept)          0.42246    0.04981   8.482  < 2e-16 ***
## avg_grp_size.scaled  0.27899    0.05105   5.465 4.62e-08 ***
## obs_effort.scaled    0.16616    0.03474   4.783 1.72e-06 ***
## ---
## Signif. codes:  0 '***' 0.001 '**' 0.01 '*' 0.05 '.' 0.1 ' ' 1
##
## Correlation of Fixed Effects:
##             (Intr) avg__.
## avg_grp_sz. -0.122
## obs_ffrt.sc -0.060  0.499
```

FIGURE S5

```
par(mfrow=c(1,2))
# Control for observer effort and baboon_id
glmer_consort_residuals <- residuals(glmer(consort_count ~ (obs_effort.scaled)
+ (1|baboon_id), family="poisson", data=all_consort))
glmer_consorter_residuals <- residuals(glmer(consorter_count ~ (obs_effort.scal
ed) + (1|baboon_id), family="poisson", data=all_consort))
# Panel A
plot(glmer_consort_residuals ~ all_consort$avg_grp_size, xlab="Social group siz
e", ylab="No. of consortships (residuals)", pch=19, cex.lab=1.25, cex.axis=1.25
, xlim=c(20,120))
abline(lm(glmer_consort_residuals ~ all_consort$avg_grp_size),col="black")
mtext("A", side=3, at=c(-2), line=0.5, cex=2, font=2)
# Panel B
plot(glmer_consorter_residuals ~ all_consort$avg_grp_size, xlab="Social group s
ize", ylab="No. of unique consortship partners (residuals)", pch=19, cex.lab=1.
25, cex.axis=1.25, xlim=c(20,120), ylim=c(-3,3))
abline(lm(glmer_consorter_residuals ~ all_consort$avg_grp_size),col="black")
mtext("B", side=3, at=c(-2), line=0.5, cex=2, font=2)
```

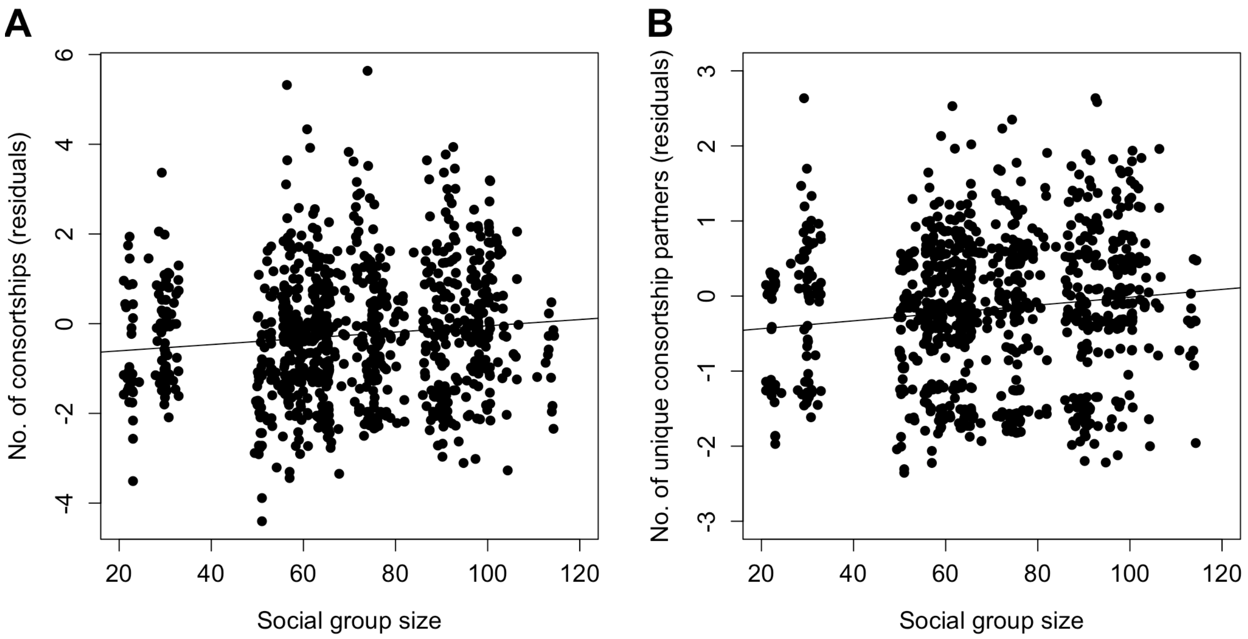


```
par(mfrow=c(1,1))
```

Beta diversity

Should we account for baboon ID in beta diversity analyses?

Four baboons were sampled twice.

```r
library(cluster) #version 2.0.4
library(reshape) #version 0.8.5
# Are samples from the same baboon more similar?
# Dataframe of all possible sample dyads:
all_dyads <- data.frame(t(combn(row.names(sid),2)), as.factor(as.numeric(bid_go
wer)))
names(all_dyads) <- c("sample_id1", "sample_id2", "same_baboon")
r <- melt(as.matrix(daisy(data.frame(rep=meta1$rep_cycle, row.names=rownames(me
ta1)), metric="gower")))
colnames(r) <-  c("sample_id1", "sample_id2", "same_rep")
# Dissimilarity matrices
bray_melt <- melt(data.matrix(bray))
colnames(bray_melt) <- c("sample_id1", "sample_id2", "bray")
unifrac_melt <- melt(data.matrix(unifrac))
colnames(unifrac_melt) <- c("sample_id1", "sample_id2", "unifrac")
all_dyads1 <- join_all(list(all_dyads, bray_melt, unifrac_melt, r), by=c("sampl
e_id1","sample_id2"))
# Control for reproductive state and cycle phase
bray_residuals <- residuals(lm(all_dyads1$bray ~ all_dyads1$same_rep))
unifrac_residuals <- residuals(lm(all_dyads1$unifrac ~ all_dyads1$same_rep))
all_dyads2 <- cbind(all_dyads1, bray_residuals, unifrac_residuals)
# Because sample sizes are not equal, use Wilcoxon rank-sum tests (Mann–Whitney
 U tests)
# Bray-Curtis dissimilarity
wilcox.test(all_dyads2$bray_residuals ~ all_dyads2$same_baboon)
```

```
##
##  Wilcoxon rank sum test with continuity correction
##
## data:  all_dyads2$bray_residuals by all_dyads2$same_baboon
## W = 1717, p-value = 0.2257
## alternative hypothesis: true location shift is not equal to 0
```

```r
# Weighted UniFrac distance
wilcox.test(all_dyads2$unifrac_residuals ~ all_dyads2$same_baboon)
```

```
##
##  Wilcoxon rank sum test with continuity correction
##
## data:  all_dyads2$unifrac_residuals by all_dyads2$same_baboon
## W = 1993, p-value = 0.395
## alternative hypothesis: true location shift is not equal to 0
```

Based on Wilcoxon rank sum tests (Mann–Whitney U tests), samples from the same baboon are no

more similar to each other than samples from different baboons.

FIGURE S3

```
library(labdsv) #version 1.8-0
library(vegan) #version 2.4-1
# Panel A
bray.pcoa <- pco(as.matrix(bray), k=3)
bray.var_pco1 <- (bray.pcoa$eig[1]/sum(bray.pcoa$eig))*100
bray.var_pco2 <- (bray.pcoa$eig[2]/sum(bray.pcoa$eig))*100
bray.points <- data.frame(bray.pco1=bray.pcoa$points[,1], bray.pco2=bray.pcoa$p
oints[,2])
meta2 <- merge(meta1, bray.points, by="row.names")
rownames(meta2) <- meta2$Row.names
meta2$Row.names <- NULL
layout(matrix(c(1,2,3,4), 2, 2, byrow = TRUE), widths=c(2, 1.5))
xpd=NA
plot(meta2$bray.pco1, meta2$bray.pco2,xlab=paste("PCO 1 (",signif(bray.var_pco1
,2),"%)",sep=""),ylab=paste("PCO 2 (",signif(bray.var_pco2,2),"%)",sep=""), pch
=ifelse(meta2$baboon_id=="B1", 19, ifelse(meta2$baboon_id=="B2",19, ifelse(meta
2$baboon_id=="B3", 19, ifelse(meta2$baboon_id=="B4", 19, 1)))), col=ifelse(meta
2$baboon_id=="B1", "red", ifelse(meta2$baboon_id=="B2","blue", ifelse(meta2$bab
oon_id=="B3", "#33A02C", ifelse(meta2$baboon_id=="B4", "orange", "black")))), x
lim=c(-0.6,0.6), ylim=c(-0.4,0.4), cex.lab=1.5, cex.axis=1.5, cex=2)
text("A", x=-0.59, y=0.39, cex=2, font=2)
# Panel B
boxplot(all_dyads2$bray_residuals ~ all_dyads2$same_baboon, ylim=c(-0.6,0.6), c
ex.axis=1.5, cex.lab=1.5, ylab="Microbiota Bray-Curtis dissimilarity (residuals
)", boxwex=0.5, pch=3, xaxt="n")
axis(1, at=c(1,2), labels=c("Same\nIndividual", "Different\nIndividuals"), cex.
axis=1.5, cex.lab=1.5, mgp=c(3,0.5,0), padj=1)
text("B", x=0.55, y=0.58, cex=2, font=2)
# Panel C
unifrac.pcoa <- pco(as.matrix(unifrac), k=3)
unifrac.var_pco1 <- (unifrac.pcoa$eig[1]/sum(unifrac.pcoa$eig))*100
unifrac.var_pco2 <- (unifrac.pcoa$eig[2]/sum(unifrac.pcoa$eig))*100
unifrac.points <- data.frame(unifrac.pco1=unifrac.pcoa$points[,1], unifrac.pco2
=unifrac.pcoa$points[,2])
meta3 <- merge(meta2, unifrac.points, by="row.names")
rownames(meta3) <- meta3$Row.names
meta3$Row.names <- NULL
plot(meta3$unifrac.pco1, meta3$unifrac.pco2,xlab=paste("PCO 1 (",signif(unifrac
.var_pco1,2),"%)",sep=""),ylab=paste("PCO 2 (",signif(unifrac.var_pco2,2),"%)",
sep=""), pch=ifelse(meta3$baboon_id=="B1", 19, ifelse(meta3$baboon_id=="B2",19,
 ifelse(meta3$baboon_id=="B3", 19, ifelse(meta3$baboon_id=="B4", 19, 1)))), col
=ifelse(meta3$baboon_id=="B1", "red", ifelse(meta3$baboon_id=="B2","blue", ifel
se(meta3$baboon_id=="B3", "#33A02C", ifelse(meta3$baboon_id=="B4", "orange", "b
lack")))), xlim=c(-0.6,0.6), ylim=c(-0.4,0.4), cex.lab=1.5, cex.axis=1.5, cex=2
)
text("C", x=-0.59, y=0.39, cex=2, font=2)
```

```
# Panel D
boxplot(all_dyads2$unifrac_residuals ~ all_dyads2$same_baboon, ylim=c(-0.6,0.6)
, cex.axis=1.5, cex.lab=1.5, ylab="Microbiota weighted UniFrac (residuals)", bo
xwex=0.5, pch=3, xaxt="n")
axis(1, at=c(1,2), labels=c("Same\nIndividual", "Different\nIndividuals"), cex.
axis=1.5, cex.lab=1.5, mgp=c(3,0.5,0), padj=1)
text("D", x=0.55, y=0.58, cex=2, font=2)
```

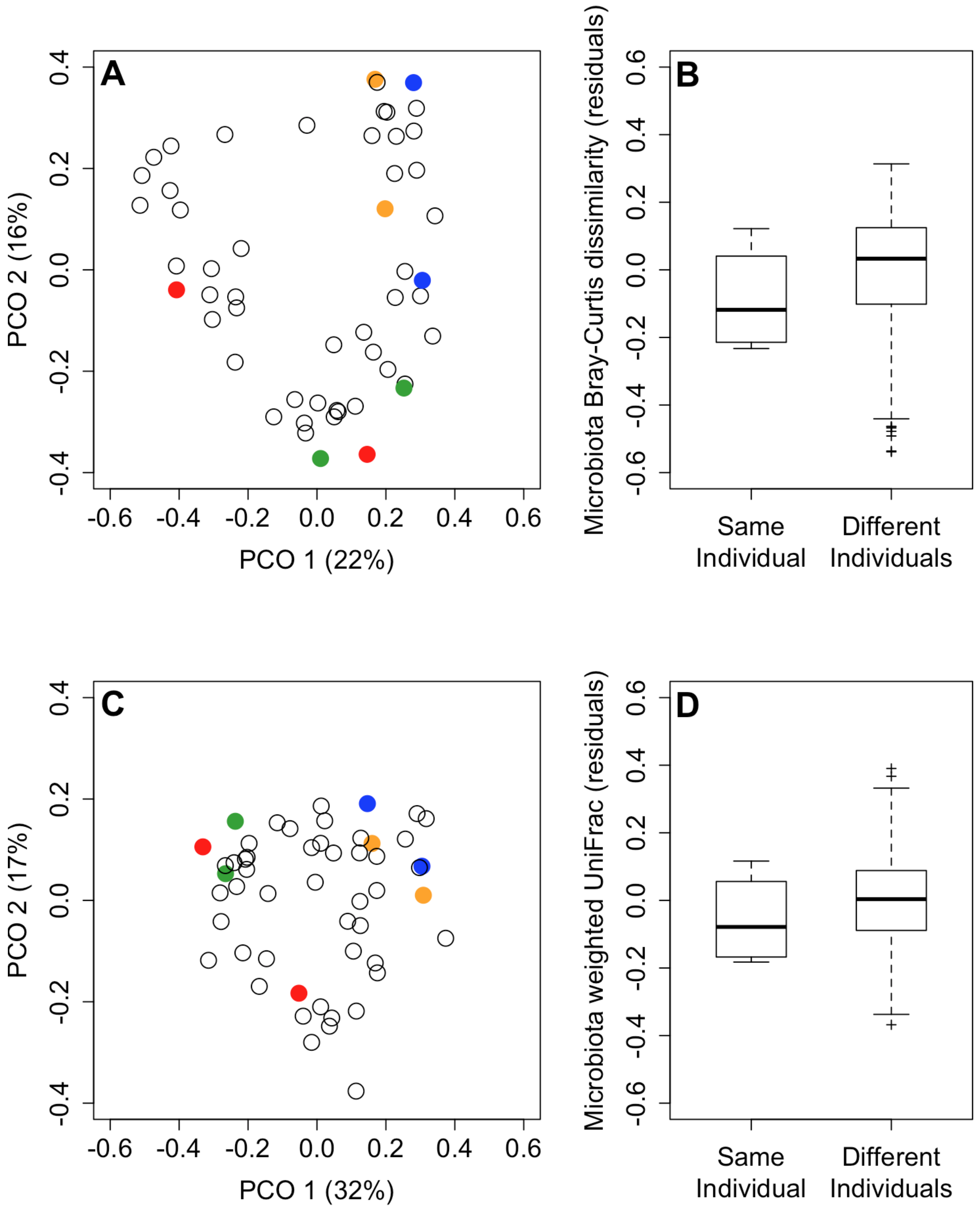

What host factors predict beta diversity?

We performed PERMANOVAs using Bray-Curtis dissimilarity and weighted UniFrac distance to identify predictors of vaginal microbial dissimilarity between samples.

Note: Due to the random iterative nature of this analysis, Pr(>F) may vary slightly with reanalyses.

```
library(vegan) #version 2.4-1
bray_noMC <- bray[rownames(bray) %in% rownames(meta_noMC), colnames(bray) %in%
rownames(meta_noMC)]
unifrac_noMC <- unifrac[rownames(unifrac) %in% rownames(meta_noMC), colnames(un
ifrac) %in% rownames(meta_noMC)]
# Make sure dissimilarity matrices and metadata dataframe are in the same order
bray_noMC_ordered <- bray_noMC[match(rownames(meta_noMC), rownames(bray_noMC)),
 match(rownames(meta_noMC), colnames(bray_noMC))]
unifrac_noMC_ordered <- unifrac_noMC[match(rownames(meta_noMC), rownames(unifra
c_noMC)), match(rownames(meta_noMC), colnames(unifrac_noMC))]
# PERMANOVAs: reproductive state
adonis(bray_noMC_ordered ~ meta_noMC$rep_state, permutations=10000, method="bra
y")$aov.tab
```

```
## Permutation: free
## Number of permutations: 10000
##
## Terms added sequentially (first to last)
##
##                     Df SumsOfSqs MeanSqs F.Model      R2    Pr(>F)
## meta_noMC$rep_state  2    2.8878 1.44388  5.2415 0.17925 9.999e-05 ***
## Residuals           48   13.2225 0.27547         0.82075
## Total               50   16.1103                 1.00000
## ---
## Signif. codes:  0 '***' 0.001 '**' 0.01 '*' 0.05 '.' 0.1 ' ' 1
```

```
adonis(unifrac_noMC_ordered ~ meta_noMC$rep_state, permutations=10000)$aov.tab
```

```
## Permutation: free
## Number of permutations: 10000
##
## Terms added sequentially (first to last)
##
##                     Df SumsOfSqs MeanSqs F.Model      R2    Pr(>F)
## meta_noMC$rep_state  2    1.1493 0.57464  5.8078 0.19484 9.999e-05 ***
## Residuals           48    4.7493 0.09894         0.80516
## Total               50    5.8985                 1.00000
## ---
## Signif. codes:  0 '***' 0.001 '**' 0.01 '*' 0.05 '.' 0.1 ' ' 1
```

```
# Repeat PERMANOVAs on just cycling females (n=28)
bray_C <- bray[rownames(bray) %in% rownames(meta_C), colnames(bray) %in% rownam
es(meta_C)]
unifrac_C <- unifrac[rownames(unifrac) %in% rownames(meta_C), colnames(unifrac)
 %in% rownames(meta_C)]
bray_C_ordered <- bray_C[match(rownames(meta_C), rownames(bray_C)), match(rowna
mes(meta_C), colnames(bray_C))]
unifrac_C_ordered <- unifrac_C[match(rownames(meta_C), rownames(unifrac_C)), ma
tch(rownames(meta_C), colnames(unifrac_C))]
adonis(dist(bray_C_ordered) ~ meta_C$cycle_phase, permutations=10000, method="b
ray")$aov.tab
```

```
## Permutation: free
## Number of permutations: 10000
##
## Terms added sequentially (first to last)
##
##                    Df SumsOfSqs MeanSqs F.Model      R2    Pr(>F)
## meta_C$cycle_phase  3    12.489  4.1629  5.0873 0.38872 9.999e-05 ***
## Residuals          24    19.639  0.8183         0.61128
## Total              27    32.127                 1.00000
## ---
## Signif. codes:  0 '***' 0.001 '**' 0.01 '*' 0.05 '.' 0.1 ' ' 1
```

```
adonis(unifrac_C_ordered ~ meta_C$cycle_phase, permutations=10000)$aov.tab
```

```
## Permutation: free
## Number of permutations: 10000
##
## Terms added sequentially (first to last)
##
##                    Df SumsOfSqs  MeanSqs F.Model     R2 Pr(>F)
## meta_C$cycle_phase  3   0.65761 0.219204  2.3053 0.2237 0.0033 **
## Residuals          24   2.28208 0.095087         0.7763
## Total              27   2.93969                  1.0000
## ---
## Signif. codes:  0 '***' 0.001 '**' 0.01 '*' 0.05 '.' 0.1 ' ' 1
```

FIGURE 4A

```
meta3$rep_state <- factor(meta3$rep_state, levels = c("C", "P", "PPA", "MC"))
par(fig=c(0,0.8,0,0.8), new=TRUE, xpd=TRUE, mar=c(10, 4, 1, 4))
plot(meta3$bray.pco1, meta3$bray.pco2, xlab=paste("PCO 1 (",signif(bray.var_pco
1,2),"%)",sep=""), ylab=paste("PCO 2 (",signif(bray.var_pco2,2),"%)",sep=""), p
ch=19, col= c("darkturquoise", "darkorange", "purple", "navy")[as.numeric(meta3
$rep_state)], xlim=c(-0.6,0.6), ylim=c(-0.5,0.5), cex.lab=1.25, cex.axis=1.25)
legend('bottom', inset=c(1,-0.55), c("Ovarian Cycling","Pregnant","Postpartum A
menorrhea","Miscarrying"), col=c("darkturquoise","darkorange","purple","navy"),
 pch=19, cex=1, xpd=TRUE)
mtext("A", side=3, at=c(-0.85), line=4, cex=2, font=2)
meta4_noMC <- droplevels(subset(meta3, meta3$rep_state != "MC"))
par(fig=c(0,0.8,0.49,0.95), new=TRUE, xpd=TRUE)
boxplot(meta4_noMC$bray.pco1 ~ meta4_noMC$rep_state, horizontal=TRUE, ylim=c(-0
.6,0.6), axes=FALSE, col=c("darkturquoise", "darkorange", "purple"), pch=21, ou
tbg=c("darkturquoise","darkorange","purple"))
par(fig=c(0.578,0.95,0,0.8),new=TRUE, xpd=TRUE)
boxplot(meta4_noMC$bray.pco2 ~ meta4_noMC$rep_state, ylim=c(-0.5,0.5), axes=FAL
SE, col=c("darkturquoise","darkorange","purple"), pch=21, outbg=c("darkturquois
e","darkorange", "purple"))
```

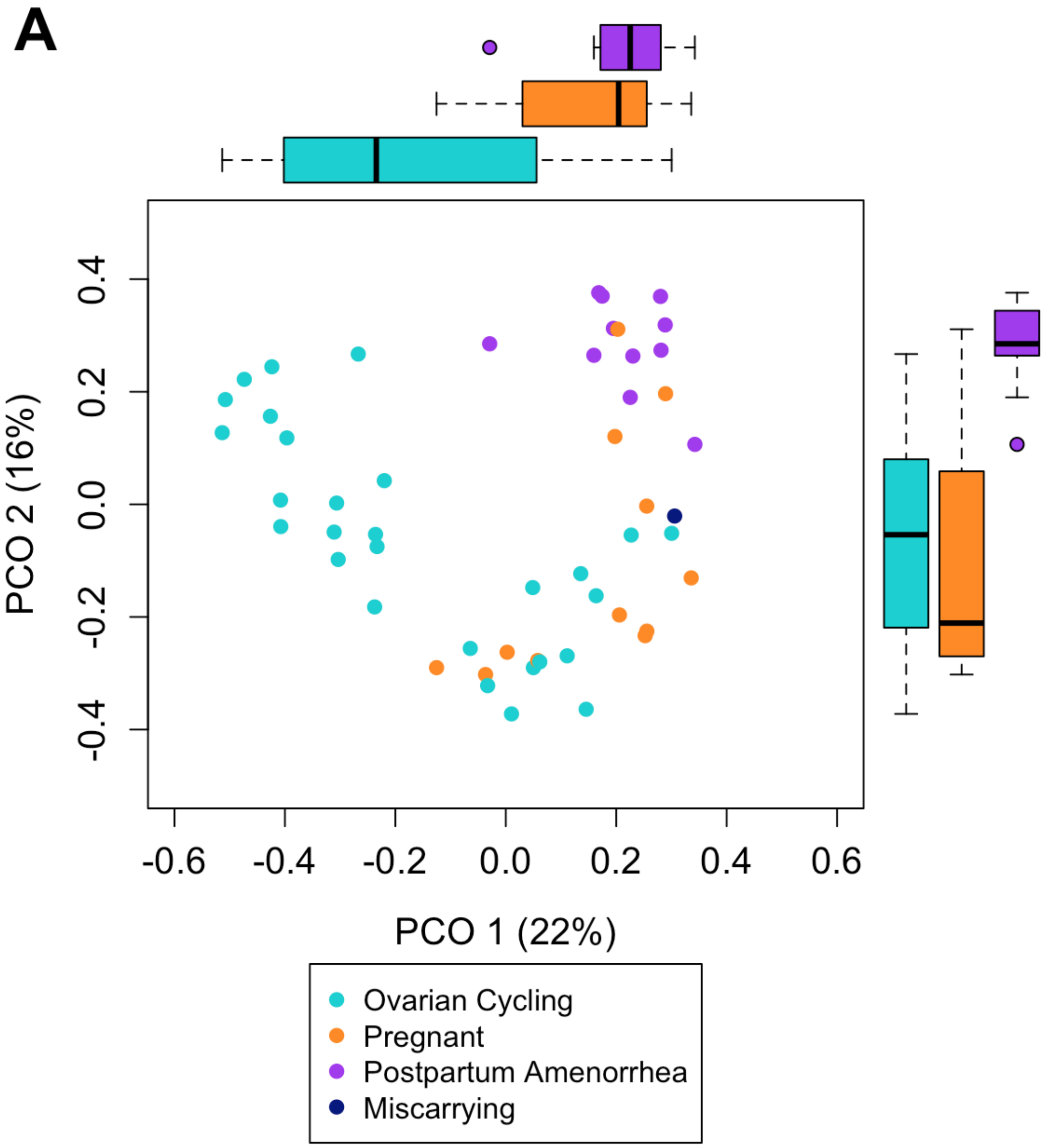
A
PCO 2 (16%)
0.4
0.2
0.0
-0.2
-0.4
-0.6
-0.4
-0.2
0.0
0.2
0.4
0.6
PCO 1 (22%)
Ovarian Cycling
Pregnant
Postpartum Amenorrhea
Miscarrying

FIGURE 4B

```
bray_C.pcoa <- pco(as.matrix(bray_C), k=3)
bray_C.var_pco1 <- (bray_C.pcoa$eig[1]/sum(bray_C.pcoa$eig))*100
bray_C.var_pco2 <- (bray_C.pcoa$eig[2]/sum(bray_C.pcoa$eig))*100
bray_C.points <- data.frame(bray_C.pco1=bray_C.pcoa$points[,1], bray_C.pco2=bra
y_C.pcoa$points[,2])
meta_C1 <- merge(meta_C, bray_C.points, by="row.names")
rownames(meta_C1) <- meta_C1$Row.names
meta_C1$Row.names <- NULL
meta_C1$cycle_phase <- factor(meta_C1$cycle_phase, levels = c("A", "S", "O", "D
"))
par(fig=c(0,0.8,0,0.8), new=TRUE, xpd=TRUE, mar=c(10, 4, 1, 4))
plot(meta_C1$bray_C.pco1, meta_C1$bray_C.pco2, xlab=paste("PCO 1 (",signif(bray
_C.var_pco1,2),"%)",sep=""), ylab=paste("PCO 2 (",signif(bray_C.var_pco2,2),"%)
",sep=""), pch=19, col= c("red", "gold3", "#33A02C", "blue")[as.numeric(meta_C1
$cycle_phase)], xlim=c(-0.6,0.6), ylim=c(-0.5,0.5), cex.lab=1.25, cex.axis=1.25
)
legend('bottom', inset=c(1,-0.55), c("Anestrus","Swelling","Peri-Ovulation","De
turgescence"), col=c("red", "gold3", "#33A02C", "blue"), pch=19, cex=1, xpd=TRU
E)
mtext("B", side=3, at=c(-0.85), line=4, cex=2, font=2)
par(fig=c(0,0.8,0.49,0.95), new=TRUE, xpd=TRUE)
boxplot(meta_C1$bray_C.pco1 ~ meta_C1$cycle_phase, horizontal=TRUE, ylim=c(-0.6
,0.6), axes=FALSE, col=c("red", "gold3", "#33A02C", "blue"), pch=21, outbg=c("r
ed", "gold3", "#33A02C", "blue"))
par(fig=c(0.578,0.95,0,0.8),new=TRUE, xpd=TRUE)
boxplot(meta_C1$bray_C.pco2 ~ meta_C1$cycle_phase, ylim=c(-0.5,0.5), axes=FALSE
, col=c("red", "gold3", "#33A02C", "blue"), pch=21, outbg=c("red", "gold3", "#3
3A02C", "blue"))
```

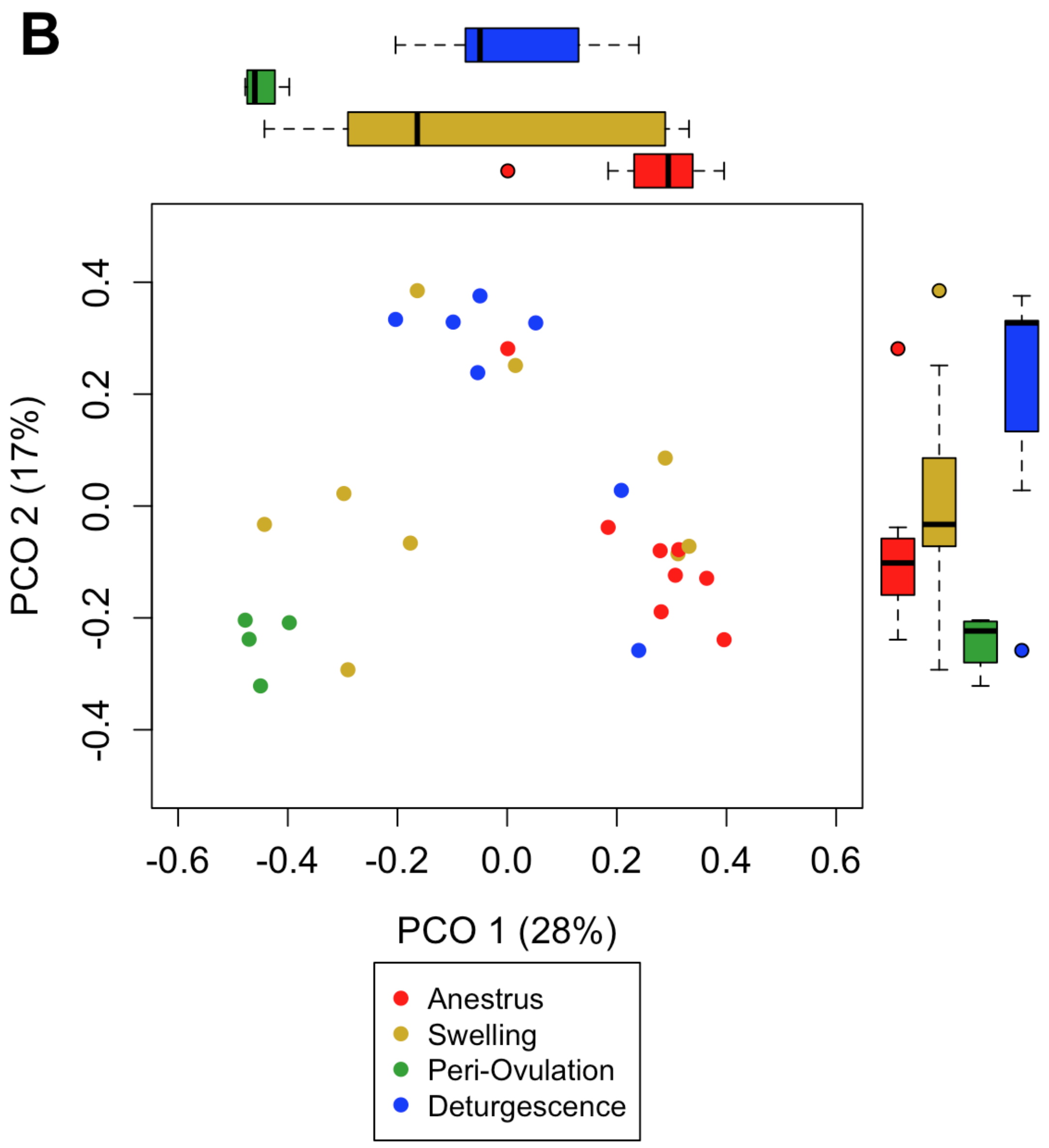
B
-0.6
-0.4
-0.2
0.0
0.2
0.4
0.6
PCO 1 (28%)
PCO 2 (17%)
Anestrus
Swelling
Peri-Ovulation
Deturgescence

FIGURE S6A

```
par(fig=c(0,0.8,0,0.8), new=TRUE, xpd=TRUE, mar=c(10, 4, 1, 4))
plot(meta3$unifrac.pco1, meta3$unifrac.pco2, xlab=paste("PCO 1 (",signif(unifra
c.var_pco1,2),"%)",sep=""), ylab=paste("PCO 2 (",signif(unifrac.var_pco2,2),"%)
",sep=""), pch=19, col= c("darkturquoise", "darkorange", "purple", "navy")[as.n
umeric(meta3$rep_state)], xlim=c(-0.4,0.4), ylim=c(-0.4,0.4), cex.lab=1.25, cex
.axis=1.25)
legend('bottom', inset=c(1,-0.55), c("Ovarian Cycling","Pregnant","Postpartum A
menorrhea","Miscarrying"), col=c("darkturquoise","darkorange","purple","navy"),
 pch=19, cex=1, xpd=TRUE)
mtext("A", side=3, at=c(-0.5), line=4, cex=2, font=2)
par(fig=c(0,0.8,0.49,0.95), new=TRUE, xpd=TRUE)
boxplot(meta4_noMC$unifrac.pco1 ~ meta4_noMC$rep_state, horizontal=TRUE, ylim=c
(-0.4,0.4), axes=FALSE, col=c("darkturquoise", "darkorange", "purple"), pch=21,
 outbg=c("darkturquoise","darkorange","purple"))
par(fig=c(0.578,0.95,0,0.8),new=TRUE, xpd=TRUE)
boxplot(meta4_noMC$unifrac.pco2 ~ meta4_noMC$rep_state, ylim=c(-0.4,0.4), axes=
FALSE, col=c("darkturquoise","darkorange","purple"), pch=21, outbg=c("darkturqu
oise","darkorange", "purple"))
```

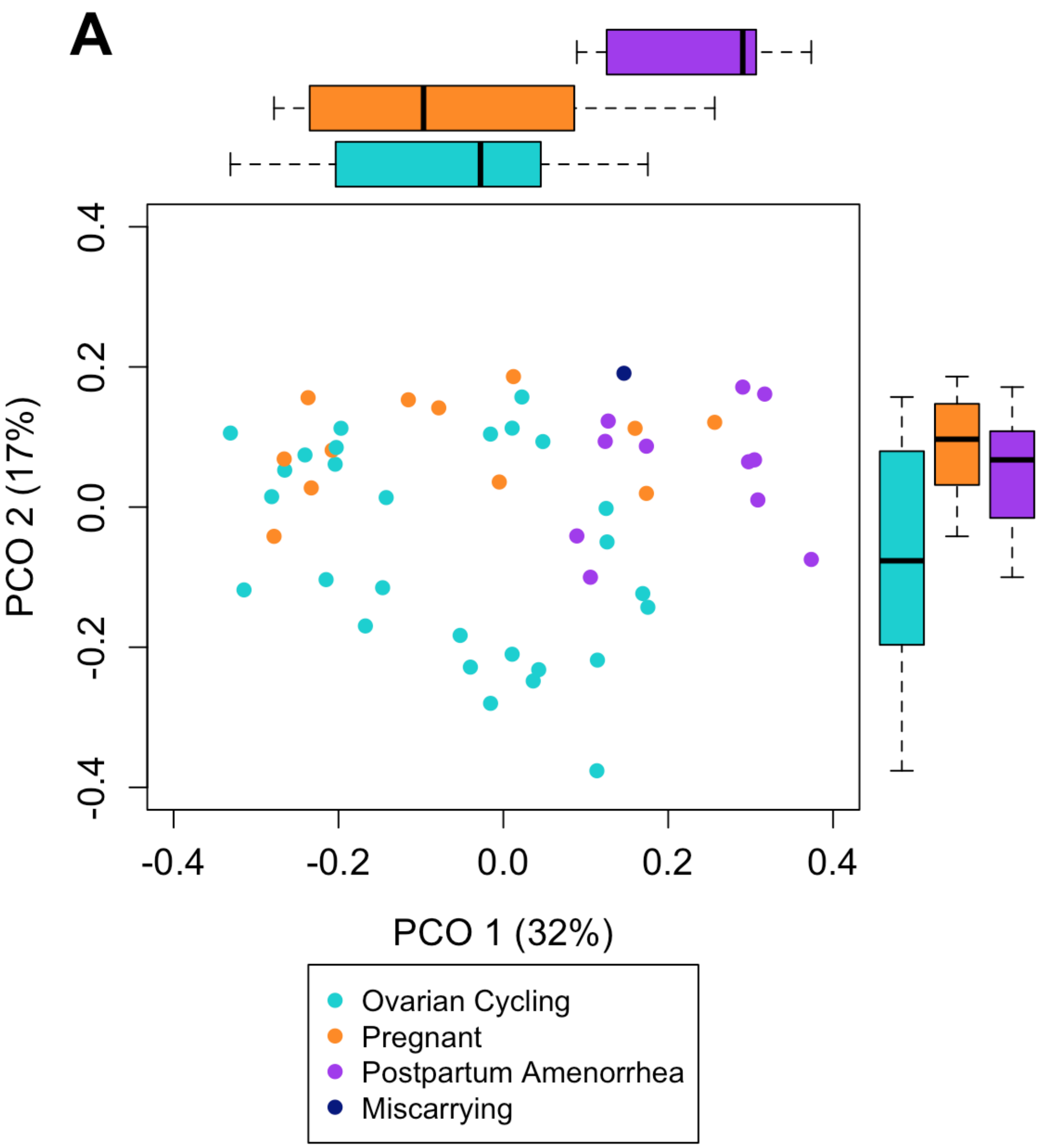
A
0.4
0.2
0.0
-0.2
-0.4
PCO 2 (17%)
-0.4
-0.2
0.0
0.2
0.4
PCO 1 (32%)
Ovarian Cycling
Pregnant
Postpartum Amenorrhea
Miscarrying

FIGURE S6B

```
unifrac_C.pcoa <- pco(as.matrix(unifrac_C), k=3)
unifrac_C.var_pco1 <- (unifrac_C.pcoa$eig[1]/sum(unifrac_C.pcoa$eig))*100
unifrac_C.var_pco2 <- (unifrac_C.pcoa$eig[2]/sum(unifrac_C.pcoa$eig))*100
unifrac_C.points <- data.frame(unifrac_C.pco1=unifrac_C.pcoa$points[,1], unifra
c_C.pco2=unifrac_C.pcoa$points[,2])
meta_C1 <- merge(meta_C, unifrac_C.points, by="row.names")
rownames(meta_C1) <- meta_C1$Row.names
meta_C1$Row.names <- NULL
meta_C1$cycle_phase <- factor(meta_C1$cycle_phase, levels = c("A", "S", "O", "D
"))
par(fig=c(0,0.8,0,0.8), new=TRUE, xpd=TRUE, mar=c(10, 4, 1, 4))
plot(meta_C1$unifrac_C.pco1, meta_C1$unifrac_C.pco2, xlab=paste("PCO 1 (",signi
f(unifrac_C.var_pco1,2),"%)",sep=""), ylab=paste("PCO 2 (",signif(unifrac_C.var
_pco2,2),"%)",sep=""), pch=19, col= c("red", "gold3", "#33A02C", "blue")[as.num
eric(meta_C1$cycle_phase)], xlim=c(-0.4,0.4), ylim=c(-0.4,0.4), cex.lab=1.25, c
ex.axis=1.25)
legend('bottom', inset=c(1,-0.55), c("Anestrus","Swelling","Peri-Ovulation","De
turgescence"), col=c("red", "gold3", "#33A02C", "blue"), pch=19, cex=1, xpd=TRU
E)
mtext("B", side=3, at=c(-0.5), line=4, cex=2, font=2)
par(fig=c(0,0.8,0.49,0.95), new=TRUE, xpd=TRUE)
boxplot(meta_C1$unifrac_C.pco1 ~ meta_C1$cycle_phase, horizontal=TRUE, ylim=c(-
0.4,0.4), axes=FALSE, col=c("red", "gold3", "#33A02C", "blue"), pch=21, outbg=c
("red", "gold3", "#33A02C", "blue"))
par(fig=c(0.578,0.95,0,0.8),new=TRUE, xpd=TRUE)
boxplot(meta_C1$unifrac_C.pco2 ~ meta_C1$cycle_phase, ylim=c(-0.4,0.4), axes=FA
LSE, col=c("red", "gold3", "#33A02C", "blue"), pch=21, outbg=c("red", "gold3",
"#33A02C", "blue"))
```

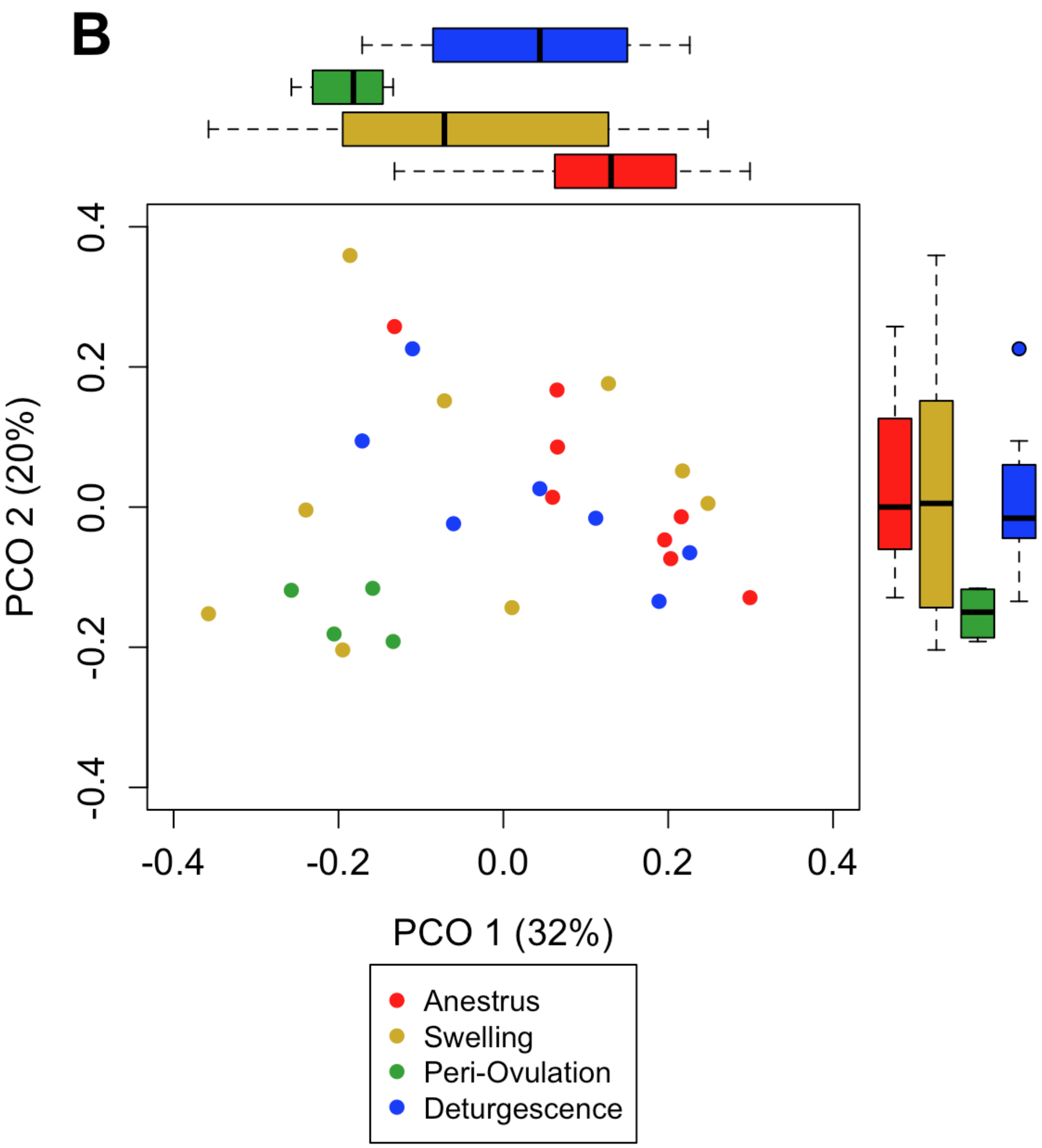

LEfSe

FIGURE 5A

The relationships between taxonomic levels were added to the figure in Microsoft PowerPoint. The significant LEfSe comparisons between reproductive states were also added in Microsoft PowerPoint.

```
# Combine all taxa relative abundance dataframes into a single dataframe
data <- rbind(phylum,class,order,family,genus,species)
# Remove unkown taxa
unknown_taxa <- grep("uncultured|Ambiguous_tax|Unknown",rownames(data), value=T
RUE)
data1 <- subset(data, !(rownames(data) %in% unknown_taxa))
other_taxa <- grep("^.+(Other)$",rownames(data1), value=TRUE)
data2 <- subset(data1, !(rownames(data1) %in% other_taxa))
# Do some modification of taxa names
rownames(data2) <- gsub("k__|p__|c__|o__|f__|g__|s__", "", rownames(data2))
rownames(data2) <- gsub(";", ".", rownames(data2))
rownames(data2) <- gsub("]", "_", rownames(data2))
rownames(data2) <- gsub("[", "_", rownames(data2), fixed=TRUE)
rownames(data2) <- gsub("-", "_", rownames(data2))
# Pick out taxa based on lefse list
data_lefse_r <- data2[rownames(data2) %in% rownames(lefse_rep),]
data_lefse_c <- data2[rownames(data2) %in% rownames(lefse_cycle),]
# Merge samples by rep. state
rep <- data.frame(rep=meta$rep_state, row.names=row.names(meta))
data_lefse_r1 <- t(data_lefse_r)
data_lefse_r2 <- merge(rep, data_lefse_r1, by="row.names")
rownames(data_lefse_r2) <- data_lefse_r2[,1]
data_lefse_r3 <- data_lefse_r2[,-1]
data_lefse_r3$rep <- as.factor(data_lefse_r3$rep)
data_lefse_r4 <- droplevels(subset(data_lefse_r3, rownames(data_lefse_r3) != "V
32"))
data_lefse_r4_agg <- aggregate(data_lefse_r4[2:ncol(data_lefse_r4)], by=list(da
ta_lefse_r4$rep), FUN=mean, data=data_lefse_r4)
colnames(data_lefse_r4_agg)[1] <- "state"
# Merge samples by cycle phase
cycle <- data.frame(cycle=meta$cycle_phase, row.names = row.names(meta))
cycle1 <- subset(cycle, cycle %in% c("A","S","O","D"))
data_lefse_c1 <- t(data_lefse_c)
data_lefse_c2 <- merge(cycle1, data_lefse_c1, by="row.names")
rownames(data_lefse_c2) <- data_lefse_c2[,1]
data_lefse_c3 <- data_lefse_c2[,-1]
data_lefse_c3$cycle <- as.factor(data_lefse_c3$cycle)
data_lefse_c3_agg <- aggregate(data_lefse_c3[2:ncol(data_lefse_c3)], by=list(da
ta_lefse_c3$cycle), FUN=mean, data=data_lefse_c3)
colnames(data_lefse_c3_agg)[1] <- "state"
# Melt dataframes and sort
data_lefse_r_final <- melt(data_lefse_r4_agg)
colnames(data_lefse_r_final) <- c("state", "taxa", "average")
data_lefse_c_final <- melt(data_lefse_c3_agg)
colnames(data_lefse_c_final) <- c("state", "taxa", "average")
data_lefse_r_final1 <- data_lefse_r_final[order(as.character(data_lefse_r_final
```

```
$taxa)),]
data_lefse_c_final1 <- data_lefse_c_final[order(as.character(data_lefse_c_final
$taxa)),]
# Modify taxa names
data_lefse_r_final1$taxa <- gsub("\\.", "|", data_lefse_r_final1$taxa)
data_lefse_r_final1$taxa <- gsub("Bacteria\\|", "", data_lefse_r_final1$taxa)
data_lefse_r_final1$taxa <- gsub("_", " ", data_lefse_r_final1$taxa)
data_lefse_c_final1$taxa <- gsub("\\.", "|", data_lefse_c_final1$taxa)
data_lefse_c_final1$taxa <- gsub("Bacteria\\|", "", data_lefse_c_final1$taxa)
data_lefse_c_final1$taxa <- gsub("Erysipelotrichaceae_UCG_001", "Erysipelotrich
aceae UCG-001", data_lefse_c_final1$taxa)
data_lefse_c_final1$taxa <- gsub("_", " ", data_lefse_c_final1$taxa)
# Add a column with phylum (for coloring circles later)
library(splitstackshape)
data_lefse_r_final2 <- data.frame(cSplit(data_lefse_r_final1, "taxa", sep="|",
drop=FALSE))
data_lefse_r_final3 <- subset(data_lefse_r_final2, select = -c(taxa_2,taxa_3,ta
xa_4,taxa_5))
names(data_lefse_r_final3)[4] <- "phylum"
data_lefse_r_final3$phylum <- with(data_lefse_r_final3, factor(phylum, levels =
 rev(levels(phylum))))
data_lefse_r_final3$taxa <- as.factor(data_lefse_r_final3$taxa)
data_lefse_r_final3$taxa <- with(data_lefse_r_final3, factor(taxa, levels = rev
(levels(taxa))))
data_lefse_r_final3$average <- data_lefse_r_final3$average*100
data_lefse_c_final2 <- data.frame(cSplit(data_lefse_c_final1, "taxa", sep="|",
drop=FALSE))
data_lefse_c_final3 <- subset(data_lefse_c_final2, select = -c(taxa_2,taxa_3,ta
xa_4,taxa_5,taxa_6))
names(data_lefse_c_final3)[4] <- "phylum"
data_lefse_c_final3$phylum <- with(data_lefse_c_final3, factor(phylum, levels =
 rev(levels(phylum))))
data_lefse_c_final3$taxa <- as.factor(data_lefse_c_final3$taxa)
data_lefse_c_final3$taxa <- with(data_lefse_c_final3, factor(taxa, levels = rev
(levels(taxa))))
data_lefse_c_final3$average <- data_lefse_c_final3$average*100
data_lefse_c_final4 <- droplevels(subset(data_lefse_c_final3, phylum != "Archae
a"))
# Add column with significant comparisons
library(ggplot2)
data_lefse_r_final4 <- subset(data_lefse_r_final3, average > 0)
data_lefse_r_final4$state <- factor(data_lefse_r_final4$state, c("C", "P", "PPA
"))
ggplot(data_lefse_r_final4, aes(state,taxa)) +
  geom_point(aes(fill=factor(phylum), size=average), shape=21) +
  theme_bw() + ylab("") + xlab("") + guides(fill = FALSE) +
```

```
  scale_size_continuous(range = c(0.5,9), breaks = c(0.01,0.1,1,5,10,20,30,40,50), labels = c('\u2264 0.01%',"0.1%","1%","5%", "10%","20%", "30%","40%",'\u2265 50%'), name="Average\nRelative\nAbundance") +
  theme(legend.position="right", legend.text.align=0, legend.title.align=0.5, legend.background = element_rect(fill="white", size=.5, linetype="solid", color="black"), axis.text.x=element_text(angle=45, size=12, vjust=1, hjust=1)) +
  scale_fill_manual(values = c("#A65628","#FFFF33","#999999","#FF7F00","#984EA3","#4DAF4A","#377EB8")) +
  scale_x_discrete(labels=c("C" = "Ovarian\nCycling", "P" = "Pregnant", "PPA" = "Postpartum\nAmenorrhea", "D"="Deturgescence"))
```

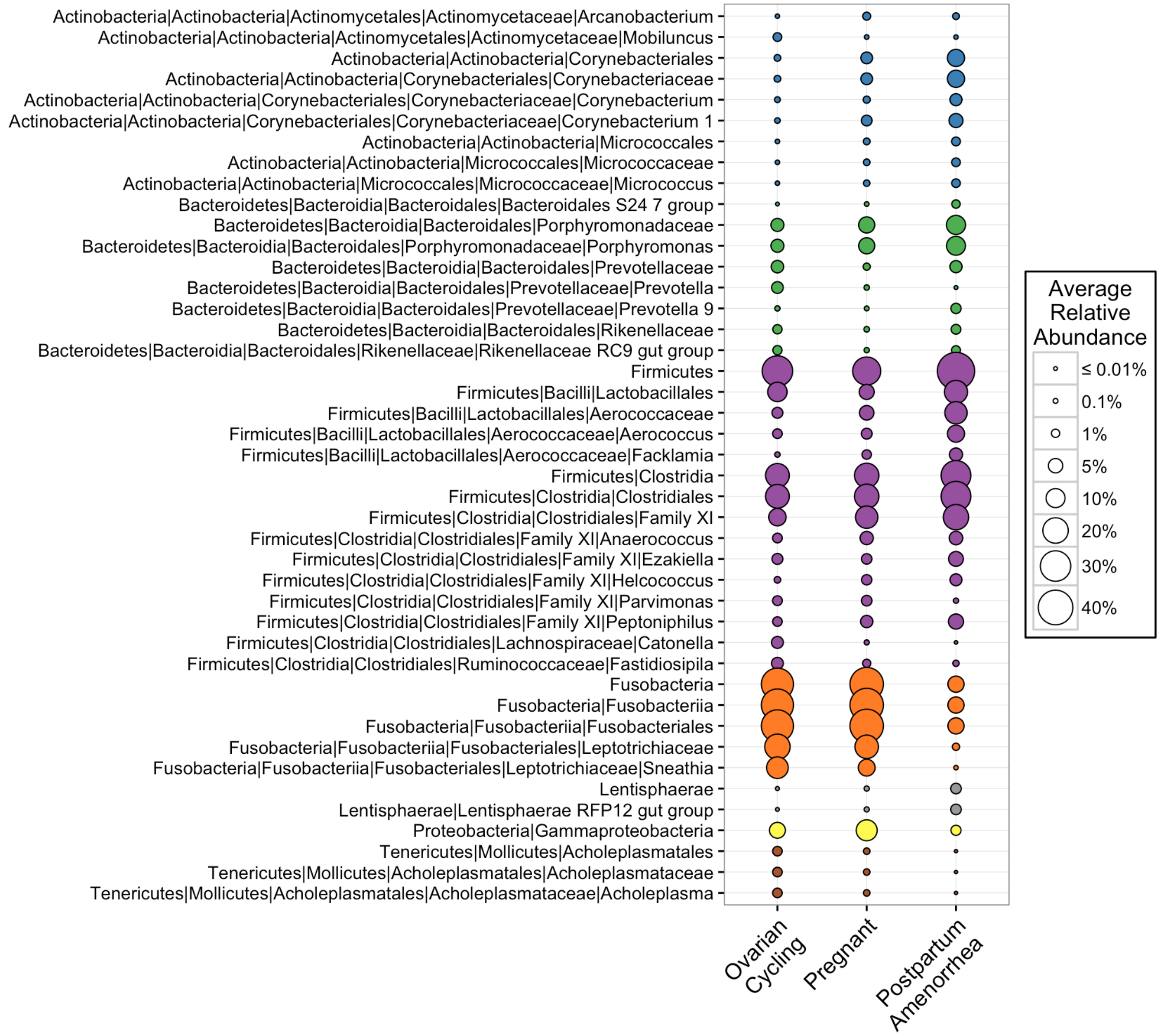

FIGURE 5B

The relationships between taxonomic levels were added to the figure in Microsoft PowerPoint. The significant LEfSe comparisons between ovarian cycle phases were also added in Microsoft PowerPoint.

```
data_lefse_c_final5 <- subset(data_lefse_c_final4, average > 0)
data_lefse_c_final5$state <- factor(data_lefse_c_final5$state, c("A", "S", "O",
 "D"))
ggplot(data_lefse_c_final5, aes(state,taxa)) +
  geom_point(aes(fill=factor(phylum), size=average), shape=21) +
  theme_bw() + ylab("") + xlab("") + guides(fill = FALSE) +
  scale_size_continuous(range = c(0.5,9), breaks = c(0.01,0.1,1,5,10,20,30,40,5
0), labels = c('\u2264 0.01%',"0.1%","1%","5%", "10%","20%", "30%","40%",'\u226
5 50%'), name="Average\nRelative\nAbundance") +
  theme(legend.position="right",
        legend.text.align=0, legend.title.align=0.5, legend.background = elemen
t_rect(fill="white", size=.5, linetype="solid", color="black"), axis.text.x=ele
ment_text(angle=45, size=12, vjust=1, hjust=1)) +
  scale_fill_manual(values = c("#FFFF33","#FF7F00","#984EA3","#4DAF4A","#377EB8
")) +
  scale_x_discrete(labels=c("A" = "Anestrus", "S" = "Swelling", "O" = "Peri-Ovu
lation", "D"="Deturgescence"))
```

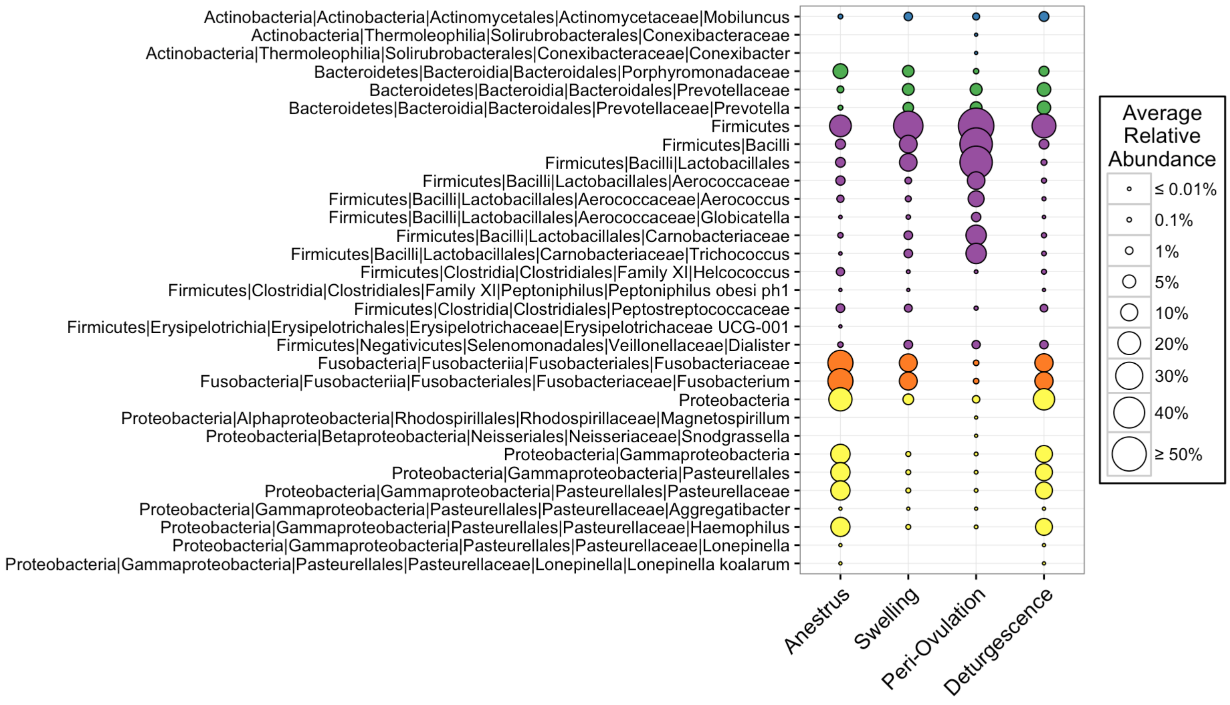

What is the mean relative abundance of the order Lactobacillales in peri-ovulatory females?

```
# Mean
subset(data_lefse_c_final4, taxa=="Firmicutes|Bacilli|Lactobacillales" & state=
="O")$average
```

```
## [1] 44.68015
```

```
# Standard Deviation
sd(data_lefse_c3[c("V51","V39","V10","V50"),"Bacteria.Firmicutes.Bacilli.Lactob
acillales"])*100
```

```
## [1] 10.13213
```

FIGURE S7

```
# Remove non-normal cycle days (normal days: -22 through +12)
cycle_norm <- droplevels(subset(meta_C, cycle_day >= -22))
cycle_norm1 <- data.frame(cycle_day=cycle_norm$"cycle_day", sample_id = rowname
s(cycle_norm))
data1 <- t(phylum)
data2 <- as.data.frame(subset(data1, rownames(data1) %in% cycle_norm1$sample_id
))
data2[,"sample_id"] <- rownames(data2)
cycle_norm_taxa <- merge(cycle_norm1, data2, by="sample_id")
firm <- cycle_norm_taxa[,c(1,2,grep("Firmicutes",colnames(cycle_norm_taxa)))]
firm[,"phylum"] <- "Firmicutes"
colnames(firm) <- c("sample_id", "cycle_day_norm", "relative_abundance", "phylu
m")
fuso <- cycle_norm_taxa[,c(1,2,grep("Fusobacteria",colnames(cycle_norm_taxa)))]
fuso[,"phylum"] <- "Fusobacteria"
colnames(fuso) <- c("sample_id", "cycle_day_norm", "relative_abundance", "phylu
m")
firm_fuso <- rbind(firm, fuso)
firm_fuso$phylum <- as.factor(firm_fuso$phylum)
firm_fuso$relative_abundance <- firm_fuso$relative_abundance * 100
# Plot
par(mar=c(5.1, 5.1, 4.1, 2.1), xpd=TRUE) #c(bottom, left, top, right)
plot(firm_fuso$cycle_day_norm, firm_fuso$relative_abundance,
     xlim=c(-22, 12), ylim=c(-20,100), xlab="Day in ovarian cycle", ylab = "Rel
ative phylum abundance (%)",
     cex.axis= 1.5, cex.lab = 1.5, type="n")
firmicutes <- subset(firm_fuso, phylum=="Firmicutes")
firmicutes <- firmicutes[order(firmicutes$cycle_day_norm),]
fusobacteria <- subset(firm_fuso, phylum=="Fusobacteria")
fusobacteria <- fusobacteria[order(fusobacteria$cycle_day_norm),]
xx <- seq(-21,11, length.out=1000)
a <- data.frame(cycle_day_norm = xx)
fuso_fit <- lm(relative_abundance ~ poly(cycle_day_norm,2,raw=FALSE), data=fuso
bacteria)
x <- predict(fuso_fit, newdata=a, interval=c("confidence"), level = 0.95, type=
"response")
polygon(c(rev(xx), xx), c(rev(x[ ,3]), x[ ,2]),
        col = adjustcolor('grey80',alpha.f=0.5), border = NA)
yy <- seq(-21,11, length.out=500)
b <- data.frame(cycle_day_norm = yy)
firm_fit <- lm(relative_abundance ~ poly(cycle_day_norm,2,raw=FALSE), data=firm
icutes)
y <- predict(firm_fit, newdata=b, interval=c("confidence"), level = 0.95, type=
"response")
polygon(c(rev(yy), yy), c(rev(y[ ,3]), y[ ,2]),
        col = adjustcolor('grey80',alpha.f=0.5), border = NA)
```

```
lines(xx, x[,"fit"], col='#FF7F00', lwd=1.5)
lines(yy, y[,"fit"], col='#984EA3', lwd=1.5)
points(firm_fuso$cycle_day_norm, firm_fuso$relative_abundance,
       col= c("#984EA3", "#FF7F00")[as.numeric(firm_fuso$phylum)],
       pch=c(19,17)[as.numeric(firm_fuso$phylum)])
legend("topright", inset=c(0.015,0.015), legend=c("Firmicutes","Fusobacteria"),

       pch=c(19,17), col=c("#984EA3", "#FF7F00"), cex=1.25)
```

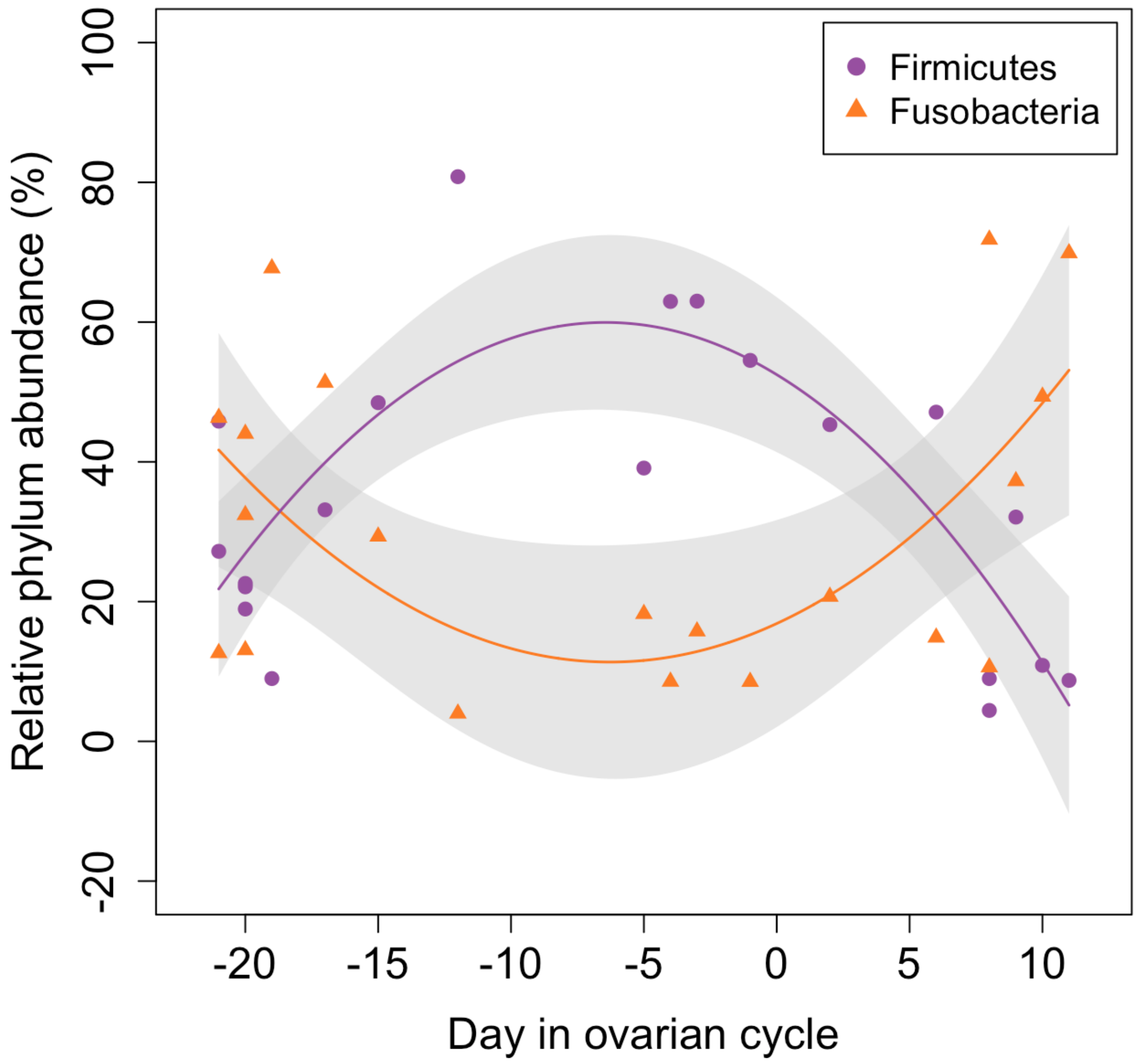

Vaginal pH

Is there a significant difference in vaginal pH between reproductive states and ovarian cycle phases?

```
kruskal.test(ph$ph ~ ph$rep_cycle)
```

```
##
##  Kruskal-Wallis rank sum test
##
## data:  ph$ph by ph$rep_cycle
## Kruskal-Wallis chi-squared = 11.782, df = 4, p-value = 0.01905
```

Yes.

FIGURE S8

```
mean_ph <- aggregate(ph$ph, list(ph$rep_cycle), mean)
colnames(mean_ph) <- c("rep_cycle","mean")
sd_ph <- aggregate(ph$ph, list(ph$rep_cycle), sd)
colnames(sd_ph) <- c("rep_cycle","sd")
ph1 <- merge(mean_ph, sd_ph, by="rep_cycle")
ph1$rep_cycle <- factor(ph1$rep_cycle, levels = c("A", "S", "D", "P", "PPA"))
ph1 <- ph1[order(ph1$rep_cycle),]
plot(as.numeric(ph1$rep_cycle), ph1$mean, ylim=c(4,10), xlim=c(0.5,5.5), ylab="
Vaginal pH", xlab=NA, cex.axis=1.25, cex.lab=1.25, xaxt="n", type="n")
axis(1, at=c(1,2,3,4,5), labels=c("A","S","D","P","PPA"), cex.axis=1.25, cex.la
b=1.25)
arrows(as.numeric(ph1[1,1]), ph1[1,2]-ph1[1,3], as.numeric(ph1[1,1]), ph1[1,2]+
ph1[1,3], length=0.05, angle=90, code=3)
arrows(as.numeric(ph1[2,1]), ph1[2,2]-ph1[2,3], as.numeric(ph1[2,1]), ph1[2,2]+
ph1[2,3], length=0.05, angle=90, code=3)
arrows(as.numeric(ph1[3,1]), ph1[3,2]-ph1[3,3], as.numeric(ph1[3,1]), ph1[3,2]+
ph1[3,3], length=0.05, angle=90, code=3)
arrows(as.numeric(ph1[4,1]), ph1[4,2]-ph1[4,3], as.numeric(ph1[4,1]), ph1[4,2]+
ph1[4,3], length=0.05, angle=90, code=3)
arrows(as.numeric(ph1[5,1]), ph1[5,2]-ph1[5,3], as.numeric(ph1[5,1]), ph1[5,2]+
ph1[5,3], length=0.05, angle=90, code=3)
points(as.numeric(ph1$rep_cycle), ph1$mean, type = "p", bg=c("red","gold3", "bl
ue","#FFA500","#800080"), pch=21,cex=1.5)
```

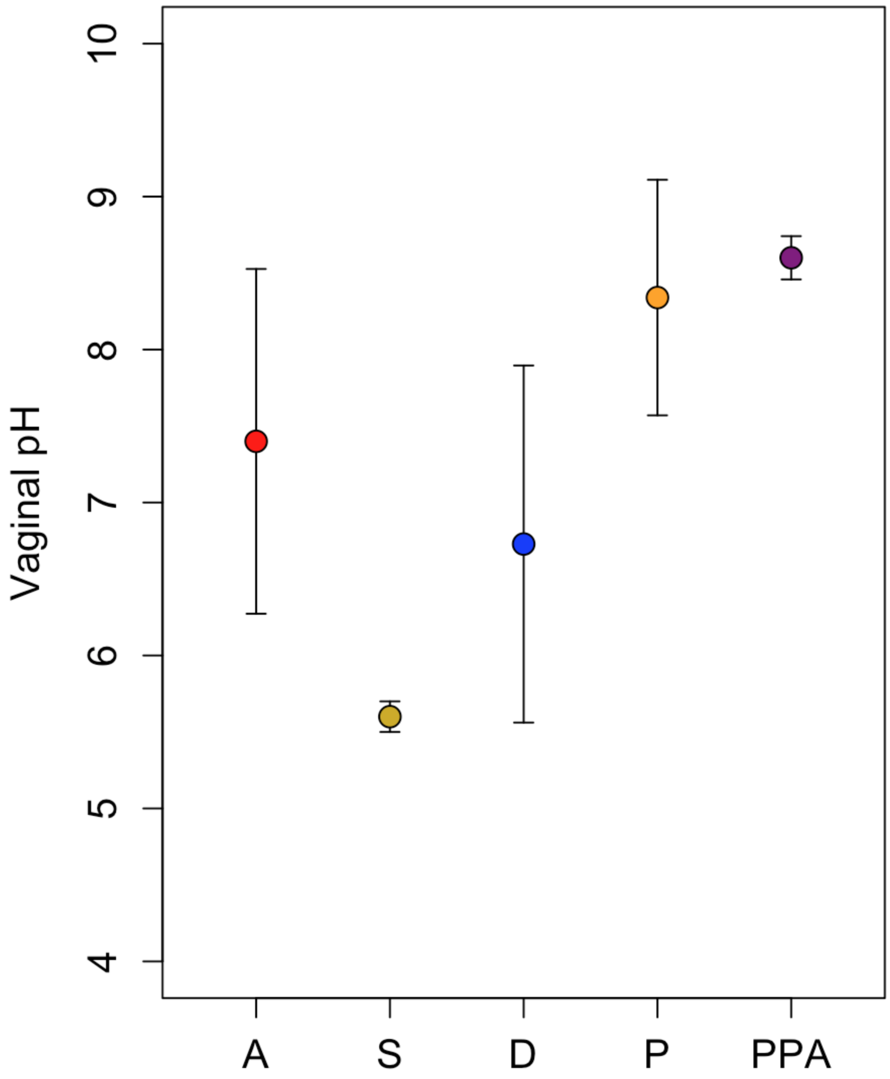

Vertical and horizontal transmission

```
relate1 <- relate
relate1[upper.tri(relate1, diag = TRUE)] <- NA
r_melt <- melt(relate1)
colnames(r_melt) <- c("sname1","sname2","r")
r_melt <- subset(r_melt, !is.na(r))
key <- data.frame(baboon_id=meta$baboon_id, sample_id=rownames(meta))
merge1 <- merge(key, r_melt, by.x="baboon_id", by.y="sname1", all.x=TRUE)
colnames(merge1)[1:2] <- c("baboon_id1", "sample_id1")
merge2 <- merge(key, merge1, by.x="baboon_id", by.y="sname2", all.x=TRUE)
colnames(merge2)[1:2] <- c("baboon_id2", "sample_id2")
parents <- data.frame(sample_id=rownames(meta), mother=meta$mother, father=meta
$father)
merge3 <- merge(parents, merge2, by.x="sample_id", by.y="sample_id1")
colnames(merge3)[1:3] <- c("sample_id1", "mom1", "dad1")
merge4 <- merge(parents, merge3, by.x="sample_id", by.y="sample_id2")
colnames(merge4)[1:3] <- c("sample_id2", "mom2", "dad2")
merge4$sibtype <- ifelse((merge4$mom1 == merge4$mom2) & (merge4$dad1 == merge4$
dad2), "full",
                    ifelse(merge4$mom1 == merge4$mom2, "mat",
                      ifelse(merge4$dad1 == merge4$dad2, "pat","not")))
merge4[is.na(merge4$sibtype),]$sibtype <- "not"
rep <- data.frame(rep=meta3$rep_cycle, row.names=rownames(meta3))
data1 <- merge(rep, merge4, by.x="row.names", by.y="sample_id1")
colnames(data1)[1:2] <- c("sample_id1", "rep1")
data2 <- merge(rep, data1, by.x="row.names", by.y="sample_id2")
colnames(data2)[1:2] <- c("sample_id2", "rep2")
data3 <- within(data2, {
  reptype <- ifelse(rep1 == rep2, "same","diff")
})
data4 <- merge(bray_melt, data3, by.x=c("sample_id1","sample_id2"), by.y=c("sam
ple_id1","sample_id2"))
data5 <- merge(unifrac_melt, data4, by.x=c("sample_id1","sample_id2"), by.y=c("
sample_id1","sample_id2"))
# Remove all pairs where the snames are equal (they will also be full sibs)
data6 <- subset(data5, baboon_id1 != baboon_id2)
# Remove all "not" sib pairs with r values >=0.125 (this means they are probabl
y mother/daughter pairs)
data7 <- subset(data6, !(sibtype == "not" & r >= 0.125))
# How many unique maternal sibling pairs?
nrow(unique(subset(data7, sibtype == "mat")[,c("baboon_id1","baboon_id2")]))
```

```
## [1] 10
```

```
# How many unique paternal sibling pairs?
nrow(unique(subset(data7, sibtype == "pat")[,c("baboon_id1","baboon_id2")]))
```

```
## [1] 15
```

```
# How many unique unrelated pairs?
nrow(unique(subset(data7, sibtype == "not")[,c("baboon_id1","baboon_id2")]))
```

```
## [1] 1049
```

```
# What is the average relatedness (r) of unrelated pairs?
# Mean
mean(unique(subset(data7, sibtype == "not")[,c("baboon_id1","baboon_id2","r")])
$r)
```

```
## [1] 0.004524398
```

```
# SD
sd(unique(subset(data7, sibtype == "not")[,c("baboon_id1","baboon_id2","r")])$r
)
```

```
## [1] 0.01477449
```

Do maternal siblings have more similar microbiomes than paternal siblings or non-related pairs?

```
# Remove the effect of rep. state
data7$reptype <- as.factor(data7$reptype)
lm_bray <- lm(data7$bray ~ data7$reptype)
resid_bray <- lm_bray$residuals
data8 <- data.frame(data7, resid_bray)
#Unifrac Weighted
lm_unifrac <- lm(data8$unifrac ~ data8$reptype)
resid_unifrac <- lm_unifrac$residuals
data9 <- data.frame(data8, resid_unifrac)
data9$sibtype <- as.factor(data9$sibtype)
data9$sibtype <- factor(data9$sibtype, levels = c("mat", "pat", "not"))
#Bray-Curtis
kruskal.test(data9$resid_bray ~ data9$sibtype)
```

```
##
##  Kruskal-Wallis rank sum test
##
## data:  data9$resid_bray by data9$sibtype
## Kruskal-Wallis chi-squared = 0.24966, df = 2, p-value = 0.8826
```

```
#Weighted Unifrac
kruskal.test(data9$resid_unifrac ~ data9$sibtype)
```

```
##
##  Kruskal-Wallis rank sum test
##
## data:  data9$resid_unifrac by data9$sibtype
## Kruskal-Wallis chi-squared = 0.77353, df = 2, p-value = 0.6793
```

No.

Does overall pairwise genetic relatedness between baboons explain similarity in vaginal microbial communities?

```
library(reshape2)
r_melt <- melt(relate)
colnames(r_melt) <- c("sname1","sname2","r")
key <- data.frame(baboon_id=meta$baboon_id, sample_id=rownames(meta))
merge1 <- merge(key, r_melt, by.x="baboon_id", by.y="sname1", all.x=TRUE)
colnames(merge1)[1:2] <- c("baboon_id1", "sample_id1")
merge2 <- merge(key, merge1, by.x="baboon_id", by.y="sname2", all.x=TRUE)
colnames(merge2)[1:2] <- c("baboon_id2", "sample_id2")
relate_m <- acast(merge2, sample_id1~sample_id2, value.var="r")
rep_m <-as.matrix(daisy(data.frame(rep=meta1$rep_cycle, row.names=rownames(meta
1)), metric="gower"))
bray1 <- bray[match(rownames(relate_m), rownames(bray)),match(rownames(relate_m
), colnames(bray))]
unifrac1 <- unifrac[match(rownames(relate_m), rownames(unifrac)),match(rownames
(relate_m), colnames(unifrac))]
rep_m <- rep_m[match(rownames(relate_m), rownames(rep_m)),match(rownames(relate
_m), colnames(rep_m))]
# Mantel tests
library(vegan)
mantel.partial(bray1, relate_m, rep_m, method="pearson", permutations=10000)
```

```
##
## Partial Mantel statistic based on Pearson's product-moment correlation
##
## Call:
## mantel.partial(xdis = bray1, ydis = relate_m, zdis = rep_m, method = "pearso
n",      permutations = 10000)
##
## Mantel statistic r: 0.01533
##       Significance: 0.30457
##
## Upper quantiles of permutations (null model):
##    90%    95%  97.5%    99%
## 0.0350 0.0436 0.0509 0.0597
## Permutation: free
## Number of permutations: 10000
```

```
mantel.partial(unifrac1, relate_m, rep_m, method="pearson", permutations=10000)
```

```
##
## Partial Mantel statistic based on Pearson's product-moment correlation
##
## Call:
## mantel.partial(xdis = unifrac1, ydis = relate_m, zdis = rep_m,      method =
 "pearson", permutations = 10000)
##
## Mantel statistic r: 0.01844
##       Significance: 0.26807
##
## Upper quantiles of permutations (null model):
##    90%    95%  97.5%    99%
## 0.0361 0.0458 0.0537 0.0626
## Permutation: free
## Number of permutations: 10000
```

No.

Does sharing sexual partners predict vaginal microbial similarity?

```r
# B5 (sample V5) and B30 (sample V17) are not included because they don't have
any consorts (too young)
consort_bray <- as.matrix(vegdist(history, method="bray", binary=FALSE, diag=TR
UE, upper=TRUE))
rep_m1 <- rep_m[rownames(rep_m) %in% rownames(consort_bray), colnames(rep_m) %i
n% rownames(consort_bray)]
rep_m1 <- rep_m1[match(rownames(consort_bray), rownames(rep_m1)),match(rownames
(consort_bray), colnames(rep_m1))]
bray_ss <- bray[rownames(bray) %in% rownames(consort_bray), colnames(bray) %in%
 rownames(consort_bray)]
bray_ss <- bray1[match(rownames(consort_bray), rownames(bray1)),match(rownames(
consort_bray), colnames(bray1))]
unifrac_ss <- unifrac[rownames(unifrac) %in% rownames(consort_bray), colnames(u
nifrac) %in% rownames(consort_bray)]
unifrac_ss <- unifrac1[match(rownames(consort_bray), rownames(unifrac1)),match(
rownames(consort_bray), colnames(unifrac1))]
mantel.partial(bray_ss, consort_bray, rep_m1, method="pearson", permutations=10
000)
```

```
##
## Partial Mantel statistic based on Pearson's product-moment correlation
##
## Call:
## mantel.partial(xdis = bray_ss, ydis = consort_bray, zdis = rep_m1,      meth
od = "pearson", permutations = 10000)
##
## Mantel statistic r: 0.0515
##       Significance: 0.044696
##
## Upper quantiles of permutations (null model):
##    90%    95%  97.5%    99%
## 0.0377 0.0496 0.0599 0.0731
## Permutation: free
## Number of permutations: 10000
```

```r
mantel.partial(unifrac_ss, consort_bray, rep_m1, method="pearson", permutations
=10000)
```

```
##
## Partial Mantel statistic based on Pearson's product-moment correlation
##
## Call:
## mantel.partial(xdis = unifrac_ss, ydis = consort_bray, zdis = rep_m1,      m
ethod = "pearson", permutations = 10000)
##
## Mantel statistic r: 0.04339
##       Significance: 0.065493
##
## Upper quantiles of permutations (null model):
##    90%    95%  97.5%    99%
## 0.0364 0.0477 0.0594 0.0732
## Permutation: free
## Number of permutations: 10000
```

Yes!

Does individual identity predict vaginal microbial similarity?

```
bid <- data.frame(baboon_id=meta[,"baboon_id"], row.names=row.names(meta))
bid_m <- as.matrix(daisy(bid, metric="gower"))
bray1 <- bray[match(rownames(bid_m), rownames(bray)),match(rownames(bid_m), col
names(bray))]
unifrac1 <- unifrac[match(rownames(bid_m), rownames(unifrac)),match(rownames(bi
d_m), colnames(unifrac))]
rep_m <- rep_m[match(rownames(bid_m), rownames(rep_m)),match(rownames(bid_m), c
olnames(rep_m))]
mantel.partial(bray1, bid_m, rep_m, method="pearson", permutations=10000)
```

```
##
## Partial Mantel statistic based on Pearson's product-moment correlation
##
## Call:
## mantel.partial(xdis = bray1, ydis = bid_m, zdis = rep_m, method = "pearson",
      permutations = 10000)
##
## Mantel statistic r: 0.03127
##       Significance: 0.13289
##
## Upper quantiles of permutations (null model):
##    90%    95%  97.5%    99%
## 0.0364 0.0477 0.0578 0.0697
## Permutation: free
## Number of permutations: 10000
```

```
mantel.partial(unifrac1, bid_m, rep_m, method="pearson", permutations=10000)
```

```
##
## Partial Mantel statistic based on Pearson's product-moment correlation
##
## Call:
## mantel.partial(xdis = unifrac1, ydis = bid_m, zdis = rep_m, method = "pearso
n",      permutations = 10000)
##
## Mantel statistic r: 0.0235
##       Significance: 0.19098
##
## Upper quantiles of permutations (null model):
##    90%    95%  97.5%    99%
## 0.0343 0.0439 0.0526 0.0626
## Permutation: free
## Number of permutations: 10000
```

No.

Does similarity in age predict vaginal microbial similarity?

```
age <- data.frame(age=meta[,"age"], row.names=row.names(meta))
age.dist <- as.matrix(dist(age, method = "manhattan", upper=TRUE, diag=TRUE))
bray1 <- bray[match(rownames(age.dist), rownames(bray)),match(rownames(age.dist
), colnames(bray))]
unifrac1 <- unifrac[match(rownames(age.dist), rownames(unifrac)),match(rownames
(age.dist), colnames(unifrac))]
rep_m <- rep_m[match(rownames(age.dist), rownames(rep_m)),match(rownames(age.di
st), colnames(rep_m))]
mantel.partial(bray1, age.dist, rep_m, method="pearson", permutations=10000)
```

```
##
## Partial Mantel statistic based on Pearson's product-moment correlation
##
## Call:
## mantel.partial(xdis = bray1, ydis = age.dist, zdis = rep_m, method = "pearso
n",      permutations = 10000)
##
## Mantel statistic r: -0.04683
##       Significance: 0.74893
##
## Upper quantiles of permutations (null model):
##    90%    95%  97.5%    99%
## 0.0848 0.1118 0.1345 0.1639
## Permutation: free
## Number of permutations: 10000
```

```
mantel.partial(unifrac1, age.dist, rep_m, method="pearson", permutations=10000)
```

```
##
## Partial Mantel statistic based on Pearson's product-moment correlation
##
## Call:
## mantel.partial(xdis = unifrac1, ydis = age.dist, zdis = rep_m,      method =
 "pearson", permutations = 10000)
##
## Mantel statistic r: -0.0451
##       Significance: 0.72353
##
## Upper quantiles of permutations (null model):
##    90%    95%  97.5%    99%
## 0.0922 0.1253 0.1514 0.1813
## Permutation: free
## Number of permutations: 10000
```

No.

Does social group co-residency predict vaginal microbial similarity?

```
grp <- data.frame(grp=meta[,"social_grp"], row.names=row.names(meta))
grp.dist <- as.matrix(daisy(grp, metric="gower"))

bray1 <- bray[match(rownames(grp.dist), rownames(bray)),match(rownames(grp.dist
), colnames(bray))]
unifrac1 <- unifrac[match(rownames(grp.dist), rownames(unifrac)),match(rownames
(grp.dist), colnames(unifrac))]
rep_m <- rep_m[match(rownames(grp.dist), rownames(rep_m)),match(rownames(grp.di
st), colnames(rep_m))]
mantel.partial(bray1, grp.dist, rep_m, method="pearson", permutations=10000)
```

```
##
## Partial Mantel statistic based on Pearson's product-moment correlation
##
## Call:
## mantel.partial(xdis = bray1, ydis = grp.dist, zdis = rep_m, method = "pearso
n",      permutations = 10000)
##
## Mantel statistic r: 0.01359
##       Significance: 0.26707
##
## Upper quantiles of permutations (null model):
##    90%    95%  97.5%    99%
## 0.0327 0.0438 0.0540 0.0654
## Permutation: free
## Number of permutations: 10000
```

```
mantel.partial(unifrac1, grp.dist, rep_m, method="pearson", permutations=10000)
```

```
##
## Partial Mantel statistic based on Pearson's product-moment correlation
##
## Call:
## mantel.partial(xdis = unifrac1, ydis = grp.dist, zdis = rep_m,      method =
 "pearson", permutations = 10000)
##
## Mantel statistic r: 0.008199
##       Significance: 0.34287
##
## Upper quantiles of permutations (null model):
##    90%    95%  97.5%    99%
## 0.0330 0.0444 0.0561 0.0707
## Permutation: free
## Number of permutations: 10000
```

No.
